# Depletion of WFS1 compromises mitochondrial function in hiPSC-derived neuronal models of Wolfram syndrome

**DOI:** 10.1016/j.stemcr.2023.04.002

**Published:** 2023-05-09

**Authors:** Malgorzata Zatyka, Tatiana R. Rosenstock, Congxin Sun, Adina M. Palhegyi, Georgina W. Hughes, Samuel Lara-Reyna, Dewi Astuti, Alessandro di Maio, Axel Sciauvaud, Miriam E. Korsgen, Vesna Stanulovic, Gamze Kocak, Malgorzata Rak, Sandra Pourtoy-Brasselet, Katherine Winter, Thiago Varga, Margot Jarrige, Hélène Polvèche, Joao Correia, Eva-Maria Frickel, Maarten Hoogenkamp, Douglas G. Ward, Laetitia Aubry, Timothy Barrett, Sovan Sarkar

**Affiliations:** 1 Institute of Cancer and Genomic Sciences, Institute of Biomedical Research, College of Medical and Dental Sciences, University of Birmingham, Edgbaston, Birmingham B15 2TT, UK; 2 Institute of Microbiology and Infection, University of Birmingham, Birmingham B15 2TT, UK; 3 Tech Hub Microscopy Facility, College of Medical and Dental Sciences, University of Birmingham, Birmingham B15 2TT, UK; 4 INSERM UMR 861, I-STEM, AFM, 91100 Corbeil-Essonnes, France; 5 Université Paris-Saclay, INSERM, University Evry, Institut des cellules Souches pour le Traitement et l’Etude des maladies Monogéniques, 91100 Corbeil-Essonnes, France; 6 Université Paris Cité, INSERM, NeuroDiderot, 75019 Paris, France; 7 CECS/AFM, I-STEM, 91100 Corbeil-Essonnes, France; 8 COMPARE Advanced Imaging Facility, College of Medical and Dental Sciences, University of Birmingham, Birmingham B15 2TT, UK; 9 Department of Endocrinology, Birmingham Women’s and Children’s Hospital, Steelhouse Lane, Birmingham B4 6NH, UK

**Keywords:** Wolfram syndrome, WFS1, Mitochondrial dysfunction, Mitochondrial membrane potential, VDAC1, Mitochondria-associated ER membrane, Human induced pluripotent stem cell-derived neurons, Neurodegeneration, Cyclosporin A, MnTBAP

## Abstract

Mitochondrial dysfunction involving mitochondria-associated ER membrane (MAM) dysregulation is implicated in the pathogenesis of late-onset neurodegenerative diseases, but understanding is limited for rare early-onset conditions. Loss of the MAM-resident protein WFS1 causes Wolfram syndrome (WS), a rare early-onset neurodegenerative disease that has been linked to mitochondrial abnormalities. Here we demonstrate mitochondrial dysfunction in human induced pluripotent stem cell-derived neuronal cells of WS patients. VDAC1 is identified to interact with WFS1, whereas loss of this interaction in WS cells could compromise mitochondrial function. Restoring WFS1 levels in WS cells reinstates WFS1-VDAC1 interaction, which correlates with an increase in MAMs and mitochondrial network that could positively affect mitochondrial function. Genetic rescue by WFS1 overexpression or pharmacological agents modulating mitochondrial function improves the viability and bioenergetics of WS neurons. Our data implicate a role of WFS1 in regulating mitochondrial functionality and highlight a therapeutic intervention for WS and related rare diseases with mitochondrial defects.

## Highlights

• Wolfram syndrome patient-derived neuronal cells exhibit mitochondrial dysfunction
• Loss of WFS1 interaction with VDAC1 could potentially affect mitochondrial function
• Mitochondrial and cell death phenotypes are rescued by genetic correction
• Pharmacological agents rescue mitochondrial defects and improve neuronal viability

## Graphical abstract

**Figure:**
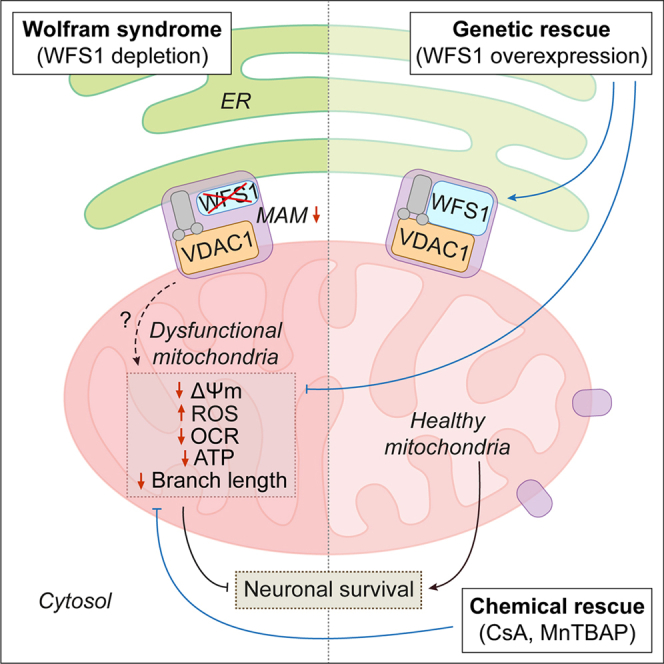

## Teaser

In this article, Sarkar and colleagues describe mitochondrial dysfunction in neuronal cells generated from patient-derived iPSCs of Wolfram syndrome, a rare early-onset neurodegenerative disorder. Genetic rescue by WFS1 restoration or chemical rescue by drugs modulating mitochondrial function improves the bioenergetics and viability of Wolfram syndrome patient neurons, thus providing insights for therapeutic intervention.

## Introduction

Neurodegenerative diseases are characterized by gradual loss of neuronal function and viability. Multiple studies have demonstrated a crucial role of impairment of mitochondrial homeostasis in the pathogenesis of common neurodegenerative diseases like Alzheimer’s disease (AD) and Parkinson’s disease (PD) and in certain rare neurodegenerative diseases (Johri and Beal, 2012). Mitochondria perform essential cellular functions in the regulation of bioenergetics, ion homeostasis, metabolism, and apoptosis and form dynamic networks that make contacts with other cellular organelles (Nunnari and Suomalainen, 2012). The interaction domains between mitochondria and endoplasmic reticulum (ER) are known as mitochondria-associated ER membranes (MAMs), which are involved in the regulation of mitochondrial biogenesis and dynamics, Ca^2+^ transfer, phospholipid synthesis and exchange, and cell death (Delprat et al., 2018; Giorgi et al., 2015). Dysfunction of MAM has been implicated in the pathogenesis of various neurodegenerative diseases including AD and PD (Paillusson et al., 2016). One of the proteins in MAM was found to be WFS1 (Wolfram syndrome 1; also called wolframin) (Delprat et al., 2018; Horner et al., 2015; La Morgia et al., 2020; Poston et al., 2013; Zhang et al., 2011), loss of function of which causes a rare, early-onset neurodegenerative disorder called Wolfram syndrome (WS) with no effective cure (Abreu and Urano, 2019; Barrett et al., 1995; Inoue et al., 1998). Loss of WFS1 protein in WS is associated with brain and optic nerve atrophy, diabetes, deafness, psychosis, and depression (Barrett et al., 1995; Rigoli et al., 2018), and these neurological and psychiatric defects resemble mitochondrial disease-like symptoms (Bu and Rotter, 1993).

Multiple lines of evidence arising from studies in immortalized, non-human or non-clinical cell lines suggest that mitochondrial functionality could be affected in WS associated with *WFS1* mutations. In immortalized human embryonic kidney (HEK) cells, siRNA-mediated *WFS1* knockdown caused upregulation of genes related to mitochondrial damage (Koks et al., 2013). Similarly, downregulation of WFS1 in primary cortical neurons of rat with shRNA-mediated *Wfs1* knockdown or of *Wfs1*-deficient mice impaired mitochondrial dynamics, which was associated with perturbation in neuronal function (Cagalinec et al., 2016). The underlying mechanism of alteration in mitochondrial dynamics was suggested to be arising from ER stress-mediated dysregulation of Ca^2+^ homeostasis (Cagalinec et al., 2016). A subsequent study using WS patient fibroblasts has further shown that loss of WFS1 disrupted its association with the neuronal calcium sensor 1 (NCS1) and inositol 1,4,5-trisphosphate receptor (IP_3_R), which in turn diminished Ca^2+^ transfer between ER and mitochondria to cause mitochondrial deregulation (Angebault et al., 2018). In this study, WS patient fibroblasts exhibited reduction in mitochondrial respiration and complex I activity, but mitochondrial membrane potential (ΔΨ_m_) was unaffected (Angebault et al., 2018). However, another study in WS patient fibroblasts reported no changes in mitochondrial respiration, ΔΨ_m_, or network morphology (La Morgia et al., 2020). On the contrary, increased mitochondrial bioenergetics were reported in the quadriceps muscle fibers of *Wfs1*-deficient mice (Eimre et al., 2018), which also had higher basal oxygen consumption (Ehrlich et al., 2016). Apart from WFS1, mutations in another MAM-resident protein CISD2 associated with Wolfram syndrome type 2 caused mitochondrial abnormalities in mouse models of *Cisd2* deficiency (Chen et al., 2009; Delprat et al., 2018; Wiley et al., 2013). Moreover, mitochondrial DNA deletions were found in some WS patients (Barrientos et al., 1996). Overall, these studies show contradictory mitochondrial functionality with varied phenotypic readouts in WS in a context-dependent manner, and therefore it is pertinent to study mitochondrial function in patient-derived disease-affected cells for biomedical exploitation.

Here we investigated the impact of loss of WFS1 protein on mitochondria-associated gene expression and mitochondrial function, and its potential mechanism and consequence on cell survival, in neural stem cells (NSCs) and cortical neurons differentiated from WS patient-derived human induced pluripotent stem cells (hiPSCs). Such patient hiPSC-derived neuronal models potentially serve as pre-clinical, disease-relevant cellular platforms for studying disease mechanisms and identifying drug candidates (Avior et al., 2016). In this context, we also assessed the therapeutic efficacy of pharmacological agents modulating mitochondrial function on neuronal viability.

## Results

### Deregulation of mitochondria-associated genes in WS hiPSC-derived neuronal cells

**Figure 1:**
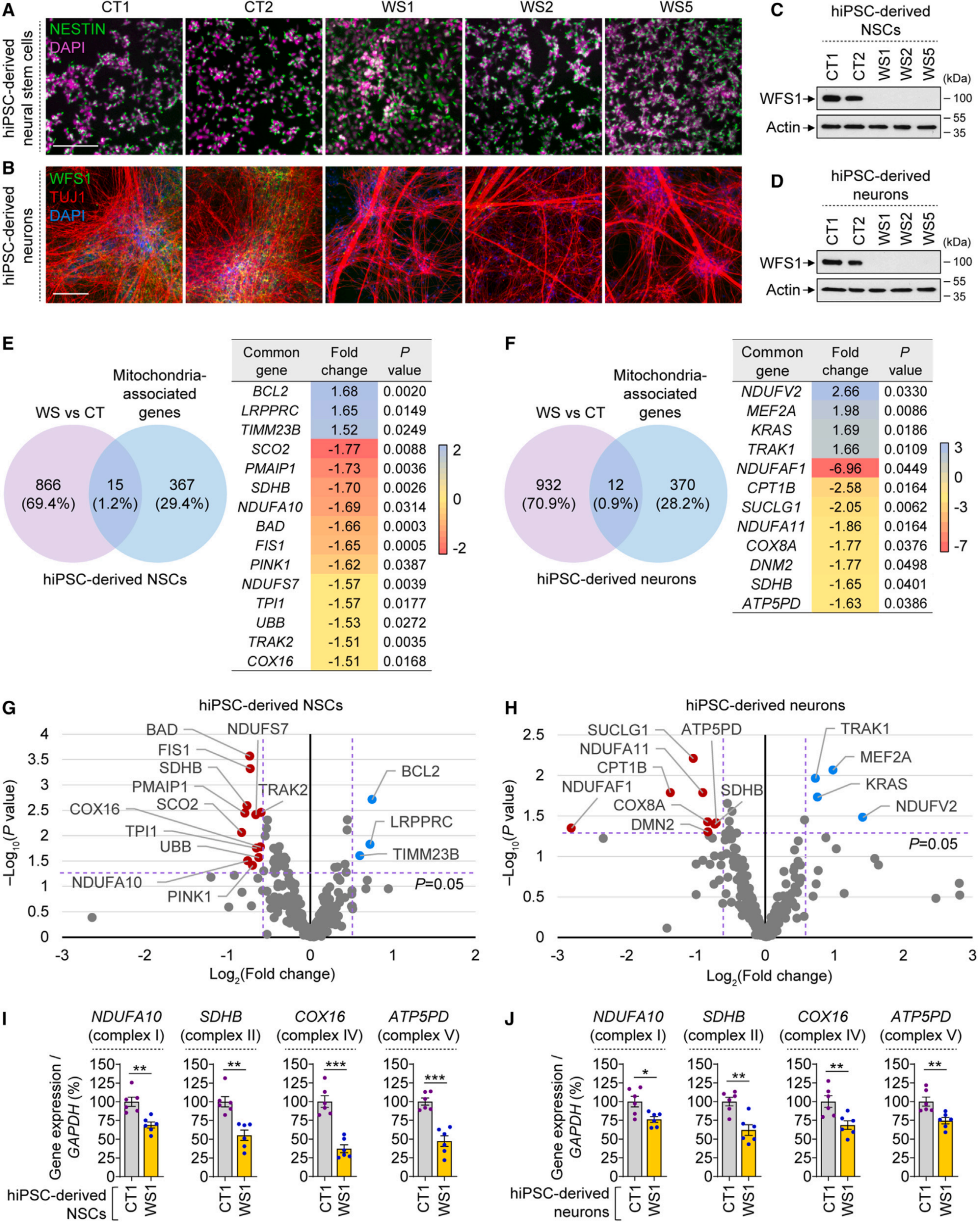
Deregulation of mitochondrial genes in WS patient hiPSC-derived NSCs and neurons (A–D) Immunofluorescence images of NESTIN (A), WFS1 and TUJ1 (B), and immunoblotting analysis of WFS1 (C, D) in control (CT1, CT2) and WS patient (WS1, WS2, WS5) hiPSC-derived NSCs (A, C) and 4-week-old (4 w) neurons (B, D). (E and F) Venn diagram summarizing the number of differentially expressed genes (DEGs) specific or common to mitochondria-associated genes set in NSCs (E) or neurons (4 w; F), and table of mitochondria-associated genes significantly deregulated in WS NSCs (E) and neurons (4 w; F), compared with CT. (G–J) Volcano plot representation of mitochondria-associated genes analyzed by RNA-seq (G, H; thresholds shown as dashed purple lines), and qPCR expression analyses of *NDUFA10*, *SHDB*, *COX16*, and *ATP5PD* relative to *GAPDH* (I, J), in CT and WS hiPSC-derived NSCs (G, I) and neurons (4 w; H, J). Graphical data are mean ± SEM of n = 6 biological replicates (I, J). p values were calculated by unpaired two-tailed Student’s t test on three independent experiments (I, J). DEGs were identified via Partek gene-specific analysis (GSA) algorithm (p value ≤ 5%; fold change ≥ 1.5; minimum reads > 100) (E, F). ^∗^p < 0.05; ^∗∗^p < 0.01; ^∗∗∗^p < 0.001. Scale bar, 100 μm (A, B). See also Figure S1.

To study mitochondrial function in clinically relevant WS cellular platforms, we generated NSCs and neurons from previously established hiPSC lines derived from three WS patients (WS1, WS2, WS5) along with two healthy individuals as controls (CT1, CT2) (Boissart et al., 2013; Pourtoy-Brasselet et al., 2021; Shang et al., 2014) (Figure S1A). Cellular identities of hiPSC-derived cells were confirmed by immunofluorescence and gene expression analyses of cell-specific markers, such as NESTIN and PAX6 for NSCs, and TUJ1, MAP2, and NeuN for neurons (Figures 1A, 1B, and S1B–S1D). Our neuronal differentiation method generated neurons of cortical nature (Pourtoy-Brasselet et al., 2021), as evident from the gene expression of *POU3F2*, *CUX1*, and *TBR1* that are specific for cortical neurons and by TBR1 immunostaining in TUJ1^+^ neurons (Figures S1E and S1F). Since disease-associated mutations in *WFS1* cause the protein to be unstable and degraded by the proteasome (Guo et al., 2011), WFS1 was detected only in control NSCs and neurons but not in WS cells (Figures 1B–1D).

In order to study the implication of mitochondria in WS pathogenesis, we established a list of 382 mitochondria-associated genes and compared the expression of these genes in three CT (control) and three WS hiPSC-derived NSCs and neurons using our previously published RNA-seq datasets (Pourtoy-Brasselet et al., 2021). Among 881 and 944 differentially expressed genes (DEGs) identified in WS compared with CT respectively in NSCs and neurons, a number of mitochondria-associated DEGs were found in WS NSCs and neurons (p value ≤ 5%; fold change ≥ 1.5; minimum reads > 100) (Figures 1E and 1F). Among the mitochondria gene set, there were 15 DEGs in WS NSCs (3 upregulated, 12 downregulated) and 12 DEGs in WS neurons (4 upregulated, 8 downregulated) compared with the control cells (Figures 1E and 1F). These mitochondria-associated DEGs in NSCs and neurons were also depicted in a volcano plot by plotting the magnitude of change against the measure of significance (Figures 1G and 1H).

Strikingly, various mitochondria-associated DEGs linked to mitochondrial electron transport chain (ETC) complexes were downregulated in WS NSCs and neurons compared with their control counterparts (Figures 1E–1H). These results were confirmed by qPCR for a set of mitochondrial ETC complex genes that were identified in either or both NSC and neuron datasets (Figures 1I and 1J). These genes include *NDUFA10* (complex I), *SDHB* (complex II), *COX16* (complex IV), and *ATP5PD* (complex V). Indeed, significant reduction in the expression of these mitochondrial ETC complex genes was found in WS1 NSCs and neurons compared with CT1 cells (Figures 1I and 1J). These data suggest that there are deficits in mitochondrial ETC complexes that could ultimately lead to mitochondrial deregulation in WS neuronal cells.

### Mitochondrial dysfunction in WS hiPSC-derived neuronal cells

**Figure 2:**
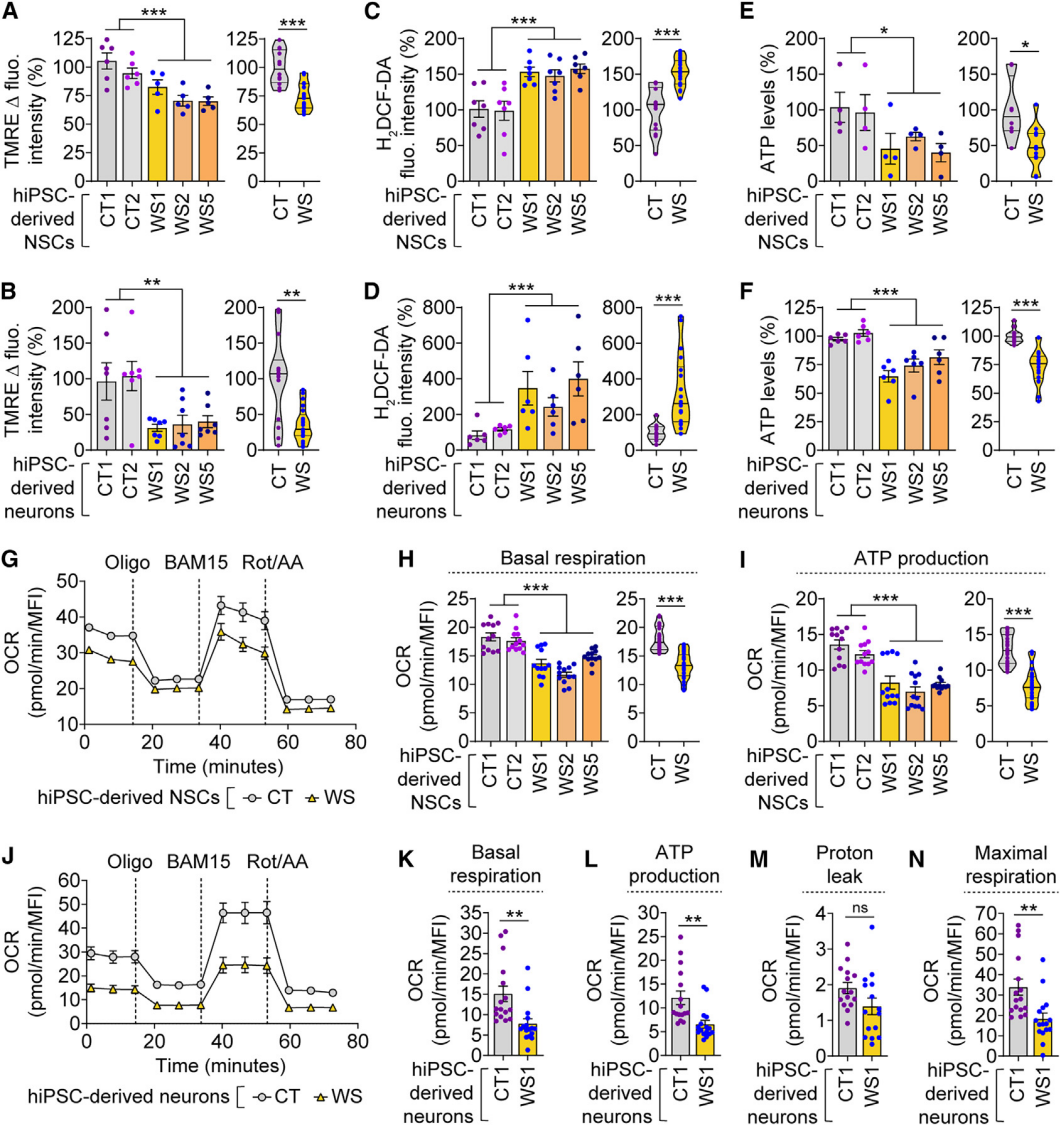
Mitochondrial dysfunction in WS patient hiPSC-derived NSCs and neurons (A–F) Measurements of ΔΨm by TMRE Δ fluorescence intensity (A, B), ROS by H_2_DCF-DA fluorescence intensity (C, D), and ATP levels (E, F) in CT1, CT2, WS1, WS2, and WS5 hiPSC-derived NSCs (A, C, E) and neurons (4 w; B, D, F). (G–N) Oxygen consumption rate (OCR) levels (G, J) were measured post mitochondrial stress test, involving oligomycin (Oligo), BAM15, and rotenone (Rot)/antimycin A (AA) treatment, in hiPSC-derived NSCs (in CT1, CT2, WS1, WS2, and WS5; G–I) and neurons (4 w; in CT1 and WS1; J–N). Basal respiration (H, K), ATP production (I, L), proton leak (M), and maximal respiration (N) were calculated as described in experimental procedures and Table S6. MFI: mean fluorescent intensity. Graphical data are mean ± SEM of n = 4–16 biological replicates as indicated (A–N) or displayed as violin plots (line at median) of CT and WS groups (A–F, H, I). p values were calculated by unpaired two-tailed Student’s t test on two (A–F) or three (G–N) independent experiments. ^∗^p < 0.05; ^∗∗^p < 0.01; ^∗∗∗^p < 0.001; ns, non-significant. See also Figure S2.

Since perturbations in mitochondrial ETC complexes can alter mitochondrial membrane potential (ΔΨ_m_) (Zorova et al., 2018), we analyzed mitochondrial functionality in NSCs and neurons derived from multiple CT and WS hiPSC lines. Measurement of ΔΨ_m_ was performed using tetramethylrhodamine ethyl ester (TMRE), a positively charged dye sequestered by negatively charged active mitochondria (Rosenstock et al., 2022). WS NSCs and neurons exhibited lower ΔΨ_m_ compared with the respective CT cells (Figures 2A and 2B), indicating mitochondrial depolarization in WS cells. Since this is often associated with oxidative stress (Murphy, 2009), we assessed intracellular reactive oxygen species (ROS) levels using a cell-permeable fluorescent dye, H_2_DCF-DA (Rosenstock et al., 2022). Indeed, WS NSCs and neurons had elevated ROS levels compared with their CT counterparts (Figures 2C and 2D). Likely because of mitochondrial deregulation, ATP levels were decreased in WS NSCs and neurons in comparison with the respective CT cells (Figures 2E and 2F). However, inconsistent changes were observed for mitochondrial Ca^2+^ in WS NSCs and neurons compared with their respective controls (Figures S2A and S2B), as measured by Fluo-3 AM Ca^2+^ indicator (Rosenstock et al., 2022). While WS NSCs showed a reduction in mitochondrial Ca^2+^ compared with CT NSCs despite variability between the individual WS lines, no significant difference was found between WS and CT neurons (Figures S2A and S2B). These data demonstrate increased oxidative stress and improper ΔΨ_m_ in WS NSCs and neurons, and such a phenotype could be a contributing factor to neurodegeneration (Nunnari and Suomalainen, 2012).

We next analyzed mitochondrial respiration by measuring oxygen consumption rate (OCR) in two CT and three WS NSCs. The OCR levels of WS NSCs were lower than CT NSCs (Figure 2G). We evaluated key parameters of mitochondrial function by sequential addition of oligomycin, BAM15, and rotenone/antimycin A, which respectively inhibit ATP synthase, disrupt ΔΨ_m_, and block mitochondrial respiration (Kenwood et al., 2014). Basal respiration and ATP production were diminished in WS NSCs compared with CT NSCs (Figures 2H and 2I), but there were no significant differences in maximal respiration or proton leak (Figures S2C and S2D). These mitochondrial respiratory defects were further seen in WS1 neurons where maximal respiration was also decreased (Figures 2J–2N). It is possible that WS being associated with neurodegeneration, the mitochondrial phenotypes could be aggravated in neurons. Moreover, decreased ATP production in WS NSCs and neurons was consistent with lower ATP levels in these cells (Figures 2E, 2F, 2I, and 2L). Overall, these data suggest mitochondrial dysfunction in WS NSCs and neurons as shown by compromised mitochondrial respiration, lower ATP production, decreased ΔΨ_m_, and increased ROS.

### Genetic rescue of mitochondrial phenotype by restoration of WFS1 expression

**Figure 3:**
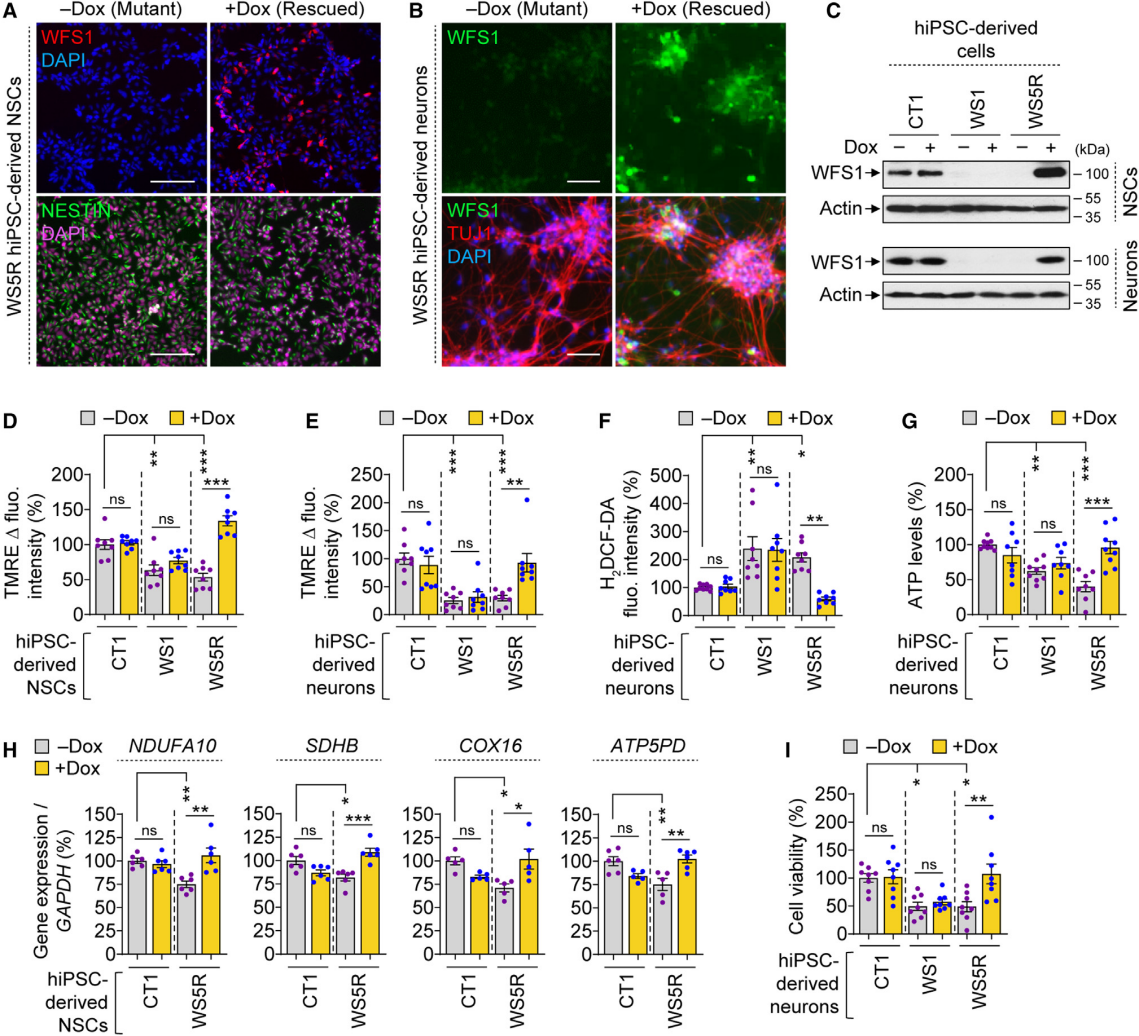
Genetic rescue of mitochondrial phenotypes and cell viability upon WFS1 restoration in WS patient hiPSC-derived cells (A–C) Immunofluorescence images of WFS1 (A, B), NESTIN (A), and TUJ1 (B) and immunoblotting analysis of WFS1 (C) in CT1, WS1, and WS5R hiPSC-derived NSCs (A, C) and neurons (4 w; B, C), treated with or without 50 ng/mL doxycycline (Dox) for 48 h. (D–I) Measurements of ΔΨm by TMRE Δ fluorescence intensity (D, E), ROS by H_2_DCF-DA fluorescence intensity (F), ATP levels (G), mitochondrial gene expression (H), and cell viability (I) in CT1, WS1, and WS5R hiPSC-derived NSCs (D, H) and neurons (4 w; E–G, I), treated with or without 50 ng/mL Dox for 48 h. Graphical data are mean ± SEM of n = 5–9 biological replicates as indicated (D–I). p values were calculated by one-way ANOVA with Tukey’s multiple comparisons test on three independent experiments (D–I). ^∗^p < 0.05; ^∗∗^p < 0.01; ^∗∗∗^p < 0.001; ns, non-significant. Scale bar, 50 (B) or 100 (A) μm. See also Figures S3 and S4.

To investigate the role of WFS1 in regulating mitochondrial function, we studied whether restoration of wild-type WFS1 affected the mitochondrial phenotypes in WS neuronal cells. We used WS5R “rescued” hiPSC line wherein *WFS1* cDNA under a doxycycline (Dox) inducible promoter was introduced via CRISPR-Cas9-mediated knockin at the *AAVS1* locus in WS patient-derived WS5 hiPSC line (Pourtoy-Brasselet et al., 2021). There were no off-target effects in WS5R hiPSCs due to genome editing, as confirmed by sequencing of the top five possible off-target loci predicted by CRISPOR (Figures S3A–S3E). In this “rescued” system, WS5R hiPSC-derived cells resemble WS patient-derived (mutant) line in the absence of Dox but act as a corrected (rescued) line in the presence of Dox due to WFS1 restoration (Figure S4A). Dox treatment restored WFS1 protein level, as confirmed by immunostaining and immunoblotting, in WS5R hiPSC-derived NSCs and neurons that also expressed the cell-specific markers (Figures 3A–3C and S4B).

In the absence of Dox, WS5R NSCs and neurons (mutant condition) exhibited a reduction in ΔΨ_m_ compared with the respective CT1 cells (control condition); a phenotype similar to WS1 cells (Figures 3D and 3E). However, Dox treatment (rescued condition) restored ΔΨ_m_ in WS5R cells but had no significant effect in CT1 or WS1 cells (Figures 3D and 3E). This suggests that restoration of ΔΨ_m_ is due to WFS1 expression and not because of WFS1-independent effects of Dox. Accordingly, increased ROS levels in WS5R cells was reduced by Dox (Figures 3F and S4C), but this rescue effect was not seen in CT1 or WS1 cells (Figure 3F). Likewise, low ATP levels in WS5R cells were increased by Dox (Figures 3G and S4D), which had no significant effect in CT1 or WS1 cells (Figure 3G). Moreover, the reduction in mitochondria-associated DEG expression linked to mitochondrial ETC complexes such as *NDUFA10*, *SDHB*, *COX16*, and *ATP5PD*, as seen in WS1 NSCs compared with CT1 NSCs, was also found in WS5R NSCs in the absence of Dox (Figures 1I and 3H). Treatment with Dox restored mitochondrial gene expression in WS5R NSCs to levels comparable to that in CT1 cells without causing any significant changes in CT1 cells (Figure 3H).

Since loss of ΔΨ_m_, elevation in ROS, and reduction in ATP levels could be detrimental to the cells (Nunnari and Suomalainen, 2012), we further analyzed cell viability in WS patient-derived cells and the effects of WFS1 restoration on them. Indeed, cell viability was significantly reduced in WS1 and WS5R neurons compared with CT1 neurons in the absence of Dox (Figure 3I). Treatment with Dox, which restored WFS1 level only in WS5R neurons, improved cell viability in these cells but not in WS1 neurons lacking the transgene or in CT1 neurons (Figures 3C and 3I). Thus, Dox-induced restoration of WFS1, but not any other direct effects of Dox, is attributed to the cytoprotective effect in WS neurons. Similarly, Dox-induced WFS1 expression significantly improved cell viability in WS5R NSCs (Figures 3C and S4E). Collectively, these data suggest that restoration of WFS1 levels rescues the mitochondrial and cell death phenotypes in WS neuronal cells.

### Identification of VDAC1 as WFS1 interactor and loss of this interaction in WS cells

**Figure 4:**
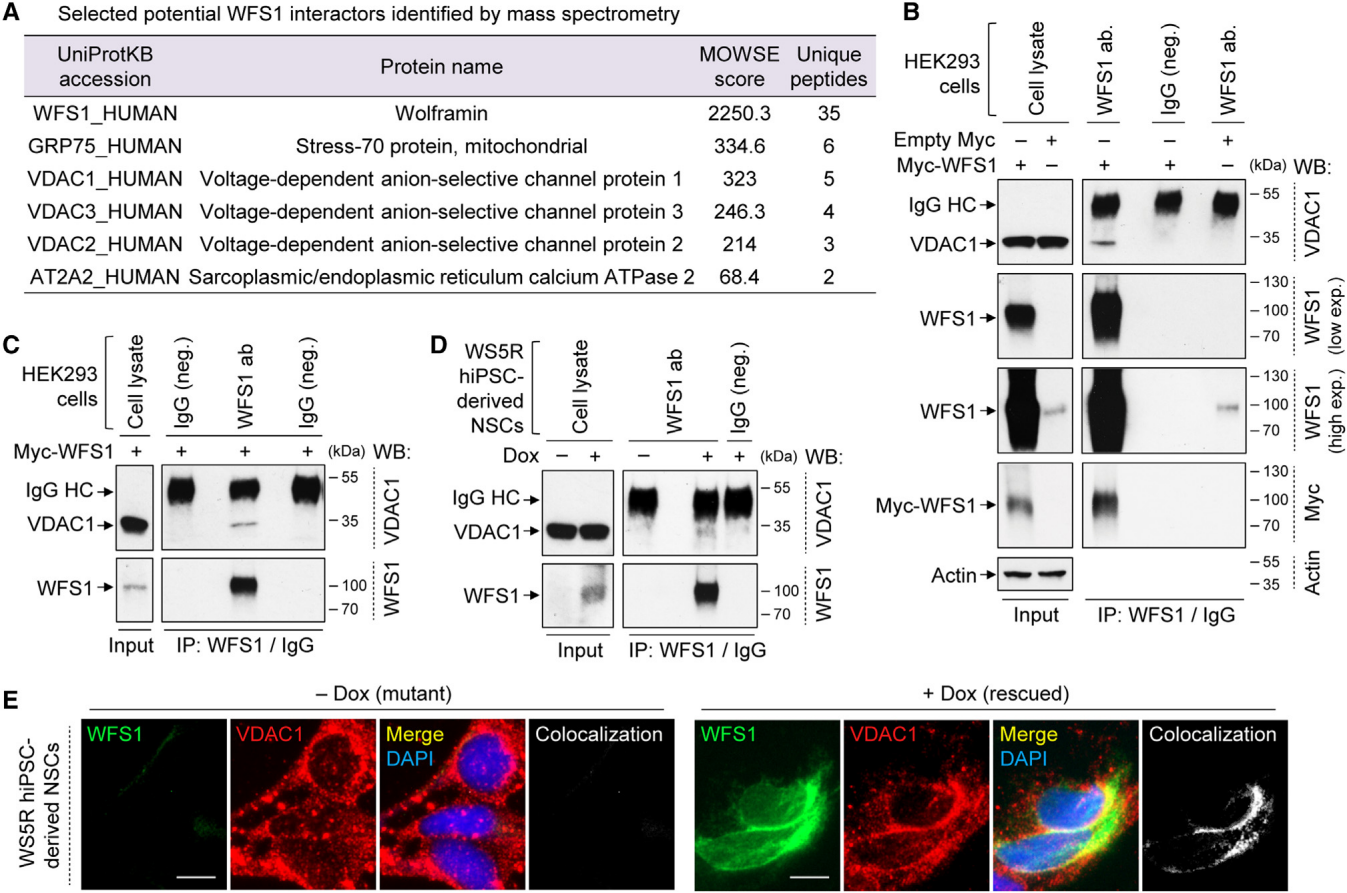
Identification of WFS1-VDAC1 interaction (A) Table of selected known and novel interactors of WFS1 identified by mass spectrometry after immunoprecipitation (IP) with WFS1 antibody in HEK293 cells expressing Myc-WFS1. (B) Immunoblotting analysis of VDAC1, WFS1, and Myc in cell lysates (input) or after IP with WFS1 antibody or IgG (negative control) in HEK293 cells, transfected with empty-Myc or Myc-WFS1. Detection of rabbit IgG heavy chain (IgG HC) with anti-mouse light chain secondary antibody was likely due to cross-reactivity. High and low exposures (exp.) of WFS1 IP and input immunoblots are shown. WB: western blot. (C and D) Immunoblotting analyses of VDAC1 and WFS1, after IP with WFS1 antibody or IgG (negative control), in the input (cell lysate) and IP samples of HEK293 cells transfected with Myc-WFS1 (C) or of WS5R hiPSC-derived NSCs treated with or without 50 ng/mL Dox for 48 h (D). (E) Immunofluorescence images of VDAC1 and WFS1 colocalization in WS5R hiPSC-derived NSCs, treated with or without 50 ng/mL Dox for 48 h. Scale bar, 10 μm (E). See also Figure S5.

To elucidate the potential mechanism underlying mitochondrial dysfunction in WS cells, we took an unbiased approach to identify WFS1 interactors via immunoprecipitation (IP) and liquid chromatography with tandem mass spectrometry (LC-MS/MS). Protein lysate of HEK293 cells overexpressing Myc-tagged WFS1 (Zatyka et al., 2008) was used for IP with WFS1 antibody or corresponding IgG from non-immunized animals as a negative control, followed by in-gel trypsin digestion and protein identification via LC-MS/MS (Figures 4A and S5A). Apart from identifying known WFS1 interactors like sarco(endo)plasmic reticulum Ca^2+^ ATPase 2 (SERCA; also known as AT2A2) (Zatyka et al., 2015), several new interactors were identified such as voltage-dependent anion channel (VDAC) isoforms VDAC1, VDAC2, and VDAC3, and chaperone glucose-regulated proteins (GRP) GRP75 (Figure 4A). VDACs are known to regulate mitochondrial function by acting as gatekeepers of the transport of metabolites, nucleotides, and ions (Camara et al., 2017; Shoshan-Barmatz et al., 2010). We selected VDAC1 for further analysis because it is the most abundant protein on mitochondrial outer membrane (Camara et al., 2017), essential for neurite maintenance (Paschon et al., 2019), and had the highest MOWSE (molecular weight search) score among the VDAC isoforms in LC-MS/MS analysis (Figure 4A).

WFS1-VDAC1 interaction was confirmed in HEK293 cells by co-IP (Figures 4B and 4C). VDAC1 was detected only in Myc-WFS1 overexpressing cells immunoprecipitated with WFS1 antibody but not in negative controls with IgG from non-immunized animals (Figure 4C), and also not in cells transfected with empty-Myc vector immunoprecipitated with WFS1 antibody (Figure 4B). In empty-Myc expressing HEK293 cells, low level of endogenous WFS1 was not sufficient to detect VDAC1 by co-IP (Figure 4B). We further examined WFS1-VDAC1 interaction in WS5R hiPSC-derived NSCs where WFS1 protein could be restored by Dox treatment (Figures 3C and 4D). VDAC1 was detected by co-IP only in Dox-treated WS5R NSCs immunoprecipitated with WFS1 antibody but not in negative IgG control or in cells without Dox lacking WFS1 expression (Figure 4D). The immunoblots re-probed with respective antibodies demonstrated the amount of immunoprecipitated WFS1, and the inputs indicated the levels of WFS1 and VDAC1 in the samples (Figures 4B–4D). Furthermore, WFS1 colocalized with VDAC1 in WS5R NSCs treated with Dox, but no colocalization was seen in cells without Dox that did not express WFS1 (Figure 4E). These data suggest that WFS1 interacts and colocalizes with VDAC1, which is abolished upon loss of WFS1, thereby raising the possibility that this could impact the ability of VDAC1 to regulate mitochondrial function. However, VDAC1 protein levels and gene expression were not significantly different between CT and WS NSCs and neurons (Figures S5B–S5D), suggesting that mutations in WFS1 did not influence VDAC1 turnover and stability. Moreover, WFS1 exhibited some degree of colocalization with TOM20, which is an outer mitochondrial membrane protein, in Dox-treated WS5R NSCs (Figure S5E). Although we have not detected TOM20 as an interacting partner of WFS1 via mass spectrometry, this colocalization could be likely due to the proximity and involvement of WFS1 in MAMs.

### Restoration of WFS1 increases MAMs and improves mitochondrial dynamics

**Figure 5:**
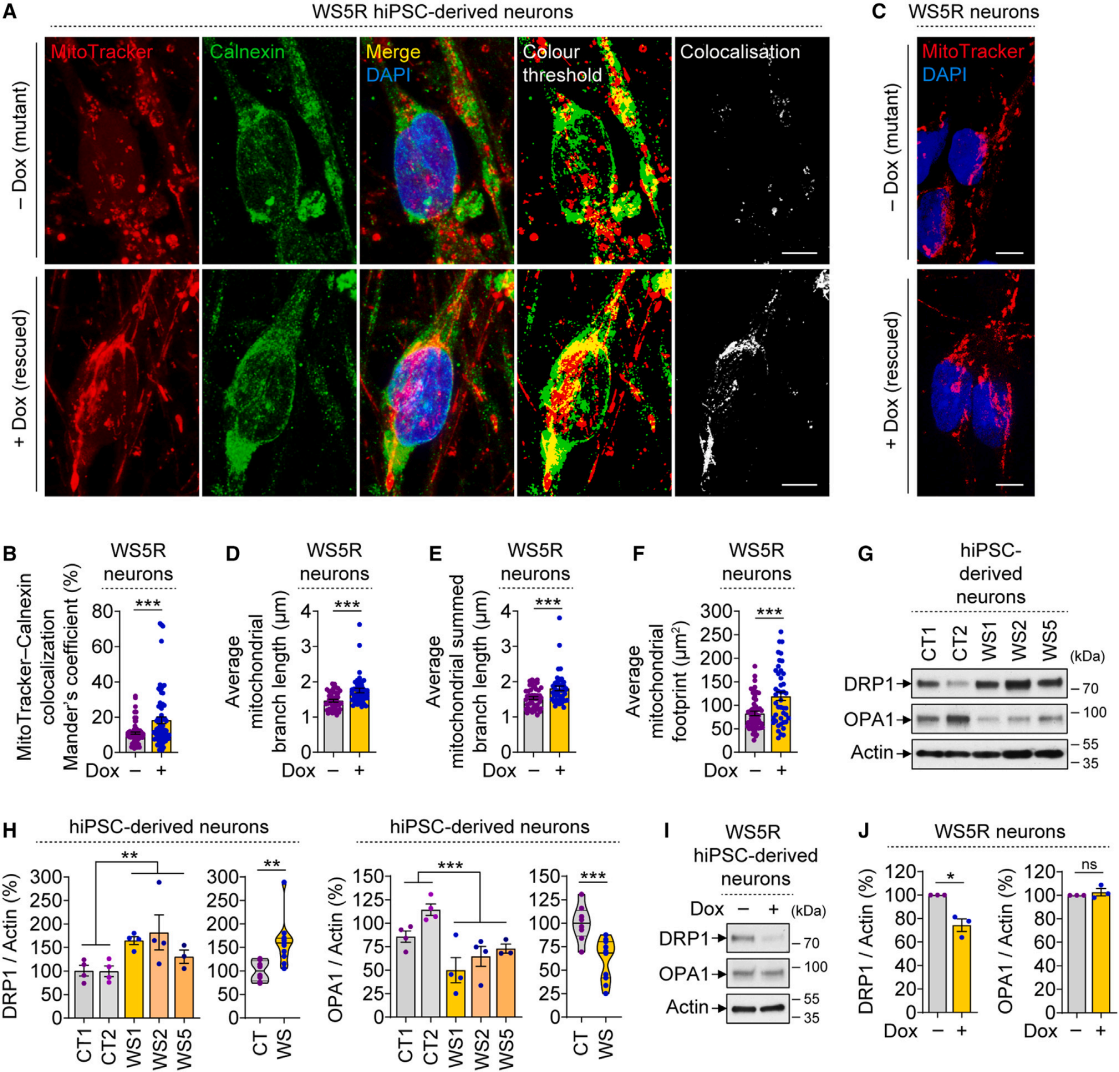
Increase in MAMs and mitochondrial branch length upon WFS1 restoration in WS patient hiPSC-derived cells exhibiting perturbed mitochondrial dynamics (A–F) Immunofluorescence images of MitoTracker and Calnexin (A), Mander’s coefficient of their colocalization (B), MitoTracker staining images (C), and average mitochondrial branch length (D), mitochondrial summed branch length (E), and mitochondrial footprint (F) in WS5R hiPSC-derived neurons (4 w), treated with or without 50 ng/mL Dox for 48 h. (G–J) Immunoblotting (G, I) and densitometric (H, J) analyses of DRP1 and OPA1 in hiPSC-derived neurons (4 w) of CT1, CT2, WS1, WS2, and WS5 (G, H) or of WS5R treated with or without 50 ng/mL Dox for 48 h (I, J). Graphical data are mean ± SEM of n = 3–4 biological replicates as indicated (H, J) or of ∼75 (B) or ∼50 (D–F) images per condition from n = 3 biological replicates or displayed as violin plots (line at median) of CT and WS groups (H). p values were calculated by unpaired two-tailed Student’s t test on three independent experiments (B, D–F, H, J). ^∗^p < 0.05; ^∗∗^p < 0.01; ^∗∗∗^p < 0.001; ns, non-significant. Scale bar, 5 μm (A, C).

Proper functioning of mitochondria relies on their spatial and temporal control in cells for which the mitochondria establish contact with different organelles, such as the ER to form MAMs (Delprat et al., 2018). Both WFS1 and VDAC1 are MAM-associated proteins (Delprat et al., 2018), and loss of WFS1 in WS reduced the number of MAMs in patient fibroblasts (Angebault et al., 2018). We thus studied if WFS1 restoration in WS patient-derived neurons could influence the MAMs, which was analyzed via the colocalization between MitoTracker (mitochondrial marker) and calnexin (ER marker) (Wang et al., 2021). Remarkably, restoration of WFS1 in Dox-treated WS5R neurons (rescued) significantly increased mitochondria-ER colocalization compared with WS5R neurons without Dox (mutant) (Figures 5A and 5B). This suggests a positive correlation between MAMs and WFS1 levels, thereby hinting at the possibility that reinstating WFS1-VDAC1 interaction in rescued cells might facilitate more MAM formation and consequently improve mitochondrial function.

Apart from the connection between mitochondria and ER, we also assessed mitochondrial network in our “rescued” system after MitoTracker staining (Valente et al., 2017). A moderate but significant increase in mitochondrial branch length, mitochondrial summed branch length, and mitochondrial footprint was observed upon WFS1 restoration in Dox-treated WS5R neurons, compared with WS5R neurons without Dox (Figures 5A and 5C–5F). We further analyzed some of the proteins involved in the core machinery of mitochondrial fission and fusion, which influence the dynamic nature of mitochondrial network, such as DRP1 (dynamin-related protein 1) and OPA1 (optic atrophy protein 1), respectively (Westermann, 2010). WS neurons exhibited increased DRP1 and decreased OPA1 levels compared with CT neurons (Figures 5G and 5H), raising the possibility that the mitochondria are probably undergoing more fission and less fusion in patient neurons that might contribute to shortening of the mitochondrial branch length. These data are in accordance with decreased mitochondrial length and fusion dynamics demonstrated in rat neuronal cells with *Wfs1* knockdown (Cagalinec et al., 2016). Genetic rescue by WFS1 restoration reduced DRP1 levels in Dox-treated WS5R neurons compared with WS5R neurons without Dox but had no effects on OPA1 (Figures 5I and 5J). This implies that reducing the fission events could improve mitochondrial branch length in WS neurons, although the fission and fusion processes are dynamic and reciprocally regulated (Sabouny and Shutt, 2020). Overall, our data suggest that restoring WFS1 levels in WS neurons increases MAMs and mitochondrial branch length.

### Pharmacological rescue of mitochondrial and cell death phenotypes

**Figure 6:**
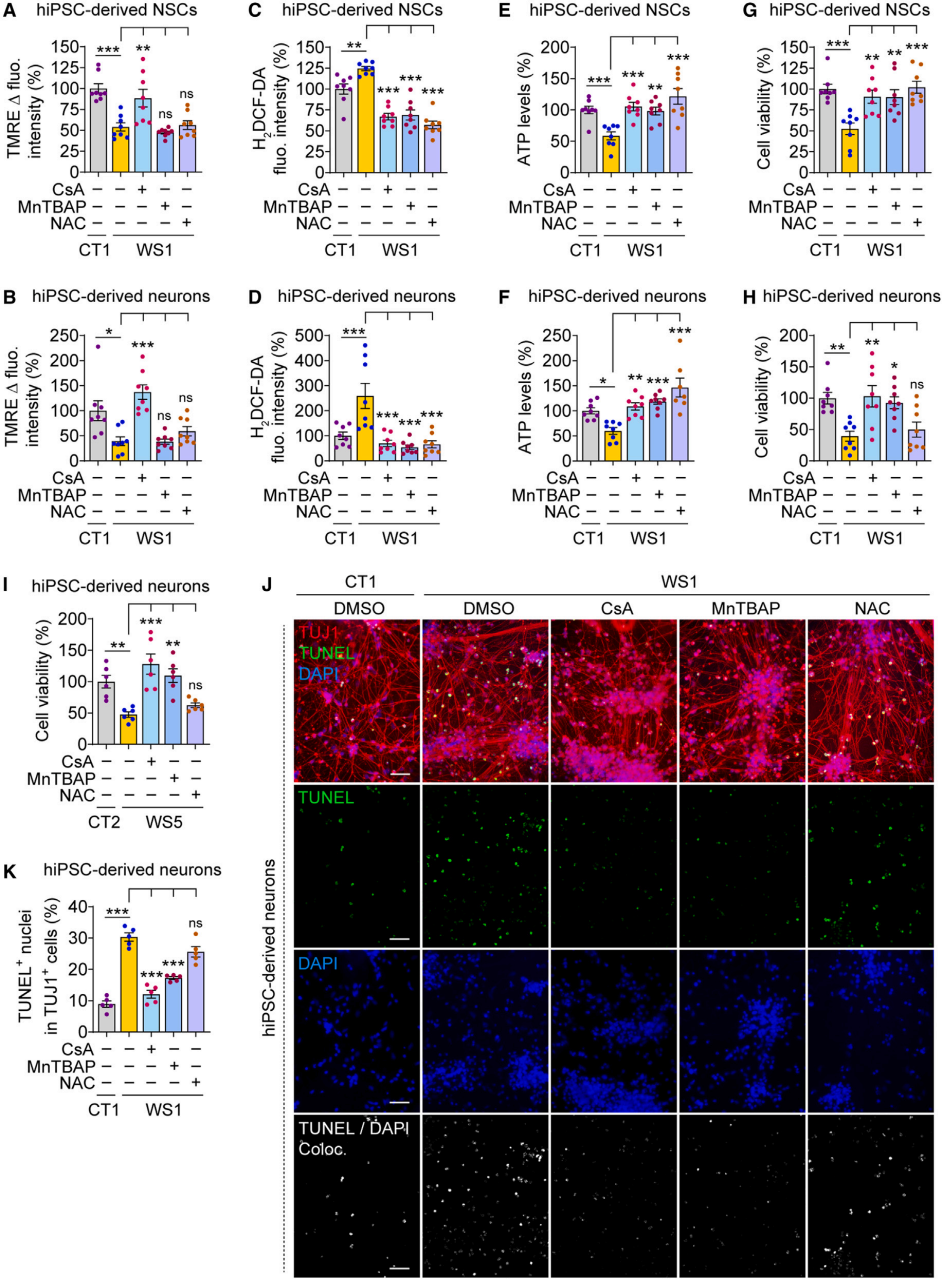
Pharmacological rescue of mitochondrial and cell death phenotypes in WS patient hiPSC-derived NSCs and neurons (A–J) Measurements of ΔΨm by TMRE Δ fluorescence intensity (A, B), ROS by H_2_DCF-DA fluorescence intensity (C, D), ATP levels (E, F) and cell viability (G–I), and immunofluorescence images of TUJ1 with TUNEL staining (J) and quantification of TUNEL^+^ apoptotic nuclei (K), in CT1 (A–H, J, K), CT2 (I), WS1 (A–H, J, K), and WS5 (I) hiPSC-derived NSCs (A, C, E, G) and neurons (4 w; B, D, F, H–K), where WS1 or WS5 cells were treated with or without 1 μM cyclosporin A (CsA), 2 μM MnTBAP, and 100 μM N-acetyl cysteine (NAC) for 2 (in NSCs; A, C, E, G) or 6 (in neurons; B, D, F, H–K) days, respectively. Graphical data are mean ± SEM of n = 5–8 biological replicates as indicated (A–I, K). p values were calculated by one-way ANOVA with Tukey’s multiple comparisons test on three independent experiments (A–I, K). ^∗^p < 0.05; ^∗∗^p < 0.01; ^∗∗∗^p < 0.001; ns, non-significant. Scale bar, 50 μm (J). See also Figure S5.

Finally, we utilized WS patient-derived neuronal platforms to evaluate the therapeutic efficacy of pharmacological agents modulating mitochondrial function. These compounds include (1) cyclosporin A (CsA), which inhibits mitochondrial permeability transition pore (PTP) and increases resting ΔΨ_m_ (Cassarino et al., 1998; Halestrap et al., 1997); (2) MnTBAP, which is a superoxide dismutase mimetic and superoxide scavenger (Faulkner et al., 1994); and (3) N-acetyl cysteine (NAC), which is an anti-oxidant and a free radical scavenger (Aldini et al., 2018) (Figure S5F). Since WS NSCs and neurons exhibited mitochondrial dysfunction, we examined the effects of these compounds on both cell types. Consistent with our findings (Figures 2A–2F), WS1 NSCs and neurons displayed reduction in ΔΨ_m_, elevation in ROS, and lower ATP levels compared with the CT1 counterparts (Figures 6A–6F). We found that CsA completely restored ΔΨ_m_ in WS1 cells owing to its mechanism of action of inhibiting the mitochondrial PTP, whereas MnTBAP and NAC did not have any effects (Figures 6A and 6B). However, these superoxide and free radical scavengers, as well as CsA, lowered ROS levels in WS1 cells (Figures 6C and 6D). Moreover, all the compounds elevated ATP levels in WS1 cells (Figures 6E and 6F). These data suggest that mitochondrial dysfunction in WS neuronal cells can be rescued by pharmacological interventions.

We next analyzed the effects of these compounds on the viability of WS neuronal cells. Both WS1 NSCs and neurons exhibited substantial reduction in cell survival compared with CT1 cells under basal state (Figures 6G and 6H). While all the compounds rescued cell viability in WS1 NSCs (Figure 6G), only CsA and MnTBAP were effective in WS1 neurons (Figure 6H). We further evaluated the effects of these compounds in neurons differentiated from another WS patient-derived line, WS5, which also had lower cell survival compared with CT2 neurons (Figure 6I). Similar to our findings in WS1 neurons, CsA and MnTBAP improved the viability of WS5 neurons (Figure 6I). We next assessed the cytoprotective effects of these compounds via terminal deoxynucleotidyl transferase dUTP nick end labeling (TUNEL) staining of apoptotic nuclei in neurons immunostained with the neuronal marker TUJ1. In line with decreased WS neuronal viability (Figures 3I, 6H, and 6I), TUJ1^+^ WS1 neurons had substantially higher amount of TUNEL^+^ staining compared with TUJ1^+^ CT1 neurons (Figures 6J and 6K), indicating higher basal cell death in WS1 neurons. CsA and MnTBAP decreased the number of TUNEL^+^ apoptotic nuclei in TUJ1^+^ WS1 neurons (Figures 6J and 6K). Although NAC had a tendency to lower cell death in WS neurons, its effect was not significant (Figures 6H–6K). We did not find any significant overcorrection effects of the drugs in rescuing ΔΨ_m_ and cell viability (Figures 6A, 6B, and 6G–6K), but ROS was suppressed in WS cells to levels lower than CT cells (Figures 6C and 6D). Collectively, these data indicate that modulating mitochondrial function by CsA or MnTBAP is cytoprotective in WS neuronal cells.

## Discussion

**Figure 7:**
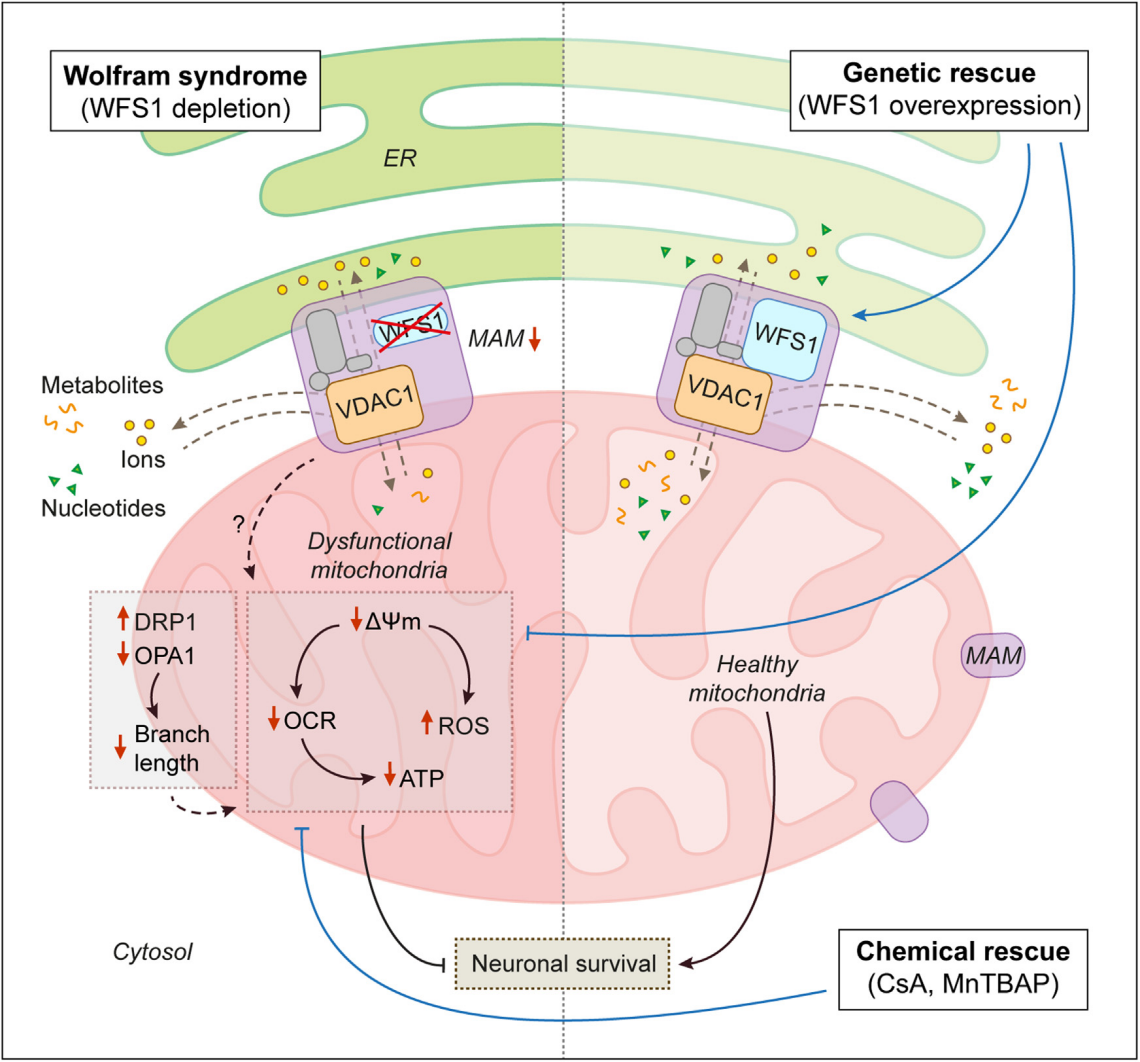
Schematic representation of genetic and chemical rescue of mitochondrial dysfunction in Wolfram syndrome Depletion of WFS1 in WS (left panel) causes mitochondrial dysfunction involving mitochondrial depolarization (lower ΔΨ_m_), oxidative stress (increased ROS), decreased mitochondrial respiration (less OCR), and lower ATP production, ultimately leading to neuronal cell death. Loss of WFS1-VDAC1 interaction in WS is suggested to decrease the MAMs and their function that could possibly deregulate mitochondrial dynamics (increased DRP1, decreased OPA1, and shortening of branch length) and also affect the metabolic functions of mitochondria by perturbing the transport of ions, nucleotides, and metabolites. Genetic rescue by WFS1 overexpression or chemical rescue by CsA and MnTBAP (right panel) attenuates the mitochondrial phenotypes and improves neuronal survival. WFS1 restoration in WS reinstates WFS1-VDAC1 interaction and increases MAMs and mitochondrial network that are suggested to improve mitochondrial function.

In summary, we show a causal link between loss of WFS1 and mitochondrial dysfunction that could be rescued by genetic or pharmacological interventions (Figure 7). RNA-seq analysis revealed significant perturbations in mitochondria-associated genes in WS patient hiPSC-derived NSCs and neurons, which exhibited mitochondrial depolarization, oxidative stress, and reduction in mitochondrial respiration and ATP production. The mitochondrial and cell death phenotypes appear to be more aggravated in WS neurons than WS NSCs. Since the mitochondrial abnormalities were detrimental for WS patient-derived neuronal cells, our data are suggestive of mitochondrial dysfunction contributing to neurodegeneration (Johri and Beal, 2012; Nunnari and Suomalainen, 2012). While there are contradictory reports on mitochondrial functionality in cell models that are either non-human or not disease relevant, our data in the patient-derived disease-affected neuronal platforms are consistent with some of the phenotypes described in previous studies. These include a reduction in ΔΨ_m_ in rat cortical neurons with siRNA-mediated *Wfs1* knockdown (Cagalinec et al., 2016), lower mitochondrial respiration in WS patient fibroblasts (Angebault et al., 2018), and decreased ATP level in mouse β-cell-derived MIN6 cell line with shRNA-mediated *Wfs1* knockdown (Zatyka et al., 2015). It is plausible to speculate that loss of WFS1 could indirectly affect the mRNA levels of mitochondria-associated genes via the unfolded protein response (UPR) pathway, which is an adaptive response to combat ER stress underlying WS (Fonseca et al., 2010). The UPR effectors include a range of transcription factors such as ATF4, ATF5, ATF6, and spliced XBP1, which can regulate the transcription of various proteins implicated in mitochondrial function, biogenesis, and turnover (Senft and Ronai, 2015).

Genetic rescue by restoration of WFS1 levels abrogated the mitochondrial phenotypes and improved neuronal viability (Figure 7), implying a potential role of WSF1 in the maintenance of mitochondrial function that is attributable to neuronal survival. One possible mechanism could be via its interaction with VDAC1, which we identified as a WFS1 interactor by mass spectrometry. VDAC1 forms a complex with IP_3_R on the ER through the molecular chaperone GRP75 (Szabadkai et al., 2006), and the WFS1-VDAC1 interaction is likely a part of this complex tethering ER and mitochondria because both these proteins are associated with MAMs (Delprat et al., 2018). Restoring WFS1 levels in WS cells reinstated WFS1-VDAC1 interaction and increased MAMs. We thus speculate that this interaction could facilitate the close contact between ER and mitochondrial outer membrane required for MAM formation and function. Concomitantly, restoration of WFS1 in WS cells increased mitochondrial branch length that correlated with reduction in the mitochondrial fission protein DRP1. Taken together, we further speculate that improved mitochondrial dynamics coupled with more MAMs could positively impact on mitochondrial function. Conversely, impairment in MAMs and mitochondrial dynamics are implicated in neurodegenerative diseases (Delprat et al., 2018; Westermann, 2010), and their dysfunction demonstrated in WS is possibly disrupting mitochondrial function (Angebault et al., 2018; Cagalinec et al., 2016).

Moreover, VDAC1 is a multifunctional channel of mitochondrial outer membrane that is essential for the metabolic functions of mitochondria by controlling the transport of ions, nucleotides, and metabolites (Camara et al., 2017; Shoshan-Barmatz et al., 2010). The IP_3_R-GRP75-VDAC1 complex on the ER allows Ca^2+^ transfer from the ER to mitochondria (Szabadkai et al., 2006). VDAC1 also mediates metabolic flow in the opposite direction, creating ATP microdomain close to the ER and SERCA (Camara et al., 2017; Shoshan-Barmatz et al., 2010). Neurodegeneration-associated proteins, such as DJ-1 in early-onset PD and WFS1 in WS, interact with the IP_3_R-GRP75-VDAC1 multicomplex to influence mitochondrial and MAM functions (Angebault et al., 2018; Liu et al., 2019). In the context of WS, WFS1 also forms a complex with IP_3_R and NCS1 to promote Ca^2+^ transfer from ER to mitochondria (Angebault et al., 2018). Identification of VDAC1 as a WFS1 interactor strengthens the finding that WFS1 is a part of the IP_3_R-GRP75-VDAC1 multicomplex, and interestingly, we also identified VDAC2, VDAC3, and GRP75 among the potential WFS1 interactors via mass spectrometry. Furthermore, it has been hypothesized that WFS1 could control the oligomerization of Na^+^ pump subunits (Zatyka et al., 2008), and hence it might also govern VDAC1 oligomerization state and channel function. From these findings, it is plausible that loss of WFS1-VDAC1 interaction observed in WS patient-derived cells lacking WFS1 could be a potential mechanism via which mutant WFS1 might affect VDAC1 and mitochondrial functionality (Figure 7). However, we did not observe any overt perturbations in mitochondrial Ca^2+^ in WS patient-derived neuronal cells, which also displayed variability among the WS cell lines. Further work is warranted in disease-relevant cellular platforms to elucidate how mutant WFS1 could disrupt VDAC1 function or how loss of WFS1-VDAC1 interaction leads to mitochondrial dysfunction.

Of biomedical relevance, pharmacological modulation of mitochondrial function via inhibition of mitochondrial PTP by CsA or suppression of superoxide by MnTBAP, which respectively restored ΔΨ_m_ and lowered oxidative stress, recovered the energy status and viability of WS NSCs and neurons (Figure 7). However, the effects of the anti-oxidant NAC on neuronal viability were not significant, which could be related to its side effects like autophagy inhibition that can augment neurodegeneration (Underwood et al., 2010). Previous studies have shown CsA to be protective in AD cell models and in rat heart after ischemia/reperfusion injury by restoring ΔΨ_m_ (Cassarino et al., 1998; Halestrap et al., 1997), while MnTBAP was protective against renal injury and obesity-induced cardiac dysfunction by antagonizing oxidative stress (Bi et al., 2018; Ilkun et al., 2015; Yu et al., 2016). We found that these pharmacological agents were also cytoprotective in the context of WS patient-derived neuronal cells. Our data highlight a potential therapeutic intervention for WS that could be further examined for generalizability in related rare or common neurodegenerative diseases associated with mitochondrial defects.

## Experimental procedures

Detailed methodologies can be found in supplemental experimental procedures in the supplemental information.

### Resource availability

#### Corresponding author

Sovan Sarkar (s.sarkar@bham.ac.uk).

#### Materials availability

The hiPSC-derived NSCs were generated by L. Aubry (I-STEM, France) and obtained by S.S. and T.B. under a materials transfer agreement (MTA 1390060). The WIBR3 hESC line was obtained by S.S. from R. Jaenisch (Whitehead Institute for Biomedical Research, USA) under a materials transfer agreement (UBMTA 15–0593).

### hiPSC lines and neuronal differentiation

The NSCs and neurons were generated from previously established control (CT1, CT2), WS patient-derived (WS1, WS2, WS5), and rescued (WS5R) hiPSC lines (Pourtoy-Brasselet et al., 2021; Shang et al., 2014). The hiPSCs were cultured and differentiated into NSCs and then into neurons as described previously (Boissart et al., 2013; Pourtoy-Brasselet et al., 2021). Neuronal differentiation was done for 4 weeks; neurons were cortical in nature.

### RNA-seq data and gene expression analysis

AmpliSeq data (GEO: GSE156911) used in this study were previously published (Pourtoy-Brasselet et al., 2021). Commonly expressed upregulated and downregulated DEGs (p value ≤ 5%; fold change ≥ 1.5; minimum reads > 100) between mitochondria gene set of control and WS NSCs or neurons were selected using Venny diagram (v2.1.0). Mitochondria-associated DEGs were depicted in a volcano plot by plotting the magnitude of change [Log_2_(Fold change)] against the measure of significance [Log_10_(*P* adjusted)]. Gene expression was analyzed by quantitative real-time PCR (qPCR) using gene-specific primers, qPCR data calculated by 2^−ΔΔCt^ method, normalized to *GAPDH* expression, and calculated as percentage of control condition.

### Identification of WFS1 interactors by mass spectrometry

HEK293 cell lysate overexpressing Myc-WFS1 was used for IP with WFS1 antibody or IgG from non-immunized animals using Dynabeads Protein A Immunoprecipitation Kit (Invitrogen). Co-IP proteins were separated by SDS-PAGE followed by in-gel tryptic digestion. The resulting peptides were analyzed by LC-MS/MS using Bruker Impact Q-ToF Mass Spectrometer (Bruker Daltonics). Peptides were identified using MASCOT to search the SWISSPROT human database, and protein identifications were filtered using a 1% false discovery rate and a requirement for ≥2 peptides using ProteinScape software (Bruker Daltonics). Mass spectrometry data for WFS1 interactors have been deposited in MassIVE repository (MassIVE: MSV000091646).

### Mitochondrial ΔΨ_m_, ROS, Ca^2+^, and ATP measurements

Measurements of ΔΨ_m_, ROS, mitochondrial Ca^2+^, and ATP were respectively done using 500 nM TMRE, 20 μM CM-H_2_DCF-DA, 10 μM Fluo-3 AM (Invitrogen), and ApoSENSOR ADP/ATP Ratio Bioluminescent Assay Kit (BioVision) as described previously (Rosenstock et al., 2022) or per manufacturer’s protocol. Data were normalized to protein concentration by Bradford Protein Assay (Bio-Rad) and expressed as percentage of control condition.

### Mitochondrial respiration measurement

Basal levels of OCRs were measured on XFe96 Extracellular Flux Analyzer (Agilent) after stimulation with 2 μM oligomycin, 3 μM BAM15, and 1 μM rotenone/antimycin A (Sigma-Aldrich) per XF Cell Mito Stress Test Kit (Agilent). Basal respiration, ATP production, proton leak, and maximal respiration were calculated, and cell number was normalized by CyQUANT Direct Cell Proliferation Assay (Invitrogen).

### Measurements of MAMs and mitochondrial branch length

Analyses of MAMs (Wang et al., 2021) was done by Mander’s coefficient of colocalization between MitoTracker Red CMXRos (Invitrogen) and Calnexin (∼75 images per sample). Measurements of mitochondrial branch length and footprint (Valente et al., 2017) were done using Mitochondrial Network Analysis (MiNA) toolset in Fiji v2.9.0 (ImageJ2) after MitoTracker Red CMXRos staining (∼50 images per sample).

### Measurements of cell viability and apoptotic cells

Cell viability was measured by luminescence-based CytoTox-Glo Cytotoxicity Assay (Promega) per manufacturer’s protocol. Data were expressed as percentage of control condition. Apoptotic cells were determined by Click-iT Plus TUNEL Assay for *in situ* apoptosis detection, Alexa Fluor 488 dye (Thermo Fisher Scientific) followed by immunostaining with TUJ1 antibody. The percentage of TUNEL^+^ apoptotic nuclei was calculated from all the TUJ1^+^ cells analyzed (200–300 cells per sample).

### Statistical analysis

Graphical data are from ≥3 biological replicates from independent experiments and depicted as column graph scatter dot plot (mean ± SEM) or violin plot (line at median) using Prism v8.3.1 (GraphPad). Statistical significance (p value) was determined by unpaired two-tailed Student’s t test or by one-way ANOVA with Tukey’s or Dunnett’s multiple comparisons test using Prism v8.3.1 (GraphPad). ^∗^p < 0.05; ^∗∗^p < 0.01; ^∗∗∗^p < 0.001; ns: non-significant.

## Author contributions

M.Z., T.R.R., C.S., A.M.P., G.W.H., S.L.R., D.A., A.D.M., A.S., M.E.K., V.S., G.Z., M.R., S.P.B., K.W., T.V., M.J., H.P., J.C., M.H., D.G.W., L.A., and S.S. performed experiments, provided tools/methodologies, and/or analyzed data; S.S., T.B., T.R.R., L.A., M.E.K., and E.M.F. acquired funding; S.S., T.R.R., and M.Z. conceptualized the project; S.S. administered the project, prepared figures, and wrote the manuscript with inputs from M.Z., T.R.R., L.A., and T.B.; all authors contributed to and/or approved the final manuscript.

## Data Availability

The GEO accession number of the RNA-seq data is GSE156911. Mass spectrometry data for WFS1 interactors are available in MassIVE repository (MassIVE: MSV000091646). Data of the results are presented in the main paper and the supplemental information. This paper does not report any original code.

## Supplemental information

Document S1. Supplemental experimental procedures, Figures S1–S5, and Tables S1–S7

Document S2. Article plus supplemental information
