# Supplementary material for: Depletion of WFS1 compromises mitochondrial function in hiPSC-derived neuronal models of Wolfram syndrome
Source: Stem Cell Reports. 2023 May 9;18(5):1090–106. doi: 10.1016/j.stemcr.2023.04.002 (PMC10202695; doi:10.1016/j.stemcr.2023.04.002)
Supplement: Document S1. Supplemental experimental procedures, Figures S1–S5, and Tables S1–S7 [file mmc1.pdf]

Stem Cell Reports, Volume *18*

Supplemental Information

Depletion of WFS1 compromises mitochondrial function in hiPSC-derived neuronal models of Wolfram syndrome

Malgorzata Zatyka, Tatiana R. Rosenstock, Congxin Sun, Adina M. Palhegyi, Georgina W. Hughes, Samuel Lara-Reyna, Dewi Astuti, Alessandro di Maio, Axel Sciauvaud, Miriam E. Korsgen, Vesna Stanulovic, Gamze Kocak, Malgorzata Rak, Sandra Pourtoy-Brasselet, Katherine Winter, Thiago Varga, Margot Jarrige, Hélène Polvèche, Joao Correia, Eva-Maria Frickel, Maarten Hoogenkamp, Douglas G. Ward, Laetitia Aubry, Timothy Barrett, and Sovan Sarkar

SUPPLEMENTAL FIGURES

Figure S1

A hiPSC lines from which neural stem cells and neurons were generated

hiPSC line	Genotype	Mutations in *WFS1* gene
CT1	Healthy control	wild-type
CT2	Healthy control	wild-type
WS1	WS patient	c.1230-1233delCTCT [p.V412fs*440]; c.2171C>T [p.P724L]
WS2	WS patient	c.1230-1233delCTCT [p.V412fs*440]; c.1230-1233delCTCT [p.V412fs*440]
WS5	WS patient	c.1060-1062delTTC [p.F354del]; c.1676C>A [p.A559D]
WS5R	WS patient 'rescued'	WS5 corrected control after Dox treatment; *WFS1* cDNA under Dox promoter inserted at *AAVS1* locus

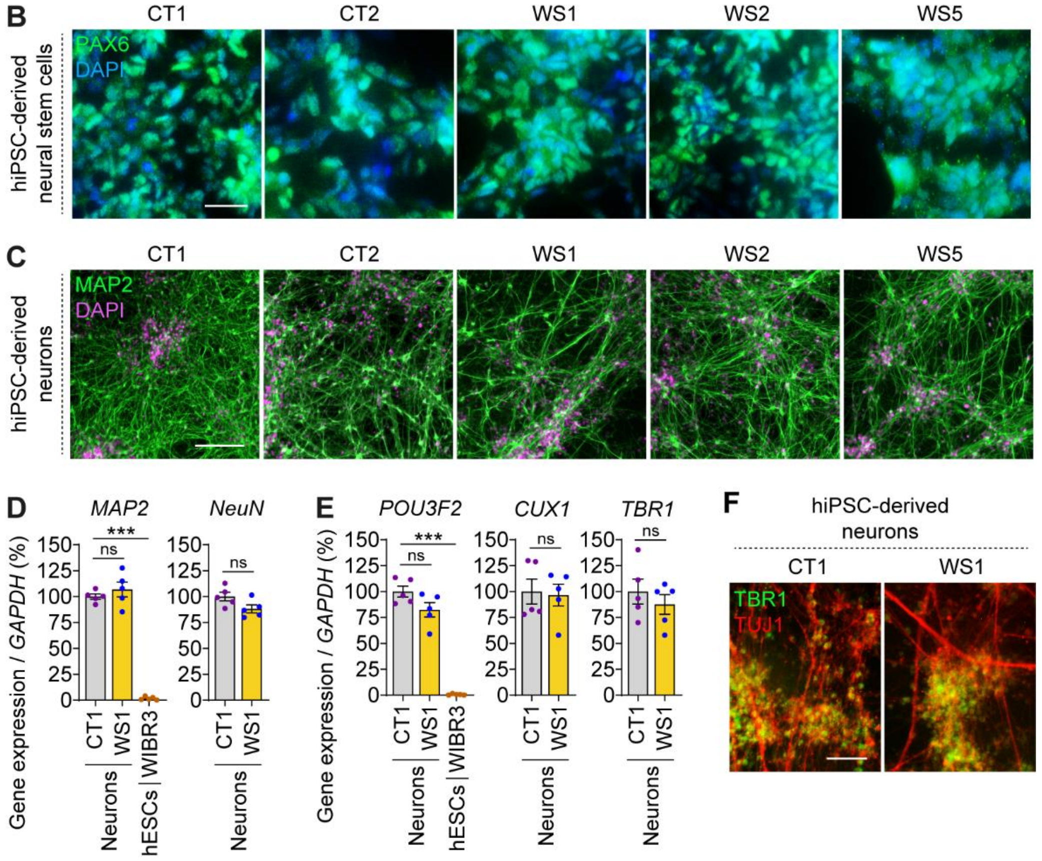

Figure S1. Characterization of hiPSC-derived neural stem cells and neurons; related to Figure 1.
(A) List of control (CT1 and CT2) and Wolfram syndrome (WS1, WS2 and WS5) patient-derived hiPSC lines from which neural stem cells (NSCs) and neurons were generated in this study.
(B, C) Immunofluorescence images of PAX6 (B) and MAP2 (C) in CT1, CT2, WS1, WS2 and WS5 hiPSC-derived NSCs (B) and 4 weeks (4 w) old neurons (C).
(D, E) qPCR expression analyses of *MAP2* (D), *NeuN* (D), *POU3F2* (E), *CUX1* (E) and *TBR1* (E) in CT1 and WS1 hiPSC-derived neurons (4 w); WIBR3 hESCs were used as a negative control (D, E).
(F) Immunofluorescence images of TBR1 and TUJ1 in CT1 and WS1 hiPSC-derived neurons (4 w).
Graphical data are mean ± s.e.m. of n = 5 biological replicates (D, E). P values were calculated by unpaired two-tailed Student's t-test (D, E; right and middle panels) or one-way ANOVA with Dunnett's multiple comparisons test (D, E; left panels) on 3 independent experiments. ***P<0.001; ns, non-significant. Scale bar, 25 µm (B), 50 µm (F), 100 µm (C).

Figure S2

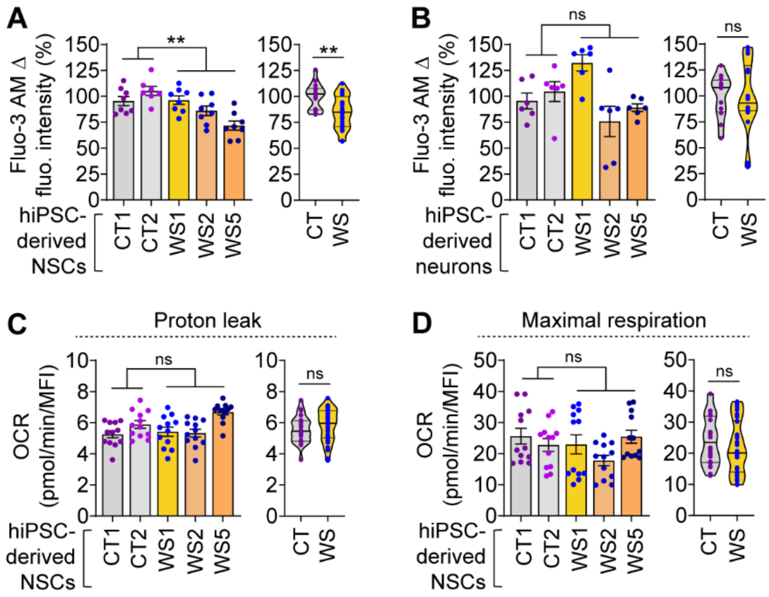

Figure S2. Analysis of mitochondrial Ca^{2+} and respiratory parameters in WS patient hiPSC-derived cells; related to Figure 2.

(A, B) Measurement of mitochondrial Ca^{2+} by Fluo-3 AM Δ fluorescence intensity in CT1, CT2, WS1, WS2 and WS5 hiPSC-derived NSCs (A) and neurons (4 w) (B).

(C, D) Oxygen consumption rate (OCR) levels were measured post mitochondrial stress test, involving oligomycin (Oligo), BAM15 and rotenone (Rot)/antimycin A (AA) treatment, in CT1, CT2, WS1, WS2 and WS5 hiPSC-derived NSCs, wherein proton leak (C) and maximal respiration (D) were calculated as described in experimental procedures and Table S6. MFI: mean fluorescent intensity. Graphical data are mean ± s.e.m. of n = 6–12 biological replicates as indicated (A–D), or displayed as violin plots (line at median) of CT and WS groups (A–D). P values were calculated by unpaired two-tailed Student's t-test on 2 (A, B) or 3 (C, D) independent experiments. **P<0.01; ns, non-significant.

Figure S3

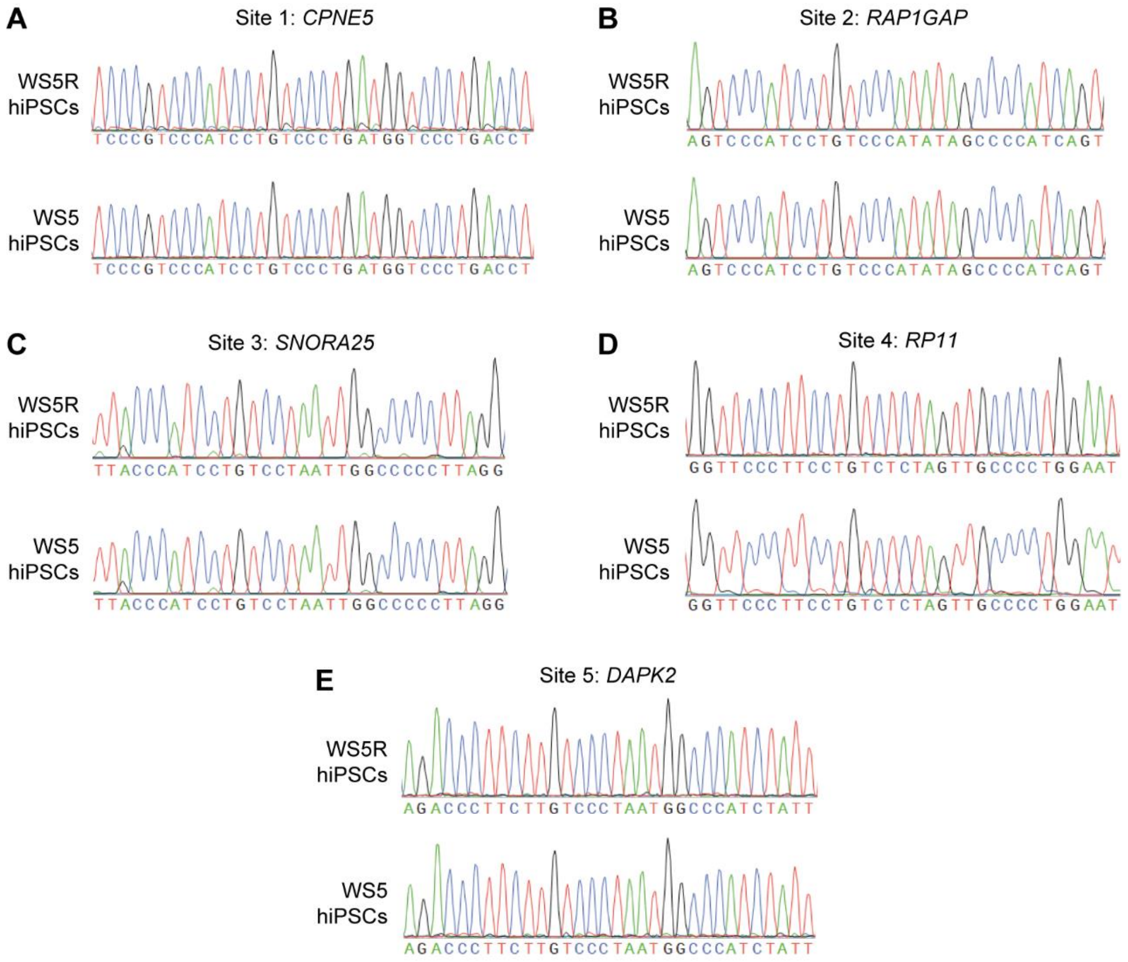

Figure S3. Off-target analysis in genome-edited WS5R hiPSC line; related to Figure 3.
(A–E) Potential off-target loci sequencing of top 5 sites, as determined by CRISPOR, illustrating no differences between WS5 and WS5R hiPSCs. Site 1: *CPNE5* (A); site 2: *RAP1GAP* (B); site 3: *SNORA25* (C); site 4: *RP11* (D); and site 5: *DAPK2* (E).

Figure S4

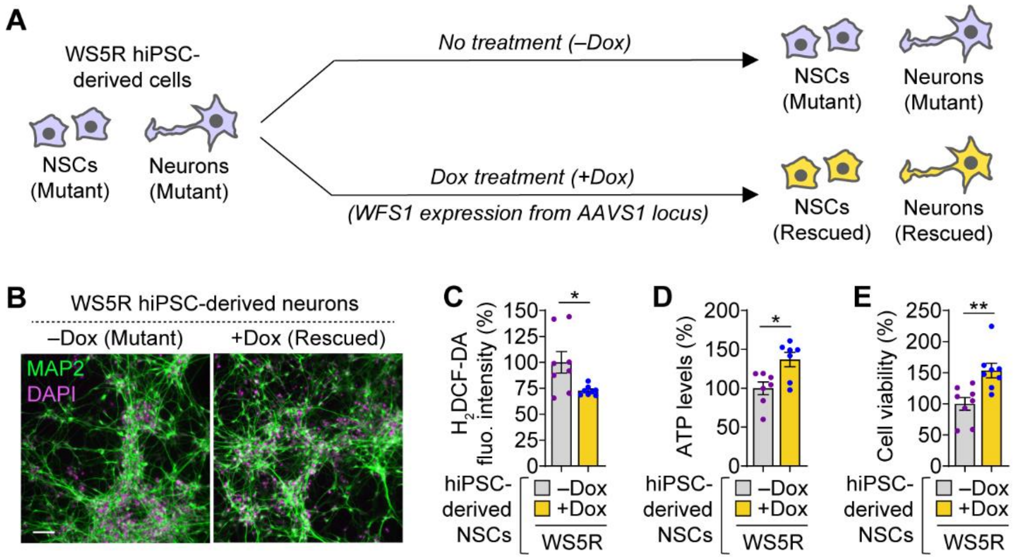

Figure S4. Genetic rescue of mitochondrial and cell death phenotypes in genome-edited WS patient hiPSC-derived cells; related to Figure 3.

(A) Genetic rescue in genome-edited WS5R hiPSC-derived neural stem cells (NSCs) and neurons, wherein WS5R cells treated with doxycycline (Dox) act as 'rescued' cells due to *WFS1* expression from *AAVS1* locus but WS5R cells in the absence of Dox remain as mutant cells.

(B) Immunofluorescence images of MAP2 in WS5R hiPSC-derived neurons (4 w), treated with or without 50 ng/mL Dox for 48 h.

(C–E) Measurements of ROS by H_2DCF-DA fluorescence intensity (C), ATP levels (D) and cell viability (E) in WS5R hiPSC-derived NSCs, treated with or without 50 ng/mL Dox for 48 h.

Graphical data are mean ± s.e.m. of n = 7–8 biological replicates as indicated (C–E). P values were calculated by unpaired two-tailed Student's t-test on 3 independent experiments (C–E). *P<0.05; **P<0.01. Scale bar, 100 µm (B).

Figure S5

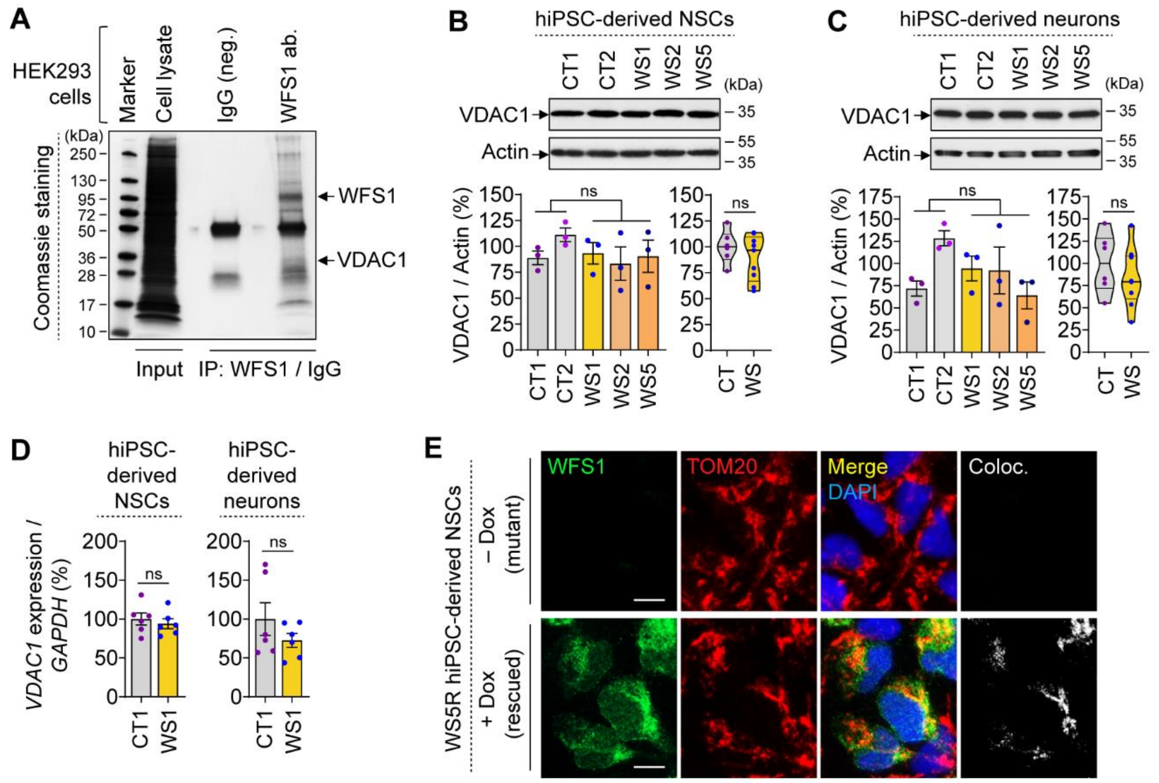

F Compounds used for modulating mitochondrial phenotype

Compounds	Cell type used	Property
Cyslosporin A (CsA)	hiPSC-derived NSCs & neurons	Inhibits mitochondrial permeability transition pore
MnTBAP	hiPSC-derived NSCs & neurons	Superoxide dismutase mimetic and superoxide scavenger
N-acetyl cysteine (NAC)	hiPSC-derived NSCs & neurons	Antioxidant and free radical scavenger

Figure S5. VDAC1 levels and WFS1–TOM20 colocalization in WS patient hiPSC-derived cells; related to Figure 4 and 6.

(A) Coomassie staining of SDS-PAGE gel with cell lysate (input) or after immunoprecipitation with WFS1 antibody or IgG (negative control) in HEK293 cells expressing Myc-WFS1. Approximate bands of WFS1 and VDAC1 are shown after pulldown.

(B, C) Immunoblotting and densitometric analyses of VDAC1 relative to Actin in CT1, CT2, WS1, WS2 and WS5 hiPSC-derived NSCs (B) and neurons (4 w) (C).

(D) qPCR expression analysis of *VDAC1* relative to *GAPDH* in CT1 and WS1 hiPSC-derived NSCs and neurons (4 w).

(E) Immunofluorescence images of TOM20 and WFS1 colocalization in WS5R hiPSC-derived NSCs, treated with or without 50 ng/ml Dox for 48 h.

(F) Details of compounds used for modulating mitochondrial phenotypes in WS hiPSC-derived NSCs and neurons.

Graphical data are mean ± s.e.m. of n = 3–6 biological replicates as indicated (B–D) or displayed as violin plots (line at median) of CT and WS groups (B, C). P values were calculated by unpaired two-tailed Student's t-test on 3 independent experiments (B–D). ns, non-significant. Scale bar, 10 μm (E).

SUPPLEMENTAL TABLES

Table S1

Compound name	Solvent	Stock conc.	Final conc.	Catalogue number	Source
Cyclosporin A (CsA)	DMSO	1 mg/mL	1 µM	SML1018	Merck
Mn(III)tetrakis(4-benzoic acid)porphyrin Chloride (MnTBAP)	NaOH, 80%	2 mM	2 µM	475870	Merck
N-Acetyl-L-cysteine (NAC)	H_2O	100 mM	100 µM	A9165	Merck

Table S1. List of compounds used for modulating mitochondrial function; related to Supplemental Experimental Procedures on compound treatment.
Details of compounds used in this study for modulating mitochondrial function in hiPSC-derived neural stem cells and neurons.

Table S2

Gene	Species	Primer direction	Primer sequence	Source	Reference
ATP5PD	Human	Forward	GCT GCT TTA CCT GAG AAT CCA CC	Merck	Origene (HP230531)
		Reverse	TAT CCT CTG GCA CGG GAA CCT T		
COX16	Human	Forward	TGG AGG TTC TTT TGG TCT TCG TG	Merck	Origene (HP212207)
		Reverse	TCT CAT ATT CCG ACT CTA AAG ATA TT		
CUX1	Human	Forward	TCC GTA GCA TCC AAG GCA GAC A	Merck	Origene (HP233477)
		Reverse	CTT CAT CAG AGC CAG TCT CCG A		
GAPDH	Human	Forward	GTC TCC TCT GAC TTC AAC AGC G	Merck	This study
		Reverse	ACC ACC CTG TTG CTG TAG CCA A		
MAP2	Human	Forward	AGG CCC AAG CTA AAG TTG GT	Merck	This study
		Reverse	ATG GTC CAC ACG GGC TTT AG		
NDUFA10	Human	Forward	TGG CTC AAG CAG GAC AAT CGC A	Merck	Origene (HP207839)
		Reverse	AGA CAC GGT CAG TCT GAT GAG C		
NeuN	Human	Forward	TAC AGC GAC AGT TAC GGC AG	Merck	This study
		Reverse	TTC CAA TGC TGT AGG TCG CC		
POU3F2	Human	Forward	GTG TTC TCG CAG ACC ACC ATC T	Merck	Origene (HP208713)
		Reverse	GCT GCG ATC TTG TCT ATG CTC G		
SDHB	Human	Forward	GCA GTC CAT AGA AGA GCG TGA G	Merck	Origene (HP206603)
		Reverse	TGT CTC CGT TCC ACC AGT AGC T		
TBR1	Human	Forward	TCA CTG GAG GTT TCA AGG AGG C	Merck	Origene (HP209514)
		Reverse	TTT CTT GGC GCA TCC AGT GAG C		
VDAC1	Human	Forward	GCA AAA TCC CGA GTG ACC CAG A	Merck	Origene (HP206903)
		Reverse	TCC AGG CAA GAT TGA CAG CGG T		

Table S2. List of primers for gene expression analysis; related to Supplemental Experimental Procedures on gene expression analysis.
Details of primers used in this study for gene expression analysis in hiPSC-derived neural stem cells and neurons.

Table S3

Antigen	Host species	Source	Catalogue number	Dilution
**Primary antibodies for immunoblotting**				
Actin	Mouse	Merck	A5441	1:2000
DRP1	Rabbit	Cell Signaling Technology	8570S	1:1000
c-Myc	Mouse	Merck	A5546	1:1000
OPA1	Rabbit	Cell Signaling Technology	67589S	1:1000
VDAC1	Mouse	Abcam	ab186321	1:1000
WFS1	Rabbit	Cell Signaling Technology	8749S	1:1000
WFS1	Sheep	R&D Systems	AF7417	1:1000
**Secondary antibodies for immunoblotting**				
Anti-mouse IgG, HRP-conjugated	Rabbit	Dako	P0161	1:10000
Anti-rabbit IgG, HRP-conjugated	Goat	Dako	P0448	1:10000
Anti-sheep IgG, HRP-conjugated	Donkey	R&D Systems	HAF016	1:2000
Anti-mouse IgG light chain, HRP-conjugated	Goat	Jackson Immuno Research	115-035-174	1:10000

Table S3. List of primary and secondary antibodies for immunoblotting; related to Supplemental Experimental Procedures on immunoblotting analysis.
Details of antibodies used in this study for immunoblotting analysis in hiPSC-derived neural stem cells and neurons and in HEK293 cells.

Table S4

Antigen	Host species	Source	Catalogue number	Dilution
**Primary antibodies for immunofluorescence**				
Calnexin	Rabbit	Abcam	ab22595	1:500
MAP2	Rabbit	Cell Signaling Technology	8707S	1:200
NESTIN	Rabbit	BioLegend	839801	1:500
PAX6	Rabbit	BioLegend	901301	1:100
TBR1	Rabbit	Cell Signaling Technology	49661S	1:250
TOM20	Mouse	Santa Cruz Biotechnology	sc17764	1:100
β3-Tubulin (TUJ1)	Mouse	Cell Signaling Technology	4466S	1:200
VDAC1	Mouse	Abcam	ab186321	1:100
WFS1	Rabbit	Cell Signaling Technology	8749S	1:80
**Secondary antibodies for immunofluorescence**				
Anti-mouse IgG (H+L), Alexa Fluor 594	Donkey	Thermo Fisher Scientific	A-21203	1:500
Anti-rabbit IgG (H+L), Alexa Fluor 488	Donkey	Thermo Fisher Scientific	A-21206	1:1000

Table S4. List of primary and secondary antibodies for immunofluorescence; related to Supplemental Experimental Procedures on immunofluorescence analysis.
Details of antibodies used in this study for immunofluorescence analysis in hiPSC-derived neural stem cells and neurons.

Table S5

Antigen	Host species	Source	Catalogue number	Concentration
**Primary antibodies for immunoprecipitation**				
WFS1	Rabbit	Cell Signaling Technology	8749S	0.007 µg/µL
Normal IgG	Rabbit	Cell Signaling Technology	2729S	0.007 µg/µL
Normal IgG	Rabbit	R&D Systems	AB105-c	0.007 µg/µL

Table S5. List of primary antibodies for immunoprecipitation; related to Supplemental Experimental Procedures on co-immunoprecipitation.
Details of antibodies used in this study for immunoprecipitation in hiPSC-derived neural stem cells and in HEK293 cells.

Table S6

Parameter	Equation
Basal respiration	(measurement before oligomycin stimulation) – (rate measurement after rotenone/antimycin A stimulation)
ATP production	(basal respiration) – (minimum rate measurement after oligomycin stimulation)
Proton leak	(minimum rate measurement after oligomycin stimulation) – (minimum measurement after rotenone/antimycin A stimulation)
Maximal respiration	(maximum rate measurement after BAM15 stimulation) – (minimum rate measurement after rotenone/antimycin A stimulation)

Table S6. Calculations for metabolic parameters in mitochondrial respiration measurements; related to Supplemental Experimental Procedures on mitochondrial respiration measurement. Details of calculations used in this study for assessing basal respiration, ATP production, proton leak and maximal respiration in hiPSC-derived neural stem cells and neurons.

Table S7

Gene	Species	Primer direction	Primer sequence	Source	Reference
CPNE5	Human	Forward	TGGACACCAAAGAATGGTGA	Eurofins	CRISPOR
		Reverse	CTGGCATCTGCAGGGTTATT		
RAP1GAP	Human	Forward	ATACCCACGCCCCTATACTC	Eurofins	CRISPOR
		Reverse	CTAGTGACAGCTGTGTGGTC		
SNORA25	Human	Forward	CGCGCCTCCCTTTATAAAGT	Eurofins	CRISPOR
		Reverse	ATGGTAGGCTCAGACACAGA		
RP11	Human	Forward	CCGGGAAATGTTATGAATGG	Eurofins	CRISPOR
		Reverse	TCACATGGAAGCACATGGAT		
DAPK2	Human	Forward	TGTGGAAATGGGGATGACTT	Eurofins	CRISPOR
		Reverse	CCTAAGCACCCGACAGAGAG		

Table S7. List of primers for off-target analysis; related to Supplemental Experimental Procedures on sequence analysis at off-targets.
Details of primers used in this study for off-target analysis in hiPSCs.

SUPPLEMENTAL EXPERIMENTAL PROCEDURES

Human pluripotent stem cell lines and culture

Human induced pluripotent stem cell (hiPSC) lines, used for generating neural stem cells (NSCs) and neurons, were established previously. These include control hiPSC lines, CT1 and CT2 (Pourtoy-Brasselet et al., 2021); and WS patient-derived hiPSC lines, WS1 (Shang et al., 2014), WS2 (Shang et al., 2014) and WS5 (Pourtoy-Brasselet et al., 2021). WS5R (rescued) hiPSC line was generated by genome editing via CRISPR/Cas9 mediated knock-in of *WFS1* cDNA under a doxycycline inducible promoter in the *AAVS1* locus of WS5 line, as previously established (Pourtoy-Brasselet et al., 2021). WIBR3 human embryonic stem cell (hESC) line was established previously (Lengner et al., 2010). The hiPSC and hESC lines were cultured, as previously described (Lengner et al., 2010; Pourtoy-Brasselet et al., 2021), on inactivated mouse embryonic fibroblast (MEF) feeder layer in hESC medium comprised of DMEM/F-12, 5 % KnockOut Serum Replacement, 1 % L-glutamine, 1 % non-essential amino acids, 1 % penicillin/streptomycin, 4 ng/mL human recombinant basic fibroblast growth factor (bFGF) (all from Gibco), 15 % fetal bovine serum (HyClone) and 0.1 mM β-mercaptoethanol (Sigma-Aldrich); or cultured feeder-free on Geltrex basement membrane matrix in StemFlex Basal Medium supplemented with StemFlex 10X Supplement (all from Gibco); and maintained in a humidified incubator with 5 % CO_2 and 5 % O_2 at 37 °C.

Generation and culture of hiPSC-derived neural stem cells

Neural stem cells (NSCs) were differentiated from hiPSCs, as described previously (Boissart et al., 2013; Pourtoy-Brasselet et al., 2021). NSCs were cultured on Poly-L-ornithine and Laminin (PO-L) (Sigma-Aldrich) coated plates or flasks in N2B27 medium comprised of DMEM/F-12 and Neurobasal medium in 1:1 ratio, 1 % N-2 supplement, 2 % B-27 supplement, 1% penicillin/streptomycin (all from Gibco), 0.1 % β-mercaptoethanol (Sigma-Aldrich) supplemented with 10 ng/mL FGF-2 (Miltenyi Biotec) and 10 ng/mL EGF (PeproTech), and were maintained in a humidified incubator with 5 % CO_2 at 37 °C. NSCs were passaged twice a week with 0.05 % Trypsin-EDTA (Gibco), and the medium was changed on alternate days. Approximately 6.5×10^4 cells/well were seeded in a 96-well plate for analyses of mitochondrial function or cell viability, 9×10^4 cells/well were seeded in 24-well plate with coverslips for immunostaining, and 4.5×10^5 cells/well were seeded in 6-well plate for immunoblotting and qPCR analyses. Analyses on NSCs were done after 48 h or after compound treatment.

Neuronal differentiation of hiPSC-derived NSCs

Neuronal differentiation of hiPSC-derived NSCs was carried out as described previously (Boissart et al., 2013; Pourtoy-Brasselet et al., 2021). The NSCs were seeded as above on PO-L coated plates in N2B27 medium without FGF-2 and EGF, and were maintained in a humidified incubator with 5 % CO_2 at 37 °C. At day 4 of neuronal differentiation, cells were treated with 10 µM DAPT (Tocris) to prevent cell proliferation. The N2B27 medium (without FGF-2 and EGF) was changed every 2 days and neuronal differentiation was carried out for 4 weeks (4 w), after which analyses were done for cellular identity, mitochondrial function and cell viability. The neurons generated *in vitro* were cortical in nature (Boissart et al., 2013; Pourtoy-Brasselet et al., 2021).

HEK293 cell culture and transfection

HEK293 cells were cultured in DMEM medium supplemented with 10 % FBS, 1 % L-glutamine, 1 % non-essential amino acids and 1 % penicillin/streptomycin (all from Gibco), and were maintained in a humidified incubator with 5 % CO_2 at 37 °C. The cells were plated at 5×10^5 cells per well in 6-well plates, and after 24 h, transient transfection was performed with FuGENE HD Transfection Reagent (Promega) using plasmids pCMV-Myc-WFS1 and pCMV-Myc (Zatyka et al., 2008) according to the manufacturer's protocol. Briefly, 3 µg of the plasmid was used per well with 3:1 ratio of FuGENE HD to DNA. Cells were incubated with the transfection mix for 48 h after which the samples were harvested for experimentation.

Compound treatment

For restoration of WFS1 levels, WS5R hiPSC-derived NSCs and neurons were treated with 50 ng/mL doxycycline (Dox; Sigma-Aldrich) for 48 h to induce WFS1 expression (Pourtoy-Brasselet et al., 2021). For modulating mitochondrial function, WS1 or WS2 hiPSC-derived NSCs and neurons were treated with 1 µM Cyclosporin A (CsA), 2 µM Mn(III)tetrakis(4-benzoic acid)porphyrin Chloride (MnTBAP), and 100 µM N-Acetyl-L-cysteine (NAC) (all from Sigma-Aldrich; details of the drugs in Table S1) for 48 h (in NSCs with replenishment on the first day) or for 6 days (in neurons with replenishment on the third day).

RNA-seq data analysis

AmpliSeq data used in this study has been previously published and deposited on NCBI GEO under accession number GSE156911 (Pourtoy-Brasselet et al., 2021). A subset of genes related to mitochondria were established from several pathways (WikiPathway 2021 Human / WP111; HumanCyc 2016 / PWY66-407; Reactome 2016 / R-HSA-163200) combined with Gene Ontologies (GO:0006084; GO:0010510; GO:0006390; GO:0030150; GO:0005741; GO:0000266; GO:0048311) followed by web-based gene list enrichment analysis tool EnrichR (Chen et al., 2013; Kuleshov et al., 2016). Commonly expressed upregulated and downregulated differentially expressed genes (DEGs) (*P* value ≤ 5 %; Fold change ≥ 1.5; Minimum reads > 100) between mitochondria gene-set of control and WS NSCs or neurons were selected using Venny diagram (v2.1.0) (Oliveros, 2007-2015). These mitochondria-associated DEGs in NSCs and neurons were depicted in a volcano plot by plotting the magnitude of change [Log_2(Fold change)] against the measure of significance [Log_{10}(*P* adjusted)].

Gene expression analysis

The expression of mitochondrial and neuronal genes was analyzed by quantitative real-time PCR (qPCR), as previously described (Araujo et al., 2020; Hummon et al., 2007). Briefly, total RNA was extracted using Trizol (Invitrogen), and the quality and concentration of mRNA was evaluated using NanoDrop ND-1000 spectrophotometer (Thermo Fisher Scientific). The RNA was converted to complementary DNA (cDNA) using iScript cDNA Synthesis Kit (Bio-Rad) according to manufacturer's instruction. 100 ng of cDNA was used for qPCR with 300 nM of gene-specific primers (primers sequences listed in Table S2) and SsoAdvanced Universal SYBR Green Supermix (Bio-Rad) using the Applied Biosystems QuantStudio 5 Real-Time PCR System (Thermo Fisher Scientific). The reaction comprised an initial cycle of 10 min at 95 °C, and then 40 subsequent cycles of 15 sec at 95 °C and 60 secs at 60 °C, followed by melting curve of 95 °C for 15 sec, 60 °C for 1 min and 95 °C for 15 sec. Data were analyzed using $2^{-\Delta\Delta Ct}$ method, normalized to the expression of the housekeeping gene *GAPDH* and expressed as a percentage of the control condition.

Immunoblotting analysis

For WFS1 immunoblotting, cells were lysed in Lysis Buffer [62.5 mM Tris-HCl pH 6.8, 2 % SDS, 12.5 % glycerol (all from Sigma-Aldrich) and Complete Mini Protease Inhibitor Cocktail (Roche)]. For all other immunoblotting, cells were lysed in RIPA Buffer [50 mM Tris pH 8, 150 mM NaCl, 0.1 % SDS, 1 mM EDTA, 0.5 % deoxycholate, 1 % IGEPAL (all from Sigma-Aldrich) and Complete Mini Protease Inhibitor Cocktail (Roche)]. The cell lysates were sonicated 3 × 10 s followed by centrifugation at 12000 × *g* for 30 min at 4 ºC. Protein concentration was measured by DC Protein Assay (Bio-Rad). The samples were boiled for all immunoblotting but not for WFS1 immunoblotting. 20 µg of protein per sample was subjected to SDS–PAGE and immunoblot analysis as described previously (Gharanei et al., 2013; Seranova et al., 2019; Sun et al., 2021). The immunoblots were then incubated in Blocking Buffer (5 % non-fat milk powder in PBS-Tween 20) for 1 h at room temperature, followed by incubation in primary antibodies overnight at 4 °C and then in appropriate secondary antibodies conjugated to horseradish peroxidase for 1 h at room temperature (list of primary and secondary antibodies for immunoblotting analysis in Table S3). The chemiluminescent signal was visualized using SuperSignal West Femto Maximum Sensitivity Substrate (Thermo Fisher Scientific) or Amersham ECL Western Blotting Detection Reagent (GE Healthcare) on Amersham Hyperfilm ECL (GE Healthcare) via ECOMAX X-ray Film Processor (PROTEC). Densitometry analysis on immunoblots was done using ImageJ v1.48 (NIH) software. The data was expressed as a percentage of the control condition, as previously described (Seranova et al., 2019; Sun et al., 2021).

Immunofluorescence analysis

Immunofluorescence analysis was performed as described previously (Seranova et al., 2019; Sun et al., 2021). Briefly, cells were washed in PBS, fixed with 4 % formaldehyde (Thermo Fisher Scientific) at room temperature for 15 min, permeabilized with 0.5 % Triton X-100 (Sigma-Aldrich) for 10 min, and incubated with Blocking Buffer [5 % donkey serum (Sigma-Aldrich) in PBS] for 1 h at room temperature. The cells were then incubated overnight with primary antibodies (diluted in Blocking Buffer) at 4 °C, then washed in PBS, followed by incubation with appropriate Alexa Fluor conjugated secondary antibodies (diluted in Blocking Buffer) for 1 h at room temperature (list of primary and secondary antibodies for immunofluorescence analysis in Table S4), and washed again in PBS. The coverslips were mounted on glass slides with ProLong Gold antifade reagent with DAPI (Invitrogen).

Staining of mitochondria-associated ER membranes (MAMs)

Analysis of MAMs by staining of mitochondria (with MitoTracker Red CMXRos dye) and ER (with Calnexin antibody) was done as described previously (Wang et al., 2021). The hiPSC-derived NSCs

were seeded into an initial density of 1–2×10^5 cells per well in 24-well plates with PO-L-coated cover slips. Neuronal differentiation was done for 4 weeks. The hiPSC-derived neurons were treated with or without 50 ng/mL doxycycline for 48 h. Cells were then incubated with 1 µM MitoTracker Red CMXRos (Invitrogen) in a humidified incubator with 5 % CO_2 at 37 °C, followed by immunostaining with Calnexin antibody and staining with 10 µg/mL DAPI (Invitrogen). The cover clips were mounted on Vectashield antifade mountant (Vector Laboratories).

Image acquisition of fixed cells
Immunofluorescence images of fixed cells were acquired by fluorescence microscopy using EVOS FL Cell Imaging System (Thermo Fisher Scientific) with AMG 10x Plan FL and AMG 40x Plan FL lens, or with Zeiss LSM880 confocal microscope with Airyscan (Zeiss) equipped with a 100x/1.4 PlanApo objective. Confocal microscope images were acquired and processed with the Zeiss ZEN (Black) software. For analyses of MAMs and mitochondrial branch length, images of fixed cells were acquired by structured illumination microscopy (SIM) using a Nikon N-SIM System (Nikon Instruments) equipped with a SR HP Apo TIRF 100x/1.49 objective and two Hamamatsu ORCA Flash4 CMOS cameras. The Nikon NIS Element software was used to acquire and process SIM images for analysis. The colocalization and colour threshold panels of images were generated using ImageJ v1.48 (NIH) software.

Image analysis for quantification of MAMs
For quantification of MAMs, colocalization analysis between MitoTracker Red CMXRos and Calnexin was performed on SIM reconstructed images using JacCoP plugin (https://imagej.net/plugins/jacop) within the Fiji/ImageJ software. The JACoP plugin allows to use several commonly used colocalization indicators. Among those, Mander's coefficient was used because it shows the percentage of pixel that overlaps between two channels (Dunn et al., 2011), therefore quantifying the colocalization between mitochondria and ER. The coefficient values range between 0 and 1, expressing the ratio of intensity of positive pixels (or threshold) from one channel into another one. Image analysis for the quantification of MAMs was performed on ~75 images per sample, and the data on Mander's coefficient was expressed as a percentage. The cells imaged for MAM analysis were selected based on their neuronal morphology.

Mitochondrial branch length analysis
Mitochondrial branch length analysis after MitoTracker Red CMXRos staining (as described above) was done, as previously described (Valente et al., 2017), on SIM reconstructed images. Measurements of mitochondrial branch length, mitochondrial summed branch length and mitochondrial footprint were done in hiPSC-derived neurons (~50 images per sample) using Analyze Morphology plugin from Mitochondrial Network Analysis (MiNA) toolset in Fiji v2.9.0 (Schindelin et al., 2012) with the following settings: (i) Median filter: radius = 4; (ii) Unsharp mask: radius = 3, mask weight = 0.7; (iii) CLAHE: blocksize = 99, histogram bins = 200, max slope = 3, mask = none. Otsu Thresholding and Ridge Detection were selected with the following settings: High contrast = 250; Low contrast = 140; Line width = 15; Minimum line length = 1. The hiPSC-derived neurons were identified based on their morphology, and non-neuronal cells or artifacts were removed using a mask in ImageJ prior to MiNA analysis.

Co-immunoprecipitation
HEK293 cell lysate (600 µg) overexpressing either pCMV-Myc-WFS1 (Myc-WFS1) or pCMV-Myc (empty Myc), or WS5R hiPSC-derived NSC lysate in the presence or absence of Doxycycline, was used for co-immunoprecipitation (co-IP) with 2.35 µg of either WFS1 antibody (rabbit) or rabbit IgG (two different rabbit IgG were used) from non-immunised animals (list of primary antibodies for immunoprecipitation in Table S5). Co-IP was performed with Dynabeads Protein A Immunoprecipitation Kit (Invitrogen) as per manufacturer's instructions with minor modifications. The cell lysates mixed with antibody and magnetic beads were incubated for 30 min at room temperature on a rotation wheel followed by 6 washes in wash buffer. The elution samples (IP samples) as well as the whole cell lysates (inputs) were subjected to immunoblotting analysis with VDAC1 (mouse) antibody, followed by reprobing of the immunoblot with WFS1 (sheep) and c-Myc (mouse) antibodies (list of primary and secondary antibodies for immunoblotting analysis in Table S3).

Identification of WFS1 interactors by immunoprecipitation and mass spectrometry
HEK293 cell lysate (1 mg protein) overexpressing Myc-WFS1 (Zatyka et al., 2008) was used for immunoprecipitation (IP) with 5 µg of WFS1 antibody (rabbit) or with 5 µg of rabbit IgG from non-immunised animals (used as a negative control). The Dynabeads Protein A Immunoprecipitation Kit

(Invitrogen) was used for IP following the manufacturer's protocol. The immunoprecipitated proteins were eluted in SDS loading buffer by heating at 95 °C for 10 min, separated by SDS–PAGE on a 4–20 % Mini-Protean Precast Protein Gel (Bio-Rad), followed by staining with Bio-Safe Coomassie Stain (Bio-Rad) for 1 h and then de-staining in water overnight. The protein bands were cut from the stained gel and subjected to in-gel digestion using sequencing grade trypsin (Promega). The peptides were extracted with 1 % formic acid in 10 % acetonitrile for 60 min at room temperature and then with 2 % formic acid in 60 % acetonitrile for 30 min at room temperature, and lyophilised. The peptides were dissolved in 0.1 % formic acid. The resulting peptides were analysed by LC-MS/MS using a 60 min 0–40 % acetonitrile gradient in 0.1 % formic acid (75 μm × 25 cm C18 Pepmap column, Dionex) and a Bruker Impact Q-ToF Mass Spectrometer (Bruker Daltonics). Peptides were identified using MASCOT to search the SWISSPROT human protein sequence database. Mass tolerances for parent and fragment ions were 20 p.p.m. and 0.05 Da, respectively, and the minimum peptide MOWSE score was 25. Protein identifications were filtered using both a 1 % false discovery threshold and a requirement for two or more peptides using ProteinScape software (Bruker Daltonics). Mass spectrometry data for WFS1 interactors has been deposited in MassIVE repository, accession number MSV000091646.

Mitochondrial $\Delta\Psi_m$, ROS and Ca^{2+} measurements
Measurements of mitochondrial membrane potential ($\Delta\Psi_m$), reactive oxygen species (ROS) and mitochondrial Ca^{2+} were respectively done using TMRE (Tetramethylrhodamine ethyl ester), CM-H_2DCF-DA (chloromethyl derivative of 2',7'-dichlorodihydrofluorescein diacetate) and Fluo-3 AM (all from Invitrogen), as described previously (Araujo et al., 2020; Rosenstock et al., 2022; Silva et al., 2019). Briefly, cells were loaded with Microscopy Medium (120 mM NaCl, 3.5 mM KCl, 0.4 mM KH_2PO_4, 5 mM $NaHCO_3$, 1.2 mM $NaSO_4$, 20 mM HEPES and 15 mM glucose; pH 7.4) supplemented with 1 mM $CaCl_2$ (all from Sigma-Aldrich), along with 1 % Pluronic F-127 (Thermo Fisher Scientific) for Fluo-3 AM only, and incubated with 500 nM TMRE (for $\Delta\Psi_m$), 20 µM CM-H_2DCFDA (for ROS) or 10 µM Fluo-3 AM (for mitochondrial Ca^{2+}) for 1 h at 37 ºC. The fluorescence signals of TMRE, CM-H_2DCFDA and Fluo-3 AM were acquired using EnSpire Multimode microplate reader (Perkin Elmer) for a period of 5 min to get basal fluorescence, and again for TMRE and Fluo-3 AM for another 5 min after the addition of the mitochondrial uncoupler, 10 µM FCCP (fluorocarbonyl cyanide phenylhydrazone; Sigma-Aldrich). The delta (Δ) values of TMRE and Fluo-3 AM, denoting mitochondrial $\Delta\Psi_m$ and mitochondrial Ca^{2+}, were calculated by subtracting the basal fluorescence from post-FCCP fluorescence (Araujo et al., 2020; Rosenstock et al., 2022; Silva et al., 2019). Data were obtained as relative fluorescence units, normalised to protein concentration by Bradford Protein Assay (Bio-Rad) and expressed as a percentage of the control condition.

ATP measurement
ATP levels were measured using ApoSENSOR ADP/ATP Ratio Bioluminescent Assay Kit (BioVision) as per manufacturer's instructions (Rosenstock et al., 2022). Briefly, the Reaction Mix containing Nucleotide Releasing Buffer and ATP monitoring enzyme was added into a 96-well, white-walled, clear flat-bottom optical plate and incubated at room temperature for 1 h to burn the residual ATP levels. The luminescence was then measured using EnSpire Multimode microplate reader (Perkin Elmer) to determine the background reading (Data A). Cells cultured in a separate 96-well plate were incubated with Nucleotide Releasing Buffer for 5 min at room temperature to release the ATP. The supernatant was then transferred to the appropriate wells of the 96-well white-walled plate containing the Reaction Mix, and incubated for 2 min at room temperature. The luminescence was measured again using microplate reader (Data B). ATP levels were determined by subtracting Data B from Data A, normalized to protein concentration by Bradford Protein Assay (Bio-Rad), and expressed as a percentage of control condition.

Mitochondrial respiration measurement
The hiPSC-derived NSCs were seeded into an initial density of 4–4.5×10^4 cells per well in XF96 cell-culture microplates previously coated with PO-L. The NSCs were cultured for 48 h (for measurement in NSCs) whilst the neurons were generated from NSCs after differentiation for 4 weeks (for measurement in neurons), amounting to ~3.5×10^4 cells per well in XF96 cell-culture microplates. Before the experiment, the original culture medium was replaced with Seahorse XF DMEM medium without phenol red supplemented with 2.5 mM L-glutamine, 0.5 mM sodium pyruvate and 17.5 mM glucose (all from Agilent; supplemented to match the levels of these components in the DMEM/F-12 medium in which the neuronal cells were cultured), and the cells were incubated for 1 h in a non-CO_2 incubator. Preparation of all the regents was done while the cells were in the incubation period and following the manufacturer's instructions. Basal levels of oxygen consumption rates (OCR) were measured on an XFe96 Extracellular Flux Analyzer (Agilent). Cells were stimulated with 2 µM

oligomycin, 3 μM BAM15 and 1 μM rotenone/antimycin A (all from Sigma-Aldrich), following the instructions specified in the XF Cell Mito Stress Test Kit (Agilent). A range of metabolic parameters were calculated, such as basal respiration, ATP production, proton leak and maximal respiration (calculations described in Table S6). CyQUANT Direct Cell Proliferation Assay (Invitrogen) was used to normalise cell number following the manufacturer's instructions. Fluorescence was measured in a FLUOstar Omega Plate Reader (BMG Labtech). We also utilised a protein normalisation method with Bradford Protein Assay (Bio-Rad) to corroborate that our normalisation method was accurate. The saturation dynamics for Seahorse was not at maximum level, and the levels were within a dynamic range. Biological replicates represent each well of the Seahorse plates, arising from 3 independent experiments performed on different days whilst keeping the same conditions as the other days.

Cell viability measurement by cytotoxicity assay
Cell viability was measured by luminescence-based CytoTox-Glo Cytotoxicity Assay (Promega) as per manufacturer's protocol. This luminescence-based cytotoxicity assay measures the extracellular activity of a distinct dead-cell protease when it is released from membrane-compromised cells. Briefly, cells in 96-well, white-walled, clear flat-bottom optical plates were incubated with CytoTox-Glo Assay Reagent (comprising of Assay Buffer and AAF-Glo Substrate) for 15 min at room temperature in the dark. The luminescence was measured using EnSpire Multimode microplate reader (Perkin Elmer). The readings obtained were attributed to the basal cytotoxicity (first reading). Cells were further incubated with Lysis Reagent (comprising of Assay Buffer and Digitonin) for 30 min at room temperature in the dark, after which luminescence was measured again (second reading). Cell viability was determined by subtracting the first reading from the second reading according to manufacturer's instructions, and expressed as percentage of the control condition.

TUNEL assay for apoptotic cells
Cells were stained with Click-iT Plus TUNEL Assay for *in situ* apoptosis detection, Alexa Fluor 488 dye (Invitrogen), as per manufacturer's protocol. Briefly, cells were fixed with 4 % formaldehyde (Thermo Fisher Scientific) for 15 min, permeabilized with 0.25 % Triton X-100 (Sigma-Aldrich) for 20 min at room temperature and washed with deionized water. Cells were incubated in TdT reaction buffer for 10 min at 37 °C, further incubated in TdT reaction mixture (comprising of TdT reaction buffer, EdUTP and TdT enzyme) for 60 min at 37 °C, washed with 3 % BSA (in PBS), then incubated in Click-iT Plus TUNEL reaction cocktail for 30 min at 37 °C, and washed again with 3 % BSA. To detect TUNEL$^+$ apoptotic nuclei only in neurons, cells were subjected to immunofluorescence by blocking with 3 % BSA, incubating with TUJ1 antibody (in 3% BSA) overnight at 4 °C, and then incubating with Alexa Fluor 594 secondary antibody for 1 h at room temperature. Coverslips were mounted on glass slides with ProLong Gold antifade reagent with DAPI (Invitrogen), followed by analysis by fluorescence microscopy as previously described (Maetzel et al., 2014). The percentage of TUNEL$^+$ nuclei was calculated from the total number of TUJ1$^+$ cells analysed. Approximately 200–300 cells per sample were analysed.

Sequence analysis at off-targets
Genomic DNA was extracted from WS5 and WS5R hiPSCs using QIAamp DNA Blood Kits (Qiagen). PCR reactions were carried out with 50 ng genomic DNA using the Phusion High-Fidelity DNA Polymerase Kit (Thermo Fisher Scientific) as per manufacturer's instruction (primers sequences are listed in Table S7). PCR conditions were 30 s at 98°C, followed by 30 cycles of 10 s at 98 °C, 30 s at 63 °C and 15 s at 72 °C, then 5 min at 72 °C. PCR products were sent for sequencing to Genewiz. To determine possible off-target loci, CRISPOR tool was used and the top five loci were sequenced.

Statistical analysis
Graphical data are shown from 3 or more biological replicates from independent experiments, as indicated in the respective figure legends. Graphical data are depicted by column graph scatter dot plot (mean ± s.e.m.) or violin plot (line at median) using Prism v8.3.1 software (GraphPad). Quantification of data are described under various Methods sections where appropriate. Statistical significance (P value) on graphical data was determined using unpaired two-tailed Student's *t*-test or by one-way ANOVA with Tukey's or Dunnett's multiple comparisons test using Prism v8.3.1 software (GraphPad). *$P<0.05$; **$P<0.01$; ***$P<0.001$; ns, non-significant.

SUPPLEMENTAL REFERENCES

Araujo, B.G., Souza, E.S.L.F., de Barros Torresi, J.L., Siena, A., Valerio, B.C.O., Brito, M.D., and Rosenstock, T.R. (2020). Decreased mitochondrial function, biogenesis, and degradation in peripheral blood mononuclear cells from amyotrophic lateral sclerosis patients as a potential tool for biomarker research. Mol Neurobiol *57*, 5084-5102. 10.1007/s12035-020-02059-1.

Boissart, C., Poulet, A., Georges, P., Darville, H., Julita, E., Delorme, R., Bourgeron, T., Peschanski, M., and Benchoua, A. (2013). Differentiation from human pluripotent stem cells of cortical neurons of the superficial layers amenable to psychiatric disease modeling and high-throughput drug screening. Transl Psychiatry *3*, e294. 10.1038/tp.2013.71.

Chen, E.Y., Tan, C.M., Kou, Y., Duan, Q., Wang, Z., Meirelles, G.V., Clark, N.R., and Ma'ayan, A. (2013). Enrichr: interactive and collaborative HTML5 gene list enrichment analysis tool. BMC Bioinformatics *14*, 128. 10.1186/1471-2105-14-128.

Dunn, K.W., Kamocka, M.M., and McDonald, J.H. (2011). A practical guide to evaluating colocalization in biological microscopy. Am J Physiol Cell Physiol *300*, C723-742. 10.1152/ajpcell.00462.2010.

Gharanei, S., Zatyka, M., Astuti, D., Fenton, J., Sik, A., Nagy, Z., and Barrett, T.G. (2013). Vacuolar-type H+-ATPase V1A subunit is a molecular partner of Wolfram syndrome 1 (WFS1) protein, which regulates its expression and stability. Hum Mol Genet *22*, 203-217. 10.1093/hmg/dds400.

Hummon, A.B., Lim, S.R., Difilippantonio, M.J., and Ried, T. (2007). Isolation and solubilization of proteins after TRIzol extraction of RNA and DNA from patient material following prolonged storage. Biotechniques *42*, 467-470, 472. 10.2144/000112401.

Kuleshov, M.V., Jones, M.R., Rouillard, A.D., Fernandez, N.F., Duan, Q., Wang, Z., Koplev, S., Jenkins, S.L., Jagodnik, K.M., Lachmann, A., et al. (2016). Enrichr: a comprehensive gene set enrichment analysis web server 2016 update. Nucleic Acids Res *44*, W90-97. 10.1093/nar/gkw377.

Lengner, C.J., Gimelbrant, A.A., Erwin, J.A., Cheng, A.W., Guenther, M.G., Welstead, G.G., Alagappan, R., Frampton, G.M., Xu, P., Muffat, J., et al. (2010). Derivation of pre-X inactivation human embryonic stem cells under physiological oxygen concentrations. Cell *141*, 872-883. 10.1016/j.cell.2010.04.010.

Maetzel, D., Sarkar, S., Wang, H., Abi-Mosleh, L., Xu, P., Cheng, A.W., Gao, Q., Mitalipova, M., and Jaenisch, R. (2014). Genetic and chemical correction of cholesterol accumulation and impaired autophagy in hepatic and neural cells derived from Niemann-Pick Type C patient-specific iPS cells. Stem Cell Reports *2*, 866-880. 10.1016/j.stemcr.2014.03.014.

Oliveros, J.C. (2007-2015). Venny. An interactive tool for comparing lists with Venn's diagrams. http://bioinfogp.cnb.csic.es/tools/venny/index.html.

Pourtoy-Brasselet, S., Sciauvaud, A., Boza-Moran, M.G., Cailleret, M., Jarrige, M., Polveche, H., Polentes, J., Chevet, E., Martinat, C., Peschanski, M., and Aubry, L. (2021). Human iPSC-derived neurons reveal early developmental alteration of neurite outgrowth in the late-occurring neurodegenerative Wolfram syndrome. Am J Hum Genet *108*, 2171-2185. 10.1016/j.ajhg.2021.10.001.

Rosenstock, T.R., Sun, C., Hughes, G.W., Winter, K., and Sarkar, S. (2022). Analysis of mitochondrial dysfunction by microplate reader in hiPSC-derived neuronal cell models of neurodegenerative disorders. Methods Mol Biol. *2549*, 1-21. 10.1007/7651_2021_451.

Schindelin, J., Arganda-Carreras, I., Frise, E., Kaynig, V., Longair, M., Pietzsch, T., Preibisch, S., Rueden, C., Saalfeld, S., Schmid, B., et al. (2012). Fiji: an open-source platform for biological-image analysis. Nat Methods *9*, 676-682. 10.1038/nmeth.2019.

Seranova, E., Ward, C., Chipara, M., Rosenstock, T.R., and Sarkar, S. (2019). In vitro screening platforms for identifying autophagy modulators in mammalian cells. Methods Mol Biol *1880*, 389-428. 10.1007/978-1-4939-8873-0_26.

Shang, L., Hua, H., Foo, K., Martinez, H., Watanabe, K., Zimmer, M., Kahler, D.J., Freeby, M., Chung, W., LeDuc, C., et al. (2014). beta-cell dysfunction due to increased ER stress in a stem cell model of Wolfram syndrome. Diabetes *63*, 923-933. 10.2337/db13-0717.

Silva, L.F.S.E., Brito, M.D., Yuzawa, J.M.C., and Rosenstock, T.R. (2019). Mitochondrial dysfunction and changes in high-energy compounds in different cellular models associated to hypoxia: Implication to schizophrenia. Sci Rep *9*, 18049. 10.1038/s41598-019-53605-4.

Sun, C., Rosenstock, T.R., Cohen, M.A., and Sarkar, S. (2021). Autophagy dysfunction as a phenotypic readout in hiPSC-derived neuronal cell models of neurodegenerative diseases. Methods Mol Biol. *2549*, 103-136. 10.1007/7651_2021_420.

Valente, A.J., Maddalena, L.A., Robb, E.L., Moradi, F., and Stuart, J.A. (2017). A simple ImageJ macro tool for analyzing mitochondrial network morphology in mammalian cell culture. Acta Histochem *119*, 315-326. 10.1016/j.acthis.2017.03.001.

Wang, C., Dai, X., Wu, S., Xu, W., Song, P., and Huang, K. (2021). FUNDC1-dependent mitochondria-associated endoplasmic reticulum membranes are involved in angiogenesis and neoangiogenesis. Nat Commun *12*, 2616. 10.1038/s41467-021-22771-3.

Zatyka, M., Ricketts, C., da Silva Xavier, G., Minton, J., Fenton, S., Hofmann-Thiel, S., Rutter, G.A., and Barrett, T.G. (2008). Sodium-potassium ATPase 1 subunit is a molecular partner of Wolframin, an endoplasmic reticulum protein involved in ER stress. Hum Mol Genet *17*, 190-200. 10.1093/hmg/ddm296.
