# Supplementary material for: Depletion of WFS1 compromises mitochondrial function in hiPSC-derived neuronal models of Wolfram syndrome
Source: Stem Cell Reports. 2023 May 9;18(5):1090–106. doi: 10.1016/j.stemcr.2023.04.002 (PMC10202695; doi:10.1016/j.stemcr.2023.04.002)
Supplement: Document S2. Article plus supplemental information [file mmc2.pdf]

Stem Cell Reports
Article
OPEN ACCESS

Depletion of WFS1 compromises mitochondrial function in hiPSC-derived neuronal models of Wolfram syndrome

Malgorzata Zatyka,[1,11] Tatiana R. Rosenstock,[1,10,11] Congxin Sun,[1] Adina M. Palhegyi,[1] Georgina W. Hughes,[1] Samuel Lara-Reyna,[2] Dewi Astuti,[1] Alessandro di Maio,[3] Axel Sciauvaud,[4,5] Miriam E. Korsgen,[1] Vesna Stanulovic,[1] Gamze Kocak,[1] Malgorzata Rak,[6] Sandra Pourtoy-Brasselet,[7] Katherine Winter,[1] Thiago Varga,[1] Margot Jarrige,[4,5,7] Hélène Polvèche,[7] Joao Correia,[8] Eva-Maria Frickel,[2] Maarten Hoogenkamp,[1] Douglas G. Ward,[1] Laetitia Aubry,[4,5] Timothy Barrett,[1,9] and Sovan Sarkar[1,*]
[1]Institute of Cancer and Genomic Sciences, Institute of Biomedical Research, College of Medical and Dental Sciences, University of Birmingham, Edgbaston, Birmingham B15 2TT, UK
[2]Institute of Microbiology and Infection, University of Birmingham, Birmingham B15 2TT, UK
[3]Tech Hub Microscopy Facility, College of Medical and Dental Sciences, University of Birmingham, Birmingham B15 2TT, UK
[4]INSERM UMR 861, I-STEM, AFM, 91100 Corbeil-Essonnes, France
[5]Université Paris-Saclay, INSERM, University Evry, Institut des cellules Souches pour le Traitement et l'Etude des maladies Monogéniques, 91100 Corbeil-Essonnes, France
[6]Université Paris Cité, INSERM, NeuroDiderot, 75019 Paris, France
[7]CECS/AFM, I-STEM, 91100 Corbeil-Essonnes, France
[8]COMPARE Advanced Imaging Facility, College of Medical and Dental Sciences, University of Birmingham, Birmingham B15 2TT, UK
[9]Department of Endocrinology, Birmingham Women's and Children's Hospital, Steelhouse Lane, Birmingham B4 6NH, UK
[10]Present address: Sygnature Discovery, Nottingham, United Kingdom
[11]These authors contributed equally
*Correspondence: s.sarkar@bham.ac.uk
https://doi.org/10.1016/j.stemcr.2023.04.002

SUMMARY

Mitochondrial dysfunction involving mitochondria-associated ER membrane (MAM) dysregulation is implicated in the pathogenesis of late-onset neurodegenerative diseases, but understanding is limited for rare early-onset conditions. Loss of the MAM-resident protein WFS1 causes Wolfram syndrome (WS), a rare early-onset neurodegenerative disease that has been linked to mitochondrial abnormalities. Here we demonstrate mitochondrial dysfunction in human induced pluripotent stem cell-derived neuronal cells of WS patients. VDAC1 is identified to interact with WFS1, whereas loss of this interaction in WS cells could compromise mitochondrial function. Restoring WFS1 levels in WS cells reinstates WFS1-VDAC1 interaction, which correlates with an increase in MAMs and mitochondrial network that could positively affect mitochondrial function. Genetic rescue by WFS1 overexpression or pharmacological agents modulating mitochondrial function improves the viability and bioenergetics of WS neurons. Our data implicate a role of WFS1 in regulating mitochondrial functionality and highlight a therapeutic intervention for WS and related rare diseases with mitochondrial defects.

INTRODUCTION

Neurodegenerative diseases are characterized by gradual loss of neuronal function and viability. Multiple studies have demonstrated a crucial role of impairment of mitochondrial homeostasis in the pathogenesis of common neurodegenerative diseases like Alzheimer's disease (AD) and Parkinson's disease (PD) and in certain rare neurodegenerative diseases (Johri and Beal, 2012). Mitochondria perform essential cellular functions in the regulation of bioenergetics, ion homeostasis, metabolism, and apoptosis and form dynamic networks that make contacts with other cellular organelles (Nunnari and Suomalainen, 2012). The interaction domains between mitochondria and endoplasmic reticulum (ER) are known as mitochondria-associated ER membranes (MAMs), which are involved in the regulation of mitochondrial biogenesis and dynamics, Ca^{2+} transfer, phospholipid synthesis and exchange, and cell death (Delprat et al., 2018; Giorgi et al., 2015). Dysfunction of MAM has been implicated in the pathogenesis of various neurodegenerative diseases including AD and PD (Paillusson et al., 2016). One of the proteins in MAM was found to be WFS1 (Wolfram syndrome 1; also called wolframin) (Delprat et al., 2018; Horner et al., 2015; La Morgia et al., 2020; Poston et al., 2013; Zhang et al., 2011), loss of function of which causes a rare, early-onset neurodegenerative disorder called Wolfram syndrome (WS) with no effective cure (Abreu and Urano, 2019; Barrett et al., 1995; Inoue et al., 1998). Loss of WFS1 protein in WS is associated with brain and optic nerve atrophy, diabetes, deafness, psychosis, and depression (Barrett et al., 1995; Rigoli et al., 2018), and these neurological and psychiatric defects resemble mitochondrial disease-like symptoms (Bu and Rotter, 1993).

Multiple lines of evidence arising from studies in immortalized, non-human or non-clinical cell lines suggest that mitochondrial functionality could be affected in WS associated with *WFS1* mutations. In immortalized human embryonic kidney (HEK) cells, siRNA-mediated *WFS1* knockdown caused upregulation of genes related to

 © 2023 The Author(s).
This is an open access article under the CC BY license (http://creativecommons.org/licenses/by/4.0/).

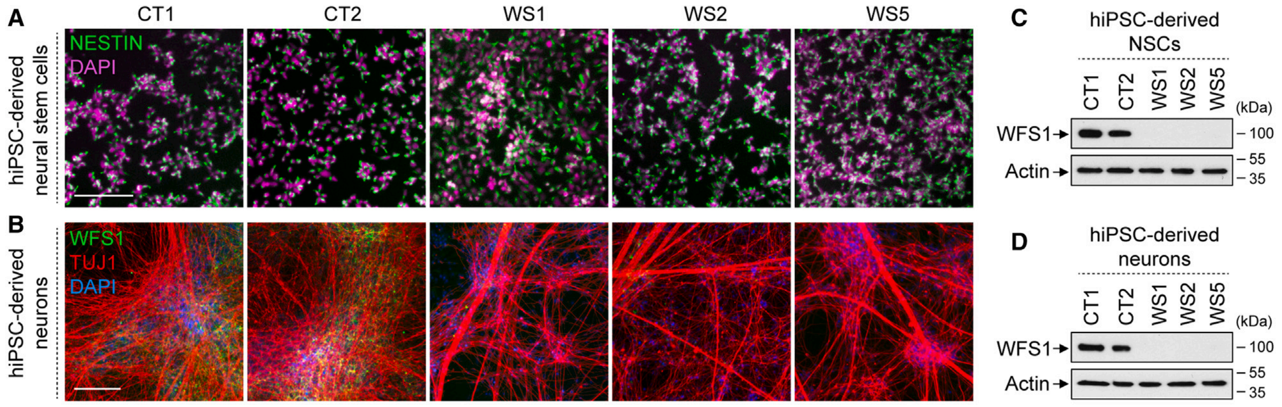

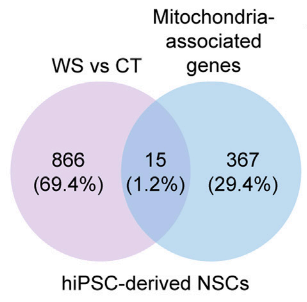

Common gene	Fold change	*P* value
BCL2	1.68	0.0020
LRPPRC	1.65	0.0149
TIMM23B	1.52	0.0249
SCO2	-1.77	0.0088
PMAIP1	-1.73	0.0036
SDHB	-1.70	0.0026
NDUFA10	-1.69	0.0314
BAD	-1.66	0.0003
FIS1	-1.65	0.0005
PINK1	-1.62	0.0387
NDUFS7	-1.57	0.0039
TPI1	-1.57	0.0177
UBB	-1.53	0.0272
TRAK2	-1.51	0.0035
COX16	-1.51	0.0168

Color scale: 2, 0, -2

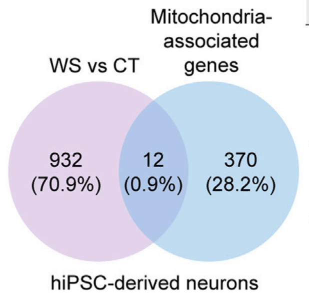

Common gene	Fold change	*P* value
NDUFV2	2.66	0.0330
MEF2A	1.98	0.0086
KRAS	1.69	0.0186
TRAK1	1.66	0.0109
NDUFAF1	-6.96	0.0449
CPT1B	-2.58	0.0164
SUCLG1	-2.05	0.0062
NDUFA11	-1.86	0.0164
COX8A	-1.77	0.0376
DNM2	-1.77	0.0498
SDHB	-1.65	0.0401
ATP5PD	-1.63	0.0386

Color scale: 3, 0, -3, -7

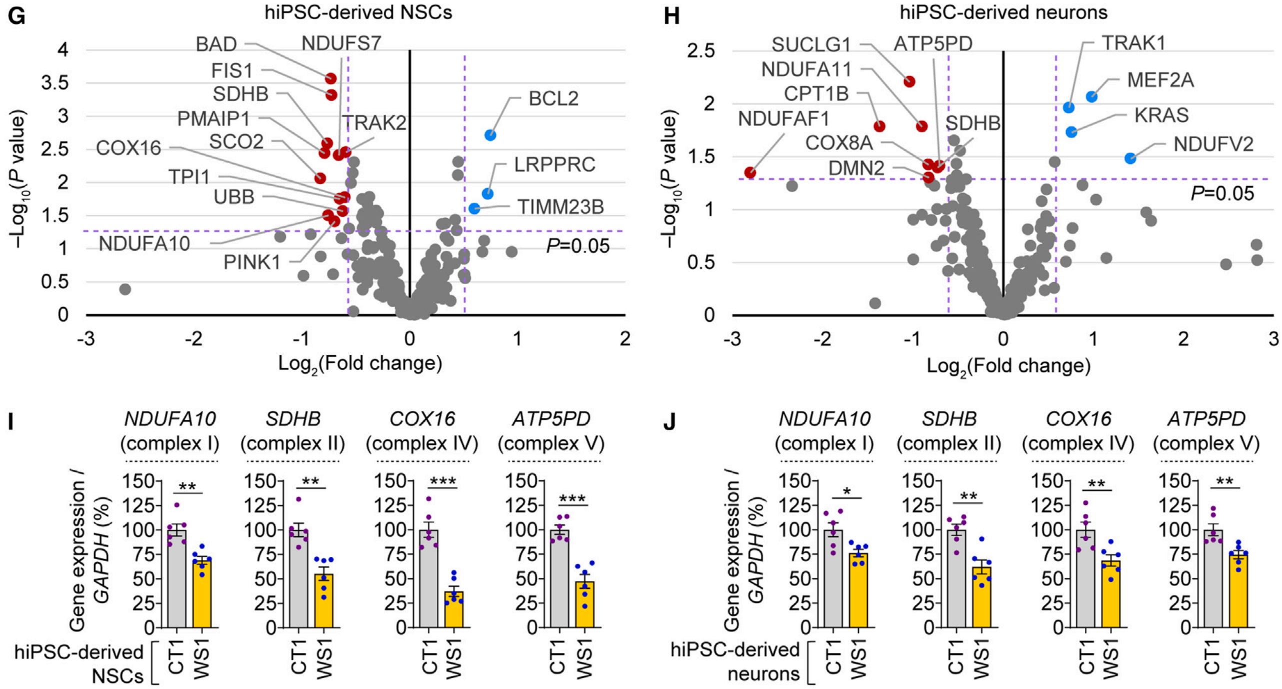

(legend on next page)

mitochondrial damage (Koks et al., 2013). Similarly, downregulation of WFS1 in primary cortical neurons of rat with shRNA-mediated *Wfs1* knockdown or of *Wfs1*-deficient mice impaired mitochondrial dynamics, which was associated with perturbation in neuronal function (Cagalinec et al., 2016). The underlying mechanism of alteration in mitochondrial dynamics was suggested to be arising from ER stress-mediated dysregulation of Ca^{2+} homeostasis (Cagalinec et al., 2016). A subsequent study using WS patient fibroblasts has further shown that loss of WFS1 disrupted its association with the neuronal calcium sensor 1 (NCS1) and inositol 1,4,5-trisphosphate receptor (IP_3R), which in turn diminished Ca^{2+} transfer between ER and mitochondria to cause mitochondrial deregulation (Angebault et al., 2018). In this study, WS patient fibroblasts exhibited reduction in mitochondrial respiration and complex I activity, but mitochondrial membrane potential ($\Delta\Psi_m$) was unaffected (Angebault et al., 2018). However, another study in WS patient fibroblasts reported no changes in mitochondrial respiration, $\Delta\Psi_m$, or network morphology (La Morgia et al., 2020). On the contrary, increased mitochondrial bioenergetics were reported in the quadriceps muscle fibers of *Wfs1*-deficient mice (Eimre et al., 2018), which also had higher basal oxygen consumption (Ehrlich et al., 2016). Apart from WFS1, mutations in another MAM-resident protein CISD2 associated with Wolfram syndrome type 2 caused mitochondrial abnormalities in mouse models of *Cisd2* deficiency (Chen et al., 2009; Delprat et al., 2018; Wiley et al., 2013). Moreover, mitochondrial DNA deletions were found in some WS patients (Barrientos et al., 1996). Overall, these studies show contradictory mitochondrial functionality with varied phenotypic readouts in WS in a context-dependent manner, and therefore it is pertinent to study mitochondrial function in patient-derived disease-affected cells for biomedical exploitation.

Here we investigated the impact of loss of WFS1 protein on mitochondria-associated gene expression and mitochondrial function, and its potential mechanism and consequence on cell survival, in neural stem cells (NSCs) and cortical neurons differentiated from WS patient-derived human induced pluripotent stem cells (hiPSCs). Such patient hiPSC-derived neuronal models potentially serve as pre-clinical, disease-relevant cellular platforms for studying disease mechanisms and identifying drug candidates (Avior et al., 2016). In this context, we also assessed the therapeutic efficacy of pharmacological agents modulating mitochondrial function on neuronal viability.

RESULTS

Deregulation of mitochondria-associated genes in WS hiPSC-derived neuronal cells

To study mitochondrial function in clinically relevant WS cellular platforms, we generated NSCs and neurons from previously established hiPSC lines derived from three WS patients (WS1, WS2, WS5) along with two healthy individuals as controls (CT1, CT2) (Boissart et al., 2013; Pourtoy-Brasselet et al., 2021; Shang et al., 2014) (Figure S1A). Cellular identities of hiPSC-derived cells were confirmed by immunofluorescence and gene expression analyses of cell-specific markers, such as NESTIN and PAX6 for NSCs, and TUJ1, MAP2, and NeuN for neurons (Figures 1A, 1B, and S1B–S1D). Our neuronal differentiation method generated neurons of cortical nature (Pourtoy-Brasselet et al., 2021), as evident from the gene expression of *POU3F2*, *CUX1*, and *TBR1* that are specific for cortical neurons and by TBR1 immunostaining in $TUJ1^+$ neurons (Figures S1E and S1F). Since disease-associated mutations in *WFS1* cause the protein to be unstable and degraded by the proteasome (Guo et al., 2011), WFS1 was detected only in control NSCs and neurons but not in WS cells (Figures 1B–1D).

In order to study the implication of mitochondria in WS pathogenesis, we established a list of 382 mitochondria-associated genes and compared the expression of these genes in three CT (control) and three WS hiPSC-derived NSCs and neurons using our previously published RNA-seq datasets (Pourtoy-Brasselet et al., 2021). Among 881 and 944 differentially expressed genes (DEGs) identified in WS compared with CT respectively in NSCs and

Figure 1. Deregulation of mitochondrial genes in WS patient hiPSC-derived NSCs and neurons

(A–D) Immunofluorescence images of NESTIN (A), WFS1 and TUJ1 (B), and immunoblotting analysis of WFS1 (C, D) in control (CT1, CT2) and WS patient (WS1, WS2, WS5) hiPSC-derived NSCs (A, C) and 4-week-old (4 w) neurons (B, D).

(E and F) Venn diagram summarizing the number of differentially expressed genes (DEGs) specific or common to mitochondria-associated genes set in NSCs (E) or neurons (4 w; F), and table of mitochondria-associated genes significantly deregulated in WS NSCs (E) and neurons (4 w; F), compared with CT.

(G–J) Volcano plot representation of mitochondria-associated genes analyzed by RNA-seq (G, H; thresholds shown as dashed purple lines), and qPCR expression analyses of *NDUFA10*, *SHDB*, *COX16*, and *ATP5PD* relative to *GAPDH* (I, J), in CT and WS hiPSC-derived NSCs (G, I) and neurons (4 w; H, J).

Graphical data are mean ± SEM of n = 6 biological replicates (I, J). p values were calculated by unpaired two-tailed Student's t test on three independent experiments (I, J). DEGs were identified via Partek gene-specific analysis (GSA) algorithm (p value ≤ 5%; fold change ≥ 1.5; minimum reads > 100) (E, F). *p < 0.05; **p < 0.01; ***p < 0.001. Scale bar, 100 μm (A, B). See also Figure S1.

neurons, a number of mitochondria-associated DEGs were found in WS NSCs and neurons (p value $\leq$ 5%; fold change $\geq$ 1.5; minimum reads > 100) (Figures 1E and 1F). Among the mitochondria gene set, there were 15 DEGs in WS NSCs (3 upregulated, 12 downregulated) and 12 DEGs in WS neurons (4 upregulated, 8 downregulated) compared with the control cells (Figures 1E and 1F). These mitochondria-associated DEGs in NSCs and neurons were also depicted in a volcano plot by plotting the magnitude of change against the measure of significance (Figures 1G and 1H).

Strikingly, various mitochondria-associated DEGs linked to mitochondrial electron transport chain (ETC) complexes were downregulated in WS NSCs and neurons compared with their control counterparts (Figures 1E–1H). These results were confirmed by qPCR for a set of mitochondrial ETC complex genes that were identified in either or both NSC and neuron datasets (Figures 1I and 1J). These genes include *NDUFA10* (complex I), *SDHB* (complex II), *COX16* (complex IV), and *ATP5PD* (complex V). Indeed, significant reduction in the expression of these mitochondrial ETC complex genes was found in WS1 NSCs and neurons compared with CT1 cells (Figures 1I and 1J). These data suggest that there are deficits in mitochondrial ETC complexes that could ultimately lead to mitochondrial deregulation in WS neuronal cells.

Mitochondrial dysfunction in WS hiPSC-derived neuronal cells

Since perturbations in mitochondrial ETC complexes can alter mitochondrial membrane potential ($\Delta\Psi_m$) (Zorova et al., 2018), we analyzed mitochondrial functionality in NSCs and neurons derived from multiple CT and WS hiPSC lines. Measurement of $\Delta\Psi_m$ was performed using tetramethylrhodamine ethyl ester (TMRE), a positively charged dye sequestered by negatively charged active mitochondria (Rosenstock et al., 2022). WS NSCs and neurons exhibited lower $\Delta\Psi_m$ compared with the respective CT cells (Figures 2A and 2B), indicating mitochondrial depolarization in WS cells. Since this is often associated with oxidative stress (Murphy, 2009), we assessed intracellular reactive oxygen species (ROS) levels using a cell-permeable fluorescent dye, H_2DCF-DA (Rosenstock et al., 2022). Indeed, WS NSCs and neurons had elevated ROS levels compared with their CT counterparts (Figures 2C and 2D). Likely because of mitochondrial deregulation, ATP levels were decreased in WS NSCs and neurons in comparison with the respective CT cells (Figures 2E and 2F). However, inconsistent changes were observed for mitochondrial Ca^{2+} in WS NSCs and neurons compared with their respective controls (Figures S2A and S2B), as measured by Fluo-3 AM Ca^{2+} indicator (Rosenstock et al., 2022). While WS NSCs showed a reduction in mitochondrial Ca^{2+} compared with CT NSCs despite variability between the individual WS lines, no significant difference was found between WS and CT neurons (Figures S2A and S2B). These data demonstrate increased oxidative stress and improper $\Delta\Psi_m$ in WS NSCs and neurons, and such a phenotype could be a contributing factor to neurodegeneration (Nunnari and Suomalainen, 2012).

We next analyzed mitochondrial respiration by measuring oxygen consumption rate (OCR) in two CT and three WS NSCs. The OCR levels of WS NSCs were lower than CT NSCs (Figure 2G). We evaluated key parameters of mitochondrial function by sequential addition of oligomycin, BAM15, and rotenone/antimycin A, which respectively inhibit ATP synthase, disrupt $\Delta\Psi_m$, and block mitochondrial respiration (Kenwood et al., 2014). Basal respiration and ATP production were diminished in WS NSCs compared with CT NSCs (Figures 2H and 2I), but there were no significant differences in maximal respiration or proton leak (Figures S2C and S2D). These mitochondrial respiratory defects were further seen in WS1 neurons where maximal respiration was also decreased (Figures 2J–2N). It is possible that WS being associated with neurodegeneration, the mitochondrial phenotypes could be aggravated in neurons. Moreover, decreased ATP production in WS NSCs and neurons was consistent with lower ATP levels in these cells (Figures 2E, 2F, 2I, and 2L). Overall, these data suggest mitochondrial dysfunction in WS NSCs and neurons as shown by compromised mitochondrial respiration, lower ATP production, decreased $\Delta\Psi_m$, and increased ROS.

Genetic rescue of mitochondrial phenotype by restoration of WFS1 expression

To investigate the role of WFS1 in regulating mitochondrial function, we studied whether restoration of wild-type WFS1 affected the mitochondrial phenotypes in WS neuronal cells. We used WS5R "rescued" hiPSC line wherein *WFS1* cDNA under a doxycycline (Dox) inducible promoter was introduced via CRISPR-Cas9-mediated knockin at the *AAVS1* locus in WS patient-derived WS5 hiPSC line (Pourtoy-Brasselet et al., 2021). There were no off-target effects in WS5R hiPSCs due to genome editing, as confirmed by sequencing of the top five possible off-target loci predicted by CRISPOR (Figures S3A–S3E). In this "rescued" system, WS5R hiPSC-derived cells resemble WS patient-derived (mutant) line in the absence of Dox but act as a corrected (rescued) line in the presence of Dox due to WFS1 restoration (Figure S4A). Dox treatment restored WFS1 protein level, as confirmed by immunostaining and immunoblotting, in WS5R hiPSC-derived NSCs and neurons that also expressed the cell-specific markers (Figures 3A–3C and S4B).

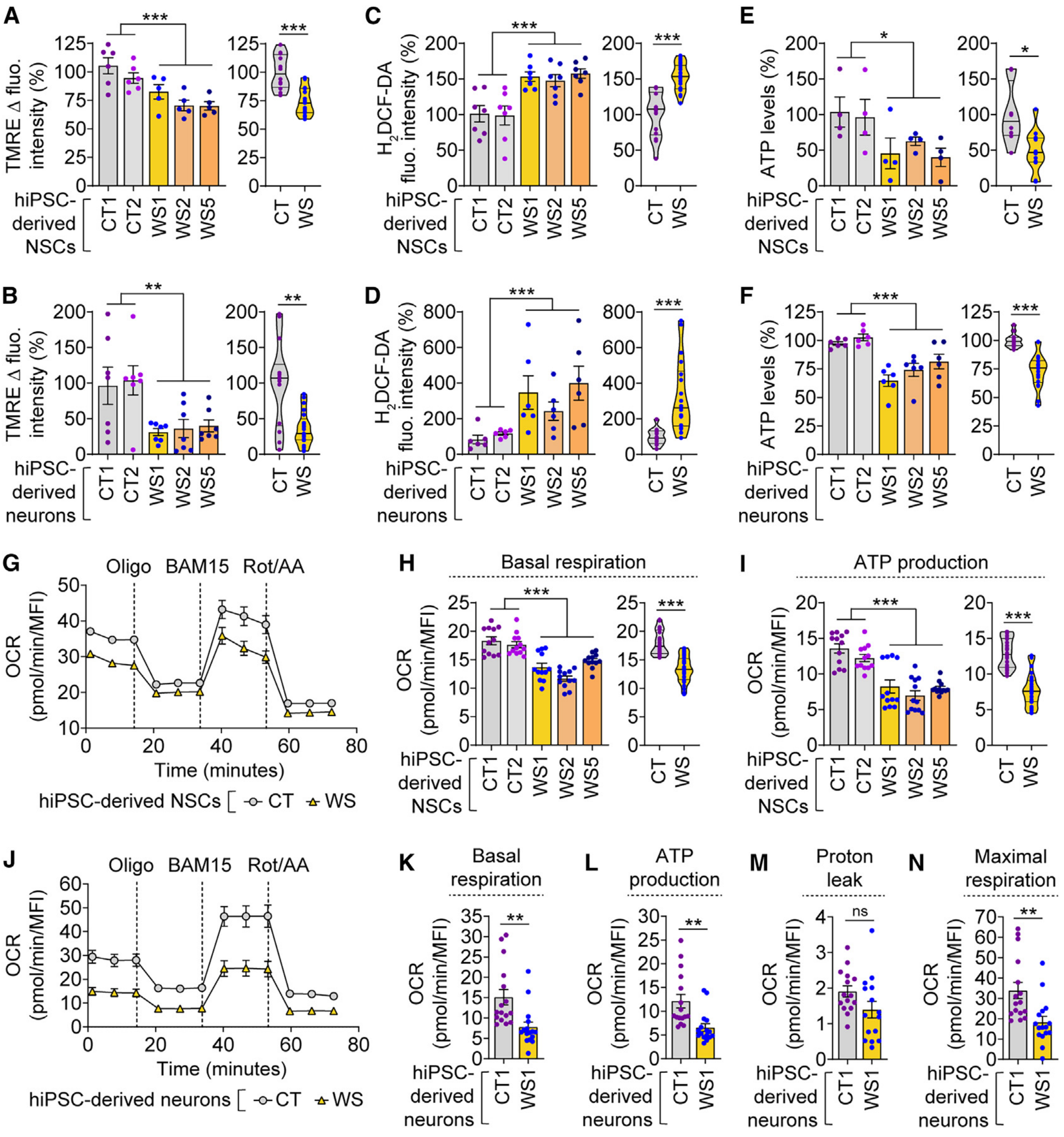

Figure 2. Mitochondrial dysfunction in WS patient hiPSC-derived NSCs and neurons

(A–F) Measurements of $\Delta\Psi m$ by TMRE Δ fluorescence intensity (A, B), ROS by H_2DCF-DA fluorescence intensity (C, D), and ATP levels (E, F) in CT1, CT2, WS1, WS2, and WS5 hiPSC-derived NSCs (A, C, E) and neurons (4 w; B, D, F).

(G–N) Oxygen consumption rate (OCR) levels (G, J) were measured post mitochondrial stress test, involving oligomycin (Oligo), BAM15, and rotenone (Rot)/antimycin A (AA) treatment, in hiPSC-derived NSCs (in CT1, CT2, WS1, WS2, and WS5; G–I) and neurons (4 w; in CT1 and WS1; J–N). Basal respiration (H, K), ATP production (I, L), proton leak (M), and maximal respiration (N) were calculated as described in experimental procedures and Table S6. MFI: mean fluorescent intensity.

Graphical data are mean ± SEM of n = 4–16 biological replicates as indicated (A–N) or displayed as violin plots (line at median) of CT and WS groups (A–F, H, I). p values were calculated by unpaired two-tailed Student's t test on two (A–F) or three (G–N) independent experiments. $^{*}p < 0.05$; $^{**}p < 0.01$; $^{***}p < 0.001$; ns, non-significant. See also Figure S2.

In the absence of Dox, WS5R NSCs and neurons (mutant condition) exhibited a reduction in $\Delta\Psi_m$ compared with the respective CT1 cells (control condition); a phenotype similar to WS1 cells (Figures 3D and 3E). However, Dox treatment (rescued condition) restored $\Delta\Psi_m$ in WS5R cells but had no significant effect in CT1 or WS1 cells (Figures 3D and 3E). This suggests that restoration of $\Delta\Psi_m$ is due to WFS1 expression and not because of WFS1-independent effects of Dox. Accordingly, increased ROS levels in WS5R cells was reduced by Dox (Figures 3F

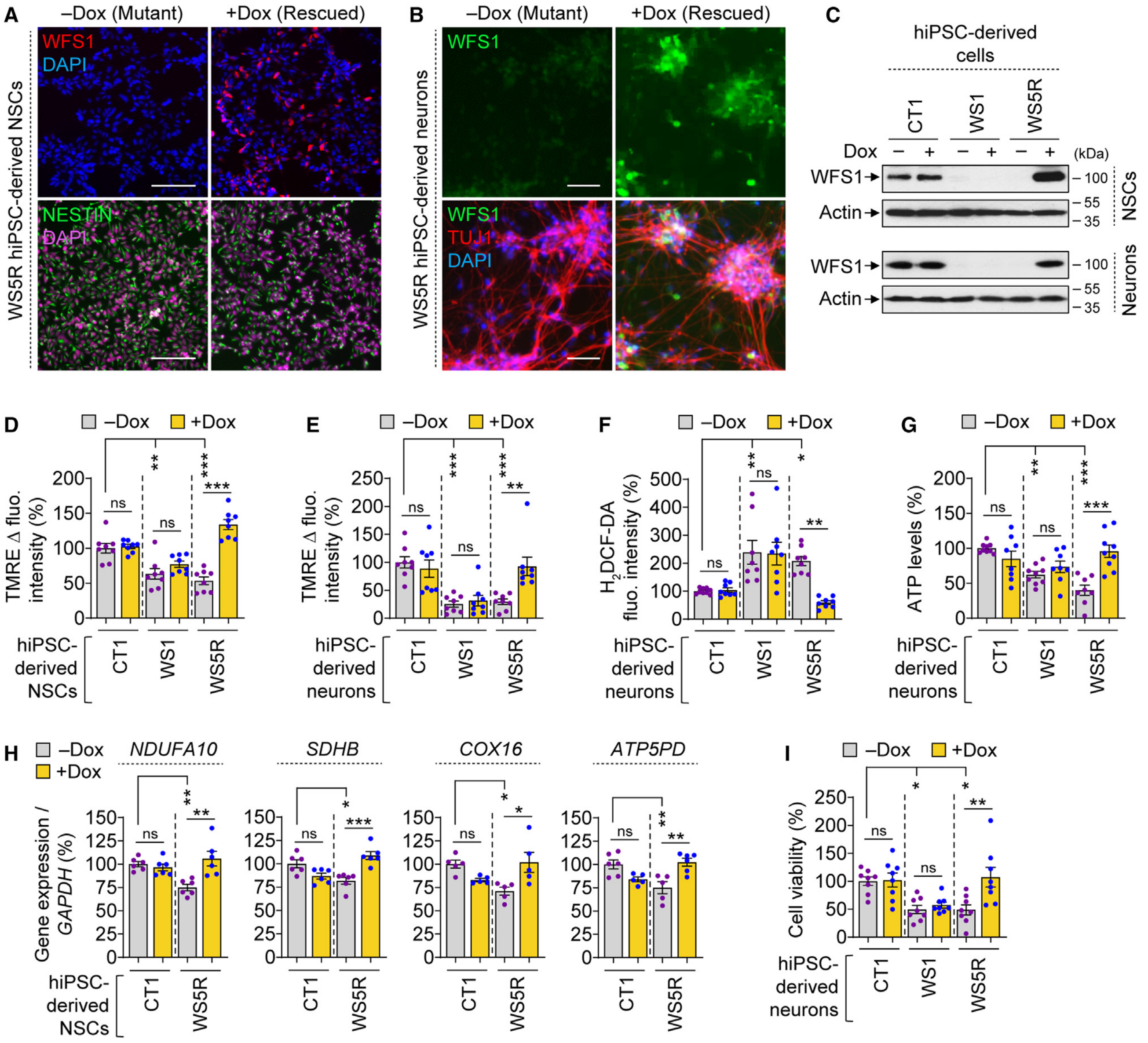

Figure 3. Genetic rescue of mitochondrial phenotypes and cell viability upon WFS1 restoration in WS patient hiPSC-derived cells

(A–C) Immunofluorescence images of WFS1 (A, B), NESTIN (A), and TUJ1 (B) and immunoblotting analysis of WFS1 (C) in CT1, WS1, and WS5R hiPSC-derived NSCs (A, C) and neurons (4 w; B, C), treated with or without 50 ng/mL doxycycline (Dox) for 48 h.

(D–I) Measurements of ΔΨm by TMRE Δ fluorescence intensity (D, E), ROS by H_2DCF-DA fluorescence intensity (F), ATP levels (G), mitochondrial gene expression (H), and cell viability (I) in CT1, WS1, and WS5R hiPSC-derived NSCs (D, H) and neurons (4 w; E–G, I), treated with or without 50 ng/mL Dox for 48 h.

Graphical data are mean ± SEM of n = 5–9 biological replicates as indicated (D–I). p values were calculated by one-way ANOVA with Tukey's multiple comparisons test on three independent experiments (D–I). $^*p < 0.05$; $^{**}p < 0.01$; $^{***}p < 0.001$; ns, non-significant. Scale bar, 50 (B) or 100 (A) μm. See also Figures S3 and S4.

and S4C), but this rescue effect was not seen in CT1 or WS1 cells (Figure 3F). Likewise, low ATP levels in WS5R cells were increased by Dox (Figures 3G and S4D), which had no significant effect in CT1 or WS1 cells (Figure 3G). Moreover, the reduction in mitochondria-associated DEG expression linked to mitochondrial ETC complexes such as *NDUFA10*, *SDHB*, *COX16*, and *ATP5PD*, as seen in WS1 NSCs compared with CT1 NSCs, was also found in WS5R NSCs in the absence of Dox (Figures 1I and 3H). Treatment with Dox restored mitochondrial

gene expression in WS5R NSCs to levels comparable to that in CT1 cells without causing any significant changes in CT1 cells (Figure 3H).

Since loss of $\Delta\Psi_m$, elevation in ROS, and reduction in ATP levels could be detrimental to the cells (Nunnari and Suomalainen, 2012), we further analyzed cell viability in WS patient-derived cells and the effects of WFS1 restoration on them. Indeed, cell viability was significantly reduced in WS1 and WS5R neurons compared with CT1 neurons in the absence of Dox (Figure 3I). Treatment with Dox, which restored WFS1 level only in WS5R neurons, improved cell viability in these cells but not in WS1 neurons lacking the transgene or in CT1 neurons (Figures 3C and 3I). Thus, Dox-induced restoration of WFS1, but not any other direct effects of Dox, is attributed to the cytoprotective effect in WS neurons. Similarly, Dox-induced WFS1 expression significantly improved cell viability in WS5R NSCs (Figures 3C and S4E). Collectively, these data suggest that restoration of WFS1 levels rescues the mitochondrial and cell death phenotypes in WS neuronal cells.

Identification of VDAC1 as WFS1 interactor and loss of this interaction in WS cells

To elucidate the potential mechanism underlying mitochondrial dysfunction in WS cells, we took an unbiased approach to identify WFS1 interactors via immunoprecipitation (IP) and liquid chromatography with tandem mass spectrometry (LC-MS/MS). Protein lysate of HEK293 cells overexpressing Myc-tagged WFS1 (Zatyka et al., 2008) was used for IP with WFS1 antibody or corresponding IgG from non-immunized animals as a negative control, followed by in-gel trypsin digestion and protein identification via LC-MS/MS (Figures 4A and S5A). Apart from identifying known WFS1 interactors like sarco(endo)plasmic reticulum Ca^{2+} ATPase 2 (SERCA; also known as AT2A2) (Zatyka et al., 2015), several new interactors were identified such as voltage-dependent anion channel (VDAC) isoforms VDAC1, VDAC2, and VDAC3, and chaperone glucose-regulated proteins (GRP) GRP75 (Figure 4A). VDACs are known to regulate mitochondrial function by acting as gatekeepers of the transport of metabolites, nucleotides, and ions (Camara et al., 2017; Shoshan-Barmatz et al., 2010). We selected VDAC1 for further analysis because it is the most abundant protein on mitochondrial outer membrane (Camara et al., 2017), essential for neurite maintenance (Paschon et al., 2019), and had the highest MOWSE (molecular weight search) score among the VDAC isoforms in LC-MS/MS analysis (Figure 4A).

WFS1-VDAC1 interaction was confirmed in HEK293 cells by co-IP (Figures 4B and 4C). VDAC1 was detected only in Myc-WFS1 overexpressing cells immunoprecipitated with WFS1 antibody but not in negative controls with IgG from non-immunized animals (Figure 4C), and also not in cells transfected with empty-Myc vector immunoprecipitated with WFS1 antibody (Figure 4B). In empty-Myc expressing HEK293 cells, low level of endogenous WFS1 was not sufficient to detect VDAC1 by co-IP (Figure 4B). We further examined WFS1-VDAC1 interaction in WS5R hiPSC-derived NSCs where WFS1 protein could be restored by Dox treatment (Figures 3C and 4D). VDAC1 was detected by co-IP only in Dox-treated WS5R NSCs immunoprecipitated with WFS1 antibody but not in negative IgG control or in cells without Dox lacking WFS1 expression (Figure 4D). The immunoblots re-probed with respective antibodies demonstrated the amount of immunoprecipitated WFS1, and the inputs indicated the levels of WFS1 and VDAC1 in the samples (Figures 4B–4D). Furthermore, WFS1 colocalized with VDAC1 in WS5R NSCs treated with Dox, but no colocalization was seen in cells without Dox that did not express WFS1 (Figure 4E). These data suggest that WFS1 interacts and colocalizes with VDAC1, which is abolished upon loss of WFS1, thereby raising the possibility that this could impact the ability of VDAC1 to regulate mitochondrial function. However, VDAC1 protein levels and gene expression were not significantly different between CT and WS NSCs and neurons (Figures S5B–S5D), suggesting that mutations in WFS1 did not influence VDAC1 turnover and stability. Moreover, WFS1 exhibited some degree of colocalization with TOM20, which is an outer mitochondrial membrane protein, in Dox-treated WS5R NSCs (Figure S5E). Although we have not detected TOM20 as an interacting partner of WFS1 via mass spectrometry, this colocalization could be likely due to the proximity and involvement of WFS1 in MAMs.

Restoration of WFS1 increases MAMs and improves mitochondrial dynamics

Proper functioning of mitochondria relies on their spatial and temporal control in cells for which the mitochondria establish contact with different organelles, such as the ER to form MAMs (Delprat et al., 2018). Both WFS1 and VDAC1 are MAM-associated proteins (Delprat et al., 2018), and loss of WFS1 in WS reduced the number of MAMs in patient fibroblasts (Angebault et al., 2018). We thus studied if WFS1 restoration in WS patient-derived neurons could influence the MAMs, which was analyzed via the colocalization between MitoTracker (mitochondrial marker) and calnexin (ER marker) (Wang et al., 2021). Remarkably, restoration of WFS1 in Dox-treated WS5R neurons (rescued) significantly increased mitochondria-ER colocalization compared with WS5R neurons without Dox (mutant) (Figures 5A and 5B). This suggests a positive correlation between MAMs and WFS1 levels, thereby hinting at the possibility that reinstating WFS1-VDAC1 interaction in rescued cells might facilitate more MAM formation and consequently improve mitochondrial function.

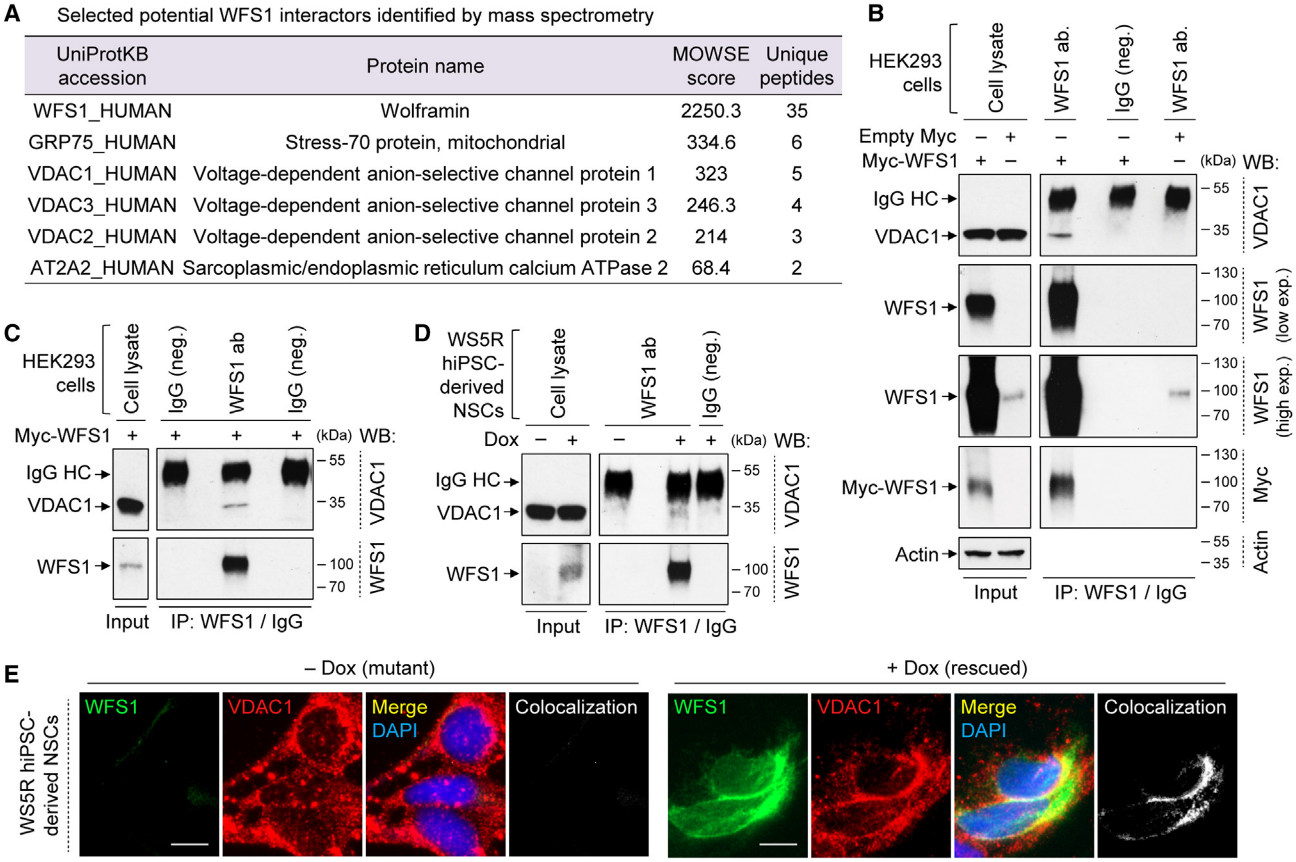

A Selected potential WFS1 interactors identified by mass spectrometry

UniProtKB accession	Protein name	MOWSE score	Unique peptides
WFS1_HUMAN	Wolframin	2250.3	35
GRP75_HUMAN	Stress-70 protein, mitochondrial	334.6	6
VDAC1_HUMAN	Voltage-dependent anion-selective channel protein 1	323	5
VDAC3_HUMAN	Voltage-dependent anion-selective channel protein 3	246.3	4
VDAC2_HUMAN	Voltage-dependent anion-selective channel protein 2	214	3
AT2A2_HUMAN	Sarcoplasmic/endoplasmic reticulum calcium ATPase 2	68.4	2

Figure 4. Identification of WFS1-VDAC1 interaction

(A) Table of selected known and novel interactors of WFS1 identified by mass spectrometry after immunoprecipitation (IP) with WFS1 antibody in HEK293 cells expressing Myc-WFS1.

(B) Immunoblotting analysis of VDAC1, WFS1, and Myc in cell lysates (input) or after IP with WFS1 antibody or IgG (negative control) in HEK293 cells, transfected with empty-Myc or Myc-WFS1. Detection of rabbit IgG heavy chain (IgG HC) with anti-mouse light chain secondary antibody was likely due to cross-reactivity. High and low exposures (exp.) of WFS1 IP and input immunoblots are shown. WB: western blot.

(C and D) Immunoblotting analyses of VDAC1 and WFS1, after IP with WFS1 antibody or IgG (negative control), in the input (cell lysate) and IP samples of HEK293 cells transfected with Myc-WFS1 (C) or of WS5R hiPSC-derived NSCs treated with or without 50 ng/mL Dox for 48 h (D).

(E) Immunofluorescence images of VDAC1 and WFS1 colocalization in WS5R hiPSC-derived NSCs, treated with or without 50 ng/mL Dox for 48 h. Scale bar, 10 μm (E). See also Figure S5.

Apart from the connection between mitochondria and ER, we also assessed mitochondrial network in our "rescued" system after MitoTracker staining (Valente et al., 2017). A moderate but significant increase in mitochondrial branch length, mitochondrial summed branch length, and mitochondrial footprint was observed upon WFS1 restoration in Dox-treated WS5R neurons, compared with WS5R neurons without Dox (Figures 5A and 5C–5F). We further analyzed some of the proteins involved in the core machinery of mitochondrial fission and fusion, which influence the dynamic nature of mitochondrial network, such as DRP1 (dynamin-related protein 1) and OPA1 (optic atrophy protein 1), respectively (Westermann, 2010). WS neurons exhibited increased DRP1 and decreased OPA1 levels compared with CT neurons (Figures 5G and 5H), raising the possibility that the mitochondria are probably undergoing more fission and less fusion in patient neurons that might contribute to shortening of the mitochondrial branch length. These data are in accordance with decreased mitochondrial length and fusion dynamics demonstrated in rat neuronal cells with *Wfs1* knockdown (Cagalinec et al., 2016). Genetic rescue by WFS1 restoration reduced DRP1 levels in Dox-treated WS5R neurons compared with WS5R neurons without Dox but had no effects on OPA1

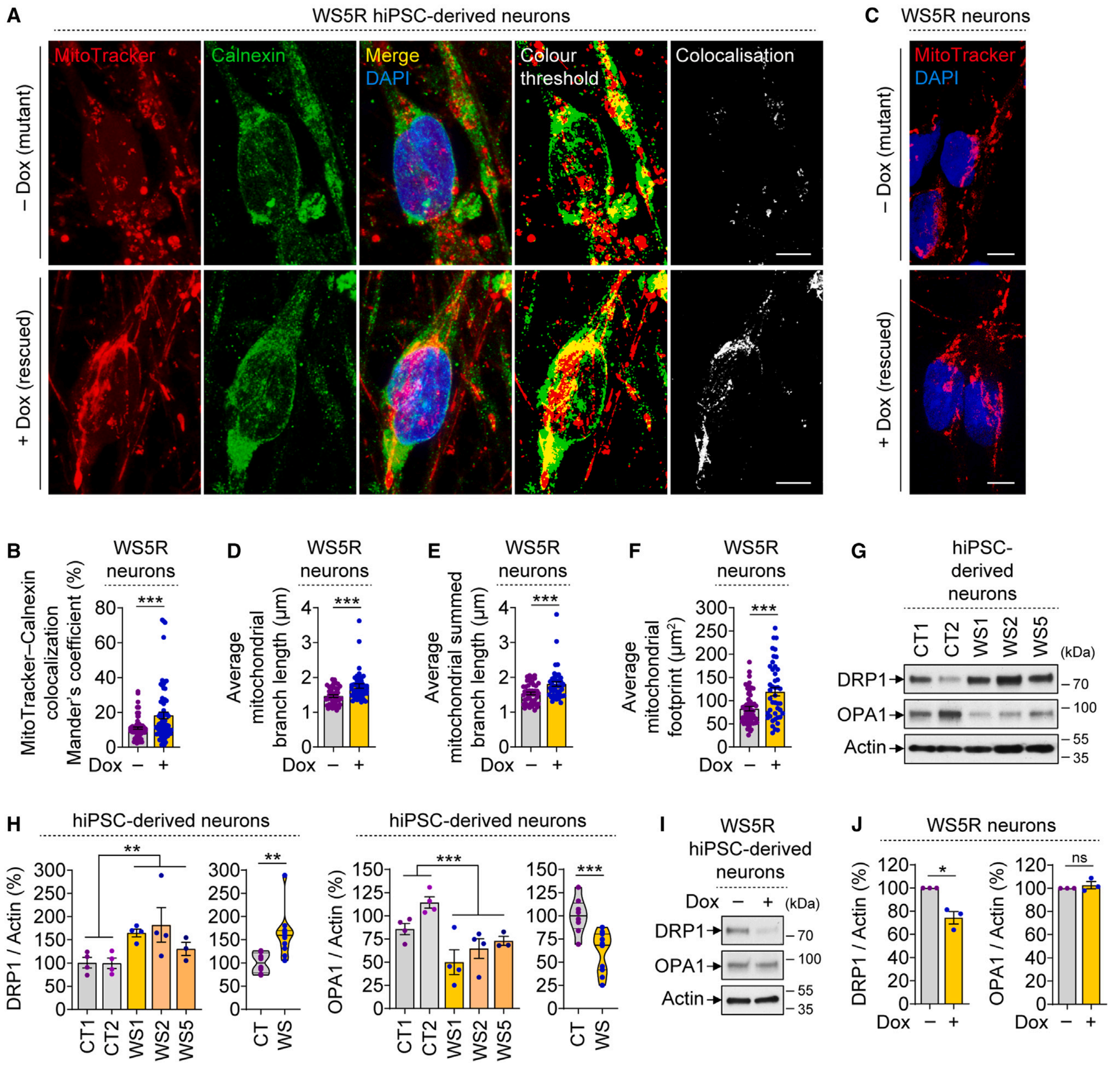

Figure 5. Increase in MAMs and mitochondrial branch length upon WFS1 restoration in WS patient hiPSC-derived cells exhibiting perturbed mitochondrial dynamics

(A–F) Immunofluorescence images of MitoTracker and Calnexin (A), Mander's coefficient of their colocalization (B), MitoTracker staining images (C), and average mitochondrial branch length (D), mitochondrial summed branch length (E), and mitochondrial footprint (F) in WS5R hiPSC-derived neurons (4 w), treated with or without 50 ng/mL Dox for 48 h.

(G–J) Immunoblotting (G, I) and densitometric (H, J) analyses of DRP1 and OPA1 in hiPSC-derived neurons (4 w) of CT1, CT2, WS1, WS2, and WS5 (G, H) or of WS5R treated with or without 50 ng/mL Dox for 48 h (I, J).

Graphical data are mean ± SEM of n = 3–4 biological replicates as indicated (H, J) or of ~75 (B) or ~50 (D–F) images per condition from n = 3 biological replicates or displayed as violin plots (line at median) of CT and WS groups (H). p values were calculated by unpaired two-tailed Student's t test on three independent experiments (B, D–F, H, J). $*p < 0.05$; $**p < 0.01$; $***p < 0.001$; ns, non-significant. Scale bar, 5 µm (A, C).

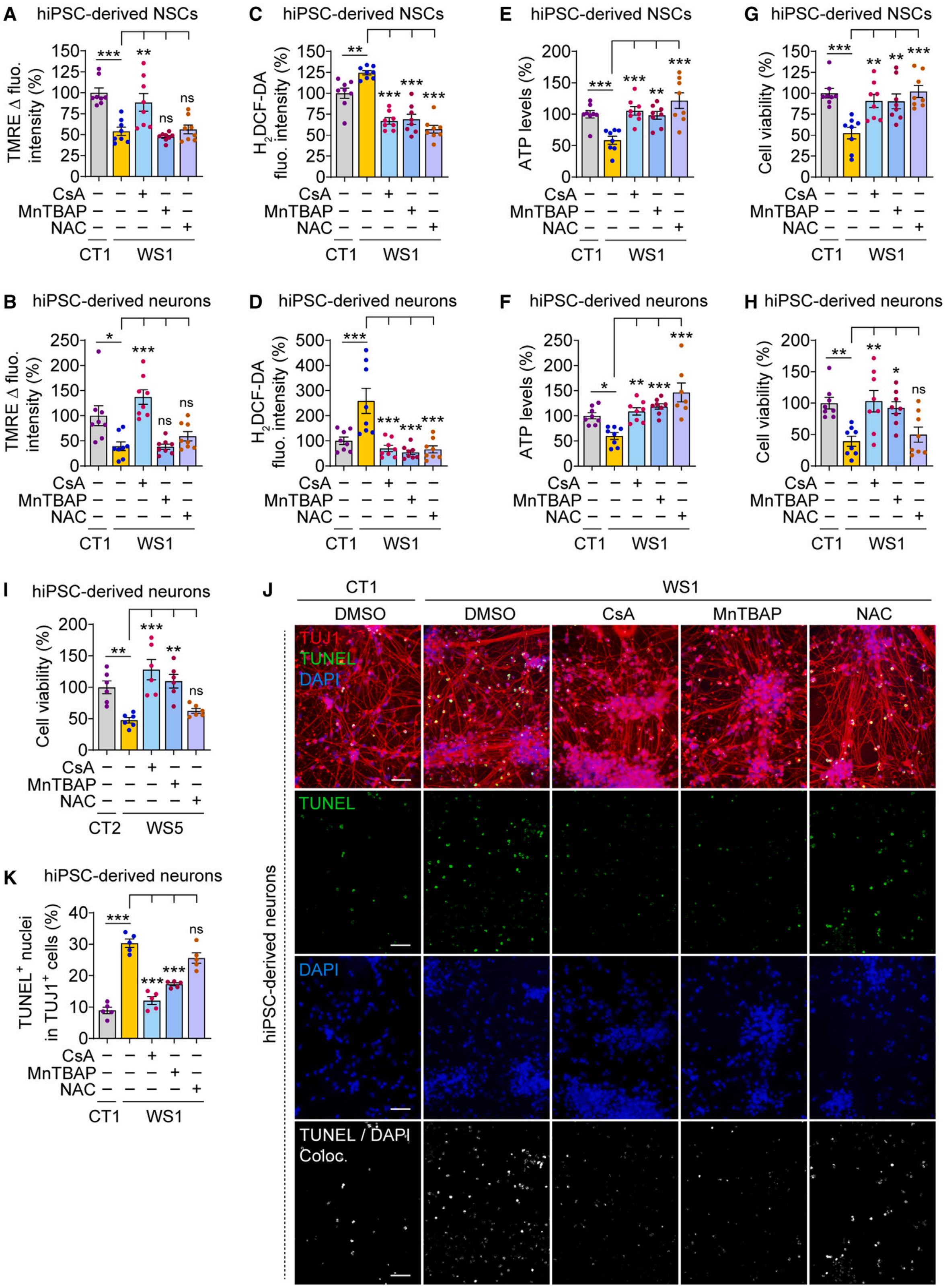

(legend on next page)

(Figures 5I and 5J). This implies that reducing the fission events could improve mitochondrial branch length in WS neurons, although the fission and fusion processes are dynamic and reciprocally regulated (Sabouny and Shutt, 2020). Overall, our data suggest that restoring WFS1 levels in WS neurons increases MAMs and mitochondrial branch length.

Pharmacological rescue of mitochondrial and cell death phenotypes

Finally, we utilized WS patient-derived neuronal platforms to evaluate the therapeutic efficacy of pharmacological agents modulating mitochondrial function. These compounds include (1) cyclosporin A (CsA), which inhibits mitochondrial permeability transition pore (PTP) and increases resting $\Delta\Psi_m$ (Cassarino et al., 1998; Halestrap et al., 1997); (2) MnTBAP, which is a superoxide dismutase mimetic and superoxide scavenger (Faulkner et al., 1994); and (3) N-acetyl cysteine (NAC), which is an anti-oxidant and a free radical scavenger (Aldini et al., 2018) (Figure S5F). Since WS NSCs and neurons exhibited mitochondrial dysfunction, we examined the effects of these compounds on both cell types. Consistent with our findings (Figures 2A–2F), WS1 NSCs and neurons displayed reduction in $\Delta\Psi_m$, elevation in ROS, and lower ATP levels compared with the CT1 counterparts (Figures 6A–6F). We found that CsA completely restored $\Delta\Psi_m$ in WS1 cells owing to its mechanism of action of inhibiting the mitochondrial PTP, whereas MnTBAP and NAC did not have any effects (Figures 6A and 6B). However, these superoxide and free radical scavengers, as well as CsA, lowered ROS levels in WS1 cells (Figures 6C and 6D). Moreover, all the compounds elevated ATP levels in WS1 cells (Figures 6E and 6F). These data suggest that mitochondrial dysfunction in WS neuronal cells can be rescued by pharmacological interventions.

We next analyzed the effects of these compounds on the viability of WS neuronal cells. Both WS1 NSCs and neurons exhibited substantial reduction in cell survival compared with CT1 cells under basal state (Figures 6G and 6H). While all the compounds rescued cell viability in WS1 NSCs (Figure 6G), only CsA and MnTBAP were effective in WS1 neurons (Figure 6H). We further evaluated the effects of these compounds in neurons differentiated from another WS patient-derived line, WS5, which also had lower cell survival compared with CT2 neurons (Figure 6I). Similar to our findings in WS1 neurons, CsA and MnTBAP improved the viability of WS5 neurons (Figure 6I). We next assessed the cytoprotective effects of these compounds via terminal deoxynucleotidyl transferase dUTP nick end labeling (TUNEL) staining of apoptotic nuclei in neurons immunostained with the neuronal marker TUJ1. In line with decreased WS neuronal viability (Figures 3I, 6H, and 6I), $TUJ1^+$ WS1 neurons had substantially higher amount of $TUNEL^+$ staining compared with $TUJ1^+$ CT1 neurons (Figures 6J and 6K), indicating higher basal cell death in WS1 neurons. CsA and MnTBAP decreased the number of $TUNEL^+$ apoptotic nuclei in $TUJ1^+$ WS1 neurons (Figures 6J and 6K). Although NAC had a tendency to lower cell death in WS neurons, its effect was not significant (Figures 6H–6K). We did not find any significant overcorrection effects of the drugs in rescuing $\Delta\Psi_m$ and cell viability (Figures 6A, 6B, and 6G–6K), but ROS was suppressed in WS cells to levels lower than CT cells (Figures 6C and 6D). Collectively, these data indicate that modulating mitochondrial function by CsA or MnTBAP is cytoprotective in WS neuronal cells.

DISCUSSION

In summary, we show a causal link between loss of WFS1 and mitochondrial dysfunction that could be rescued by genetic or pharmacological interventions (Figure 7). RNA-seq analysis revealed significant perturbations in mitochondria-associated genes in WS patient hiPSC-derived NSCs and neurons, which exhibited mitochondrial depolarization, oxidative stress, and reduction in mitochondrial respiration and ATP production. The mitochondrial and cell death phenotypes appear to be more aggravated in WS neurons than WS NSCs. Since the mitochondrial abnormalities were detrimental for WS patient-derived neuronal cells, our data are suggestive of mitochondrial dysfunction contributing to neurodegeneration (Johri and Beal, 2012; Nunnari and Suomalainen, 2012). While there are contradictory reports on mitochondrial functionality in cell models that are either non-human or not

Figure 6. Pharmacological rescue of mitochondrial and cell death phenotypes in WS patient hiPSC-derived NSCs and neurons
(A–J) Measurements of $\Delta\Psi$m by TMRE Δ fluorescence intensity (A, B), ROS by H_2DCF-DA fluorescence intensity (C, D), ATP levels (E, F) and cell viability (G–I), and immunofluorescence images of TUJ1 with TUNEL staining (J) and quantification of $TUNEL^+$ apoptotic nuclei (K), in CT1 (A–H, J, K), CT2 (I), WS1 (A–H, J, K), and WS5 (I) hiPSC-derived NSCs (A, C, E, G) and neurons (4 w; B, D, F, H–K), where WS1 or WS5 cells were treated with or without 1 μM cyclosporin A (CsA), 2 μM MnTBAP, and 100 μM N-acetyl cysteine (NAC) for 2 (in NSCs; A, C, E, G) or 6 (in neurons; B, D, F, H–K) days, respectively.
Graphical data are mean ± SEM of n = 5–8 biological replicates as indicated (A–I, K). p values were calculated by one-way ANOVA with Tukey's multiple comparisons test on three independent experiments (A–I, K). *p < 0.05; **p < 0.01; ***p < 0.001; ns, non-significant. Scale bar, 50 μm (J). See also Figure S5.

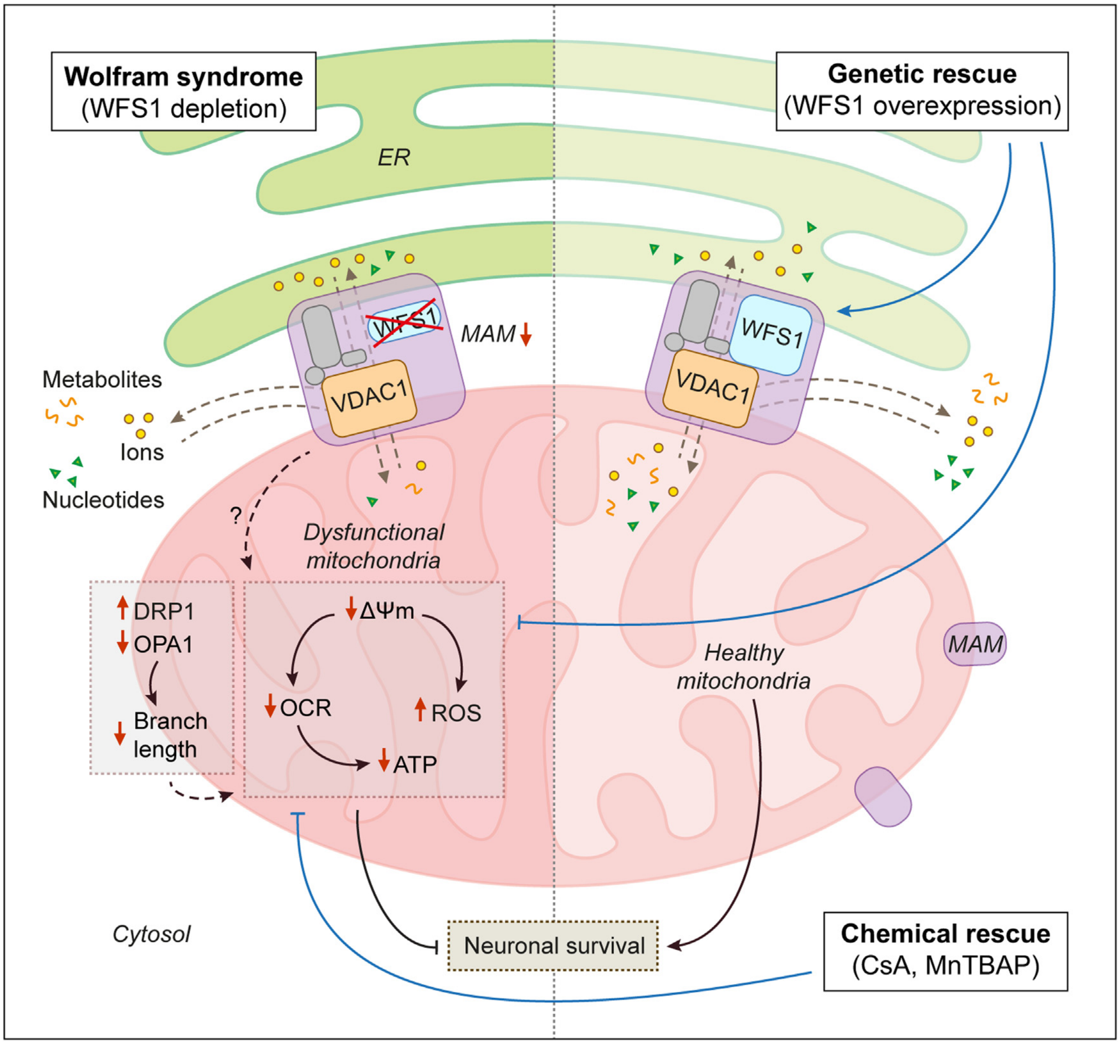

Figure 7. Schematic representation of genetic and chemical rescue of mitochondrial dysfunction in Wolfram syndrome
Depletion of WFS1 in WS (left panel) causes mitochondrial dysfunction involving mitochondrial depolarization (lower $\Delta\Psi_m$), oxidative stress (increased ROS), decreased mitochondrial respiration (less OCR), and lower ATP production, ultimately leading to neuronal cell death. Loss of WFS1-VDAC1 interaction in WS is suggested to decrease the MAMs and their function that could possibly deregulate mitochondrial dynamics (increased DRP1, decreased OPA1, and shortening of branch length) and also affect the metabolic functions of mitochondria by perturbing the transport of ions, nucleotides, and metabolites. Genetic rescue by WFS1 overexpression or chemical rescue by CsA and MnTBAP (right panel) attenuates the mitochondrial phenotypes and improves neuronal survival. WFS1 restoration in WS reinstates WFS1-VDAC1 interaction and increases MAMs and mitochondrial network that are suggested to improve mitochondrial function.

disease relevant, our data in the patient-derived disease-affected neuronal platforms are consistent with some of the phenotypes described in previous studies. These include a reduction in $\Delta\Psi_m$ in rat cortical neurons with siRNA-mediated *Wfs1* knockdown (Cagalinec et al., 2016), lower mitochondrial respiration in WS patient fibroblasts (Angebault et al., 2018), and decreased ATP level in mouse β-cell-derived MIN6 cell line with shRNA-mediated *Wfs1* knockdown (Zatyka et al., 2015). It is plausible to speculate that loss of WFS1 could indirectly affect the mRNA levels of mitochondria-associated genes via the unfolded protein response (UPR) pathway, which is an adaptive response to combat ER stress underlying WS (Fonseca et al., 2010). The UPR effectors include a range of transcription factors such as ATF4, ATF5, ATF6, and spliced XBP1, which can regulate the transcription of various proteins implicated in mitochondrial function, biogenesis, and turnover (Senft and Ronai, 2015).

Genetic rescue by restoration of WFS1 levels abrogated the mitochondrial phenotypes and improved neuronal

viability (Figure 7), implying a potential role of WSF1 in the maintenance of mitochondrial function that is attributable to neuronal survival. One possible mechanism could be via its interaction with VDAC1, which we identified as a WFS1 interactor by mass spectrometry. VDAC1 forms a complex with IP_3R on the ER through the molecular chaperone GRP75 (Szabadkai et al., 2006), and the WFS1-VDAC1 interaction is likely a part of this complex tethering ER and mitochondria because both these proteins are associated with MAMs (Delprat et al., 2018). Restoring WFS1 levels in WS cells reinstated WFS1-VDAC1 interaction and increased MAMs. We thus speculate that this interaction could facilitate the close contact between ER and mitochondrial outer membrane required for MAM formation and function. Concomitantly, restoration of WFS1 in WS cells increased mitochondrial branch length that correlated with reduction in the mitochondrial fission protein DRP1. Taken together, we further speculate that improved mitochondrial dynamics coupled with more MAMs could positively impact on mitochondrial function. Conversely, impairment in MAMs and mitochondrial dynamics are implicated in neurodegenerative diseases (Delprat et al., 2018; Westermann, 2010), and their dysfunction demonstrated in WS is possibly disrupting mitochondrial function (Angebault et al., 2018; Cagalinec et al., 2016).

Moreover, VDAC1 is a multifunctional channel of mitochondrial outer membrane that is essential for the metabolic functions of mitochondria by controlling the transport of ions, nucleotides, and metabolites (Camara et al., 2017; Shoshan-Barmatz et al., 2010). The IP_3R-GRP75-VDAC1 complex on the ER allows Ca^{2+} transfer from the ER to mitochondria (Szabadkai et al., 2006). VDAC1 also mediates metabolic flow in the opposite direction, creating ATP microdomain close to the ER and SERCA (Camara et al., 2017; Shoshan-Barmatz et al., 2010). Neurodegeneration-associated proteins, such as DJ-1 in early-onset PD and WFS1 in WS, interact with the IP_3R-GRP75-VDAC1 multicomplex to influence mitochondrial and MAM functions (Angebault et al., 2018; Liu et al., 2019). In the context of WS, WFS1 also forms a complex with IP_3R and NCS1 to promote Ca^{2+} transfer from ER to mitochondria (Angebault et al., 2018). Identification of VDAC1 as a WFS1 interactor strengthens the finding that WFS1 is a part of the IP_3R-GRP75-VDAC1 multicomplex, and interestingly, we also identified VDAC2, VDAC3, and GRP75 among the potential WFS1 interactors via mass spectrometry. Furthermore, it has been hypothesized that WFS1 could control the oligomerization of Na^+ pump subunits (Zatyka et al., 2008), and hence it might also govern VDAC1 oligomerization state and channel function. From these findings, it is plausible that loss of WFS1-VDAC1 interaction observed in WS patient-derived cells lacking WFS1 could be a potential mechanism via which mutant WFS1 might affect VDAC1 and mitochondrial functionality (Figure 7). However, we did not observe any overt perturbations in mitochondrial Ca^{2+} in WS patient-derived neuronal cells, which also displayed variability among the WS cell lines. Further work is warranted in disease-relevant cellular platforms to elucidate how mutant WFS1 could disrupt VDAC1 function or how loss of WFS1-VDAC1 interaction leads to mitochondrial dysfunction.

Of biomedical relevance, pharmacological modulation of mitochondrial function via inhibition of mitochondrial PTP by CsA or suppression of superoxide by MnTBAP, which respectively restored $\Delta\Psi_m$ and lowered oxidative stress, recovered the energy status and viability of WS NSCs and neurons (Figure 7). However, the effects of the anti-oxidant NAC on neuronal viability were not significant, which could be related to its side effects like autophagy inhibition that can augment neurodegeneration (Underwood et al., 2010). Previous studies have shown CsA to be protective in AD cell models and in rat heart after ischemia/reperfusion injury by restoring $\Delta\Psi_m$ (Cassarino et al., 1998; Halestrap et al., 1997), while MnTBAP was protective against renal injury and obesity-induced cardiac dysfunction by antagonizing oxidative stress (Bi et al., 2018; Ilkun et al., 2015; Yu et al., 2016). We found that these pharmacological agents were also cytoprotective in the context of WS patient-derived neuronal cells. Our data highlight a potential therapeutic intervention for WS that could be further examined for generalizability in related rare or common neurodegenerative diseases associated with mitochondrial defects.

EXPERIMENTAL PROCEDURES

Detailed methodologies can be found in supplemental experimental procedures in the supplemental information.

Resource availability

Corresponding author

Sovan Sarkar (s.sarkar@bham.ac.uk).

Materials availability

The hiPSC-derived NSCs were generated by L. Aubry (I-STEM, France) and obtained by S.S. and T.B. under a materials transfer agreement (MTA 1390060). The WIBR3 hESC line was obtained by S.S. from R. Jaenisch (Whitehead Institute for Biomedical Research, USA) under a materials transfer agreement (UBMTA 15–0593).

Data and code availability

The GEO accession number of the RNA-seq data is GSE156911. Mass spectrometry data for WFS1 interactors are available in MassIVE repository (MassIVE: MSV000091646). Data of the results are presented in the main paper and the supplemental information. This paper does not report any original code.

hiPSC lines and neuronal differentiation

The NSCs and neurons were generated from previously established control (CT1, CT2), WS patient-derived (WS1, WS2, WS5), and rescued (WS5R) hiPSC lines (Pourtoy-Brasselet et al., 2021; Shang et al., 2014). The hiPSCs were cultured and differentiated into NSCs and then into neurons as described previously (Boissart et al., 2013; Pourtoy-Brasselet et al., 2021). Neuronal differentiation was done for 4 weeks; neurons were cortical in nature.

RNA-seq data and gene expression analysis

AmpliSeq data (GEO: GSE156911) used in this study were previously published (Pourtoy-Brasselet et al., 2021). Commonly expressed upregulated and downregulated DEGs (p value $\leq$ 5%; fold change $\geq$ 1.5; minimum reads > 100) between mitochondria gene set of control and WS NSCs or neurons were selected using Venny diagram (v2.1.0). Mitochondria-associated DEGs were depicted in a volcano plot by plotting the magnitude of change [Log_2(Fold change)] against the measure of significance [Log_{10}(*P* adjusted)]. Gene expression was analyzed by quantitative real-time PCR (qPCR) using gene-specific primers, qPCR data calculated by $2^{-\Delta\Delta Ct}$ method, normalized to *GAPDH* expression, and calculated as percentage of control condition.

Identification of WFS1 interactors by mass spectrometry

HEK293 cell lysate overexpressing Myc-WFS1 was used for IP with WFS1 antibody or IgG from non-immunized animals using Dynabeads Protein A Immunoprecipitation Kit (Invitrogen). Co-IP proteins were separated by SDS-PAGE followed by in-gel tryptic digestion. The resulting peptides were analyzed by LC-MS/MS using Bruker Impact Q-ToF Mass Spectrometer (Bruker Daltonics). Peptides were identified using MASCOT to search the SWISSPROT human database, and protein identifications were filtered using a 1% false discovery rate and a requirement for $\geq$2 peptides using ProteinScape software (Bruker Daltonics). Mass spectrometry data for WFS1 interactors have been deposited in MassIVE repository (MassIVE: MSV000091646).

Mitochondrial $\Delta\Psi_m$, ROS, Ca^{2+}, and ATP measurements

Measurements of $\Delta\Psi_m$, ROS, mitochondrial Ca^{2+}, and ATP were respectively done using 500 nM TMRE, 20 μM CM-H_2DCF-DA, 10 μM Fluo-3 AM (Invitrogen), and ApoSENSOR ADP/ATP Ratio Bioluminescent Assay Kit (BioVision) as described previously (Rosenstock et al., 2022) or per manufacturer's protocol. Data were normalized to protein concentration by Bradford Protein Assay (Bio-Rad) and expressed as percentage of control condition.

Mitochondrial respiration measurement

Basal levels of OCRs were measured on XFe96 Extracellular Flux Analyzer (Agilent) after stimulation with 2 μM oligomycin, 3 μM BAM15, and 1 μM rotenone/antimycin A (Sigma-Aldrich) per XF Cell Mito Stress Test Kit (Agilent). Basal respiration, ATP production, proton leak, and maximal respiration were calculated, and cell number was normalized by CyQUANT Direct Cell Proliferation Assay (Invitrogen).

Measurements of MAMs and mitochondrial branch length

Analyses of MAMs (Wang et al., 2021) was done by Mander's coefficient of colocalization between MitoTracker Red CMXRos (Invitrogen) and Calnexin (~75 images per sample). Measurements of mitochondrial branch length and footprint (Valente et al., 2017) were done using Mitochondrial Network Analysis (MiNA) toolset in Fiji v2.9.0 (ImageJ2) after MitoTracker Red CMXRos staining (~50 images per sample).

Measurements of cell viability and apoptotic cells

Cell viability was measured by luminescence-based CytoTox-Glo Cytotoxicity Assay (Promega) per manufacturer's protocol. Data were expressed as percentage of control condition. Apoptotic cells were determined by Click-iT Plus TUNEL Assay for *in situ* apoptosis detection, Alexa Fluor 488 dye (Thermo Fisher Scientific) followed by immunostaining with TUJ1 antibody. The percentage of $TUNEL^+$ apoptotic nuclei was calculated from all the $TUJ1^+$ cells analyzed (200–300 cells per sample).

Statistical analysis

Graphical data are from $\geq$3 biological replicates from independent experiments and depicted as column graph scatter dot plot (mean ± SEM) or violin plot (line at median) using Prism v8.3.1 (GraphPad). Statistical significance (p value) was determined by unpaired two-tailed Student's t test or by one-way ANOVA with Tukey's or Dunnett's multiple comparisons test using Prism v8.3.1 (GraphPad). *$p < 0.05$; **$p < 0.01$; ***$p < 0.001$; ns: non-significant.

SUPPLEMENTAL INFORMATION

Supplemental information can be found online at https://doi.org/10.1016/j.stemcr.2023.04.002.

AUTHOR CONTRIBUTIONS

M.Z., T.R.R., C.S., A.M.P., G.W.H., S.L.R., D.A., A.D.M., A.S., M.E.K., V.S., G.Z., M.R., S.P.B., K.W., T.V., M.J., H.P., J.C., M.H., D.G.W., L.A., and S.S. performed experiments, provided tools/methodologies, and/or analyzed data; S.S., T.B., T.R.R., L.A., M.E.K., and E.M.F. acquired funding; S.S., T.R.R., and M.Z. conceptualized the project; S.S. administered the project, prepared figures, and wrote the manuscript with inputs from M.Z., T.R.R., L.A., and T.B.; all authors contributed to and/or approved the final manuscript.

ACKNOWLEDGMENTS

This study was supported by LifeArc Philanthropic Fund (P2019-0004), LifeArc Pathfinder Award, along with Wellcome Trust Seed Award (109626/Z/15/Z), Wellcome Trust ISSF (1516ISSFFEL10), UKIERI DST Thematic Partnership Award (2016-17-0087), and Birmingham Fellowship to S.S., University of Birmingham (UoB) Brazil Visiting Fellowship, FAPESP-UoB Strategic Collaboration Fund, and Rutherford Fellowship to S.S. and T.R.R., BBSRC and UoB-funded MIBTP Studentship (BB/T00746X/1) to M.E.K. and S.S., FAPESP grant (2015/02041-1) to T.R.R., Wellcome Trust Senior Research Fellowship (217202/Z/19/Z) to E.M.F., Agence Nationale de la Recherche: Labex REVIVE (ANR-10-LABX-73) to L.A., and

MRC DPFS (MR/P007732/1) to T.B. We thank New York Stem Cell Foundation for providing WS1 and WS2 hiPSC lines, R. Jaenisch for providing WIBR3 hESC line, J. Barlow and UoB Mitochondrial Profiling Center for technical support and the use of Seahorse facility, and M. Coleman for the use of microplate reader. T.R.R. is also a Volunteer Professor at University of São Paulo, Brazil. T.B. is an NIHR Senior Investigator. S.S. is also Former Fellow for life at Hughes Hall, University of Cambridge, UK.

CONFLICT OF INTERESTS

The authors declare no competing interests.

Received: July 26, 2022
Revised: April 5, 2023
Accepted: April 6, 2023
Published: May 9, 2023

REFERENCES

Abreu, D., and Urano, F. (2019). Current landscape of treatments for Wolfram syndrome. Trends Pharmacol. Sci. *40*, 711–714. https://doi.org/10.1016/j.tips.2019.07.011.

Aldini, G., Altomare, A., Baron, G., Vistoli, G., Carini, M., Borsani, L., and Sergio, F. (2018). N-Acetylcysteine as an antioxidant and disulphide breaking agent: the reasons why. Free Radic. Res. *52*, 751–762. https://doi.org/10.1080/10715762.2018.1468564.

Angebault, C., Fauconnier, J., Patergnani, S., Rieusset, J., Danese, A., Affortit, C.A., Jagodzinska, J., Mégy, C., Quiles, M., Cazevieille, C., et al. (2018). ER-mitochondria cross-talk is regulated by the Ca(2+) sensor NCS1 and is impaired in Wolfram syndrome. Sci. Signal. *11*, eaaq1380. https://doi.org/10.1126/scisignal.aaq1380.

Avior, Y., Sagi, I., and Benvenisty, N. (2016). Pluripotent stem cells in disease modelling and drug discovery. Nat. Rev. Mol. Cell Biol. *17*, 170–182. https://doi.org/10.1038/nrm.2015.27.

Barrett, T.G., Bundey, S.E., and Macleod, A.F. (1995). Neurodegeneration and diabetes: UK nationwide study of Wolfram (DIDMOAD) syndrome. Lancet *346*, 1458–1463. https://doi.org/10.1016/s0140-6736(95)92473-6.

Barrientos, A., Volpini, V., Casademont, J., Genís, D., Manzanares, J.M., Ferrer, I., Corral, J., Cardellach, F., Urbano-Márquez, A., Estivill, X., and Nunes, V. (1996). A nuclear defect in the 4p16 region predisposes to multiple mitochondrial DNA deletions in families with Wolfram syndrome. J. Clin. Invest. *97*, 1570–1576. https://doi.org/10.1172/JCI118581.

Bi, X., Wang, J., Liu, Y., Wang, Y., and Ding, W. (2018). MnTBAP treatment ameliorates aldosterone-induced renal injury by regulating mitochondrial dysfunction and NLRP3 inflammasome signalling. Am. J. Transl. Res. *10*, 3504–3513.

Boissart, C., Poulet, A., Georges, P., Darville, H., Julita, E., Delorme, R., Bourgeron, T., Peschanski, M., and Benchoua, A. (2013). Differentiation from human pluripotent stem cells of cortical neurons of the superficial layers amenable to psychiatric disease modeling and high-throughput drug screening. Transl. Psychiatry *3*, e294. https://doi.org/10.1038/tp.2013.71.

Bu, X., and Rotter, J.I. (1993). Wolfram syndrome: a mitochondrial-mediated disorder? Lancet *342*, 598–600. https://doi.org/10.1016/0140-6736(93)91416-j.

Cagalinec, M., Liiv, M., Hodurova, Z., Hickey, M.A., Vaarmann, A., Mandel, M., Zeb, A., Choubey, V., Kuum, M., Safiulina, D., et al. (2016). Role of mitochondrial dynamics in neuronal development: mechanism for Wolfram Syndrome. PLoS Biol. *14*, e1002511. https://doi.org/10.1371/journal.pbio.1002511.

Camara, A.K.S., Zhou, Y., Wen, P.C., Tajkhorshid, E., and Kwok, W.M. (2017). Mitochondrial VDAC1: a key gatekeeper as potential therapeutic target. Front. Physiol. *8*, 460. https://doi.org/10.3389/fphys.2017.00460.

Cassarino, D.S., Swerdlow, R.H., Parks, J.K., Parker, W.D., Jr., and Bennett, J.P., Jr. (1998). Cyclosporin A increases resting mitochondrial membrane potential in SY5Y cells and reverses the depressed mitochondrial membrane potential of Alzheimer's disease cybrids. Biochem. Biophys. Res. Commun. *248*, 168–173. https://doi.org/10.1006/bbrc.1998.8866.

Chen, Y.F., Kao, C.H., Chen, Y.T., Wang, C.H., Wu, C.Y., Tsai, C.Y., Liu, F.C., Yang, C.W., Wei, Y.H., Hsu, M.T., et al. (2009). Cisd2 deficiency drives premature aging and causes mitochondria-mediated defects in mice. Genes Dev. *23*, 1183–1194. https://doi.org/10.1101/gad.1779509.

Delprat, B., Maurice, T., and Delettre, C. (2018). Wolfram syndrome: MAMs' connection? Cell Death Dis. *9*, 364. https://doi.org/10.1038/s41419-018-0406-3.

Ehrlich, M., Ivask, M., Raasmaja, A., and Kõks, S. (2016). Analysis of metabolic effects of menthol on WFS1-deficient mice. Physiol. Rep. *4*, e12660. https://doi.org/10.14814/phy2.12660.

Eimre, M., Paju, K., Peet, N., Kadaja, L., Tarrend, M., Kasvandik, S., Seppet, J., Ivask, M., Orlova, E., and Kõks, S. (2018). Increased mitochondrial protein levels and bioenergetics in the *musculus rectus femoris* of *Wfs1*-deficient mice. Oxid. Med. Cell. Longev. *2018*, 3175313. https://doi.org/10.1155/2018/3175313.

Faulkner, K.M., Liochev, S.I., and Fridovich, I. (1994). Stable Mn(III) porphyrins mimic superoxide dismutase in vitro and substitute for it in vivo. J. Biol. Chem. *269*, 23471–23476.

Fonseca, S.G., Ishigaki, S., Oslowski, C.M., Lu, S., Lipson, K.L., Ghosh, R., Hayashi, E., Ishihara, H., Oka, Y., Permutt, M.A., and Urano, F. (2010). Wolfram syndrome 1 gene negatively regulates ER stress signaling in rodent and human cells. J. Clin. Invest. *120*, 744–755. https://doi.org/10.1172/JCI39678.

Giorgi, C., Missiroli, S., Patergnani, S., Duszynski, J., Wieckowski, M.R., and Pinton, P. (2015). Mitochondria-associated membranes: composition, molecular mechanisms, and physiopathological implications. Antioxid. Redox Signal. *22*, 995–1019. https://doi.org/10.1089/ars.2014.6223.

Guo, X., Shen, S., Song, S., He, S., Cui, Y., Xing, G., Wang, J., Yin, Y., Fan, L., He, F., and Zhang, L. (2011). The E3 ligase Smurf1 regulates Wolfram syndrome protein stability at the endoplasmic reticulum. J. Biol. Chem. *286*, 18037–18047. https://doi.org/10.1074/jbc.M111.225615.

Halestrap, A.P., Connern, C.P., Griffiths, E.J., and Kerr, P.M. (1997). Cyclosporin A binding to mitochondrial cyclophilin inhibits the

permeability transition pore and protects hearts from ischaemia/reperfusion injury. Mol. Cell. Biochem. *174*, 167–172.

Horner, S.M., Wilkins, C., Badil, S., Iskarpatyoti, J., and Gale, M., Jr. (2015). Proteomic analysis of mitochondrial-associated ER membranes (MAM) during RNA virus infection reveals dynamic changes in protein and organelle trafficking. PLoS One *10*, e0117963. https://doi.org/10.1371/journal.pone.0117963.

Ilkun, O., Wilde, N., Tuinei, J., Pires, K.M.P., Zhu, Y., Bugger, H., Soto, J., Wayment, B., Olsen, C., Litwin, S.E., and Abel, E.D. (2015). Antioxidant treatment normalizes mitochondrial energetics and myocardial insulin sensitivity independently of changes in systemic metabolic homeostasis in a mouse model of the metabolic syndrome. J. Mol. Cell. Cardiol. *85*, 104–116. https://doi.org/10.1016/j.yjmcc.2015.05.012.

Inoue, H., Tanizawa, Y., Wasson, J., Behn, P., Kalidas, K., Bernal-Mizrachi, E., Mueckler, M., Marshall, H., Donis-Keller, H., Crock, P., et al. (1998). A gene encoding a transmembrane protein is mutated in patients with diabetes mellitus and optic atrophy (Wolfram syndrome). Nat. Genet. *20*, 143–148. https://doi.org/10.1038/2441.

Johri, A., and Beal, M.F. (2012). Mitochondrial dysfunction in neurodegenerative diseases. J. Pharmacol. Exp. Ther. *342*, 619–630. https://doi.org/10.1124/jpet.112.192138.

Kenwood, B.M., Weaver, J.L., Bajwa, A., Poon, I.K., Byrne, F.L., Murrow, B.A., Calderone, J.A., Huang, L., Divakaruni, A.S., Tomsig, J.L., et al. (2014). Identification of a novel mitochondrial uncoupler that does not depolarize the plasma membrane. Mol. Metab. *3*, 114–123. https://doi.org/10.1016/j.molmet.2013.11.005.

Kõks, S., Overall, R.W., Ivask, M., Soomets, U., Guha, M., Vasar, E., Fernandes, C., and Schalkwyk, L.C. (2013). Silencing of the WFS1 gene in HEK cells induces pathways related to neurodegeneration and mitochondrial damage. Physiol. Genomics *45*, 182–190. https://doi.org/10.1152/physiolgenomics.00122.2012.

La Morgia, C., Maresca, A., Amore, G., Gramegna, L.L., Carbonelli, M., Scimonelli, E., Danese, A., Patergnani, S., Caporali, L., Tagliavini, F., et al. (2020). Calcium mishandling in absence of primary mitochondrial dysfunction drives cellular pathology in Wolfram Syndrome. Sci. Rep. *10*, 4785. https://doi.org/10.1038/s41598-020-61735-3.

Liu, Y., Ma, X., Fujioka, H., Liu, J., Chen, S., and Zhu, X. (2019). DJ-1 regulates the integrity and function of ER-mitochondria association through interaction with IP3R3-Grp75-VDAC1. Proc. Natl. Acad. Sci. USA *116*, 25322–25328. https://doi.org/10.1073/pnas.1906565116.

Murphy, M.P. (2009). How mitochondria produce reactive oxygen species. Biochem. J. *417*, 1–13. https://doi.org/10.1042/BJ20081386.

Nunnari, J., and Suomalainen, A. (2012). Mitochondria: in sickness and in health. Cell *148*, 1145–1159. https://doi.org/10.1016/j.cell.2012.02.035.

Paillusson, S., Stoica, R., Gomez-Suaga, P., Lau, D.H.W., Mueller, S., Miller, T., and Miller, C.C.J. (2016). There's something wrong with my MAM; the ER-mitochondria axis and neurodegenerative diseases. Trends Neurosci. *39*, 146–157. https://doi.org/10.1016/j.tins.2016.01.008.

Paschon, V., Morena, B.C., Correia, F.F., Beltrame, G.R., Dos Santos, G.B., Cristante, A.F., and Kihara, A.H. (2019). VDAC1 is essential for neurite maintenance and the inhibition of its oligomerization protects spinal cord from demyelination and facilitates locomotor function recovery after spinal cord injury. Sci. Rep. *9*, 14063. https://doi.org/10.1038/s41598-019-50506-4.

Poston, C.N., Krishnan, S.C., and Bazemore-Walker, C.R. (2013). In-depth proteomic analysis of mammalian mitochondria-associated membranes (MAM). J. Proteomics *79*, 219–230. https://doi.org/10.1016/j.jprot.2012.12.018.

Pourtoy-Brasselet, S., Sciauvaud, A., Boza-Moran, M.G., Cailleret, M., Jarrige, M., Polvèche, H., Polentes, J., Chevet, E., Martinat, C., Peschanski, M., and Aubry, L. (2021). Human iPSC-derived neurons reveal early developmental alteration of neurite outgrowth in the late-occurring neurodegenerative Wolfram syndrome. Am. J. Hum. Genet. *108*, 2171–2185. https://doi.org/10.1016/j.ajhg.2021.10.001.

Rigoli, L., Bramanti, P., Di Bella, C., and De Luca, F. (2018). Genetic and clinical aspects of Wolfram syndrome 1, a severe neurodegenerative disease. Pediatr. Res. *83*, 921–929. https://doi.org/10.1038/pr.2018.17.

Rosenstock, T.R., Sun, C., Hughes, G.W., Winter, K., and Sarkar, S. (2022). Analysis of mitochondrial dysfunction by microplate reader in hiPSC-derived neuronal cell models of neurodegenerative disorders. Methods Mol. Biol. *2549*, 1–21. https://doi.org/10.1007/7651_2021_451.

Sabouny, R., and Shutt, T.E. (2020). Reciprocal regulation of mitochondrial fission and fusion. Trends Biochem. Sci. *45*, 564–577. https://doi.org/10.1016/j.tibs.2020.03.009.

Senft, D., and Ronai, Z.A. (2015). UPR, autophagy, and mitochondria crosstalk underlies the ER stress response. Trends Biochem. Sci. *40*, 141–148. https://doi.org/10.1016/j.tibs.2015.01.002.

Shang, L., Hua, H., Foo, K., Martinez, H., Watanabe, K., Zimmer, M., Kahler, D.J., Freeby, M., Chung, W., LeDuc, C., et al. (2014). beta-cell dysfunction due to increased ER stress in a stem cell model of Wolfram syndrome. Diabetes *63*, 923–933. https://doi.org/10.2337/db13-0717.

Shoshan-Barmatz, V., De Pinto, V., Zweckstetter, M., Raviv, Z., Keinan, N., and Arbel, N. (2010). VDAC, a multi-functional mitochondrial protein regulating cell life and death. Mol. Aspects Med. *31*, 227–285. https://doi.org/10.1016/j.mam.2010.03.002.

Szabadkai, G., Bianchi, K., Várnai, P., De Stefani, D., Wieckowski, M.R., Cavagna, D., Nagy, A.I., Balla, T., and Rizzuto, R. (2006). Chaperone-mediated coupling of endoplasmic reticulum and mitochondrial Ca2+ channels. J. Cell Biol. *175*, 901–911. https://doi.org/10.1083/jcb.200608073.

Underwood, B.R., Imarisio, S., Fleming, A., Rose, C., Krishna, G., Heard, P., Quick, M., Korolchuk, V.I., Renna, M., Sarkar, S., et al. (2010). Antioxidants can inhibit basal autophagy and enhance neurodegeneration in models of polyglutamine disease. Hum. Mol. Genet. *19*, 3413–3429. https://doi.org/10.1093/hmg/ddq253.

Valente, A.J., Maddalena, L.A., Robb, E.L., Moradi, F., and Stuart, J.A. (2017). A simple ImageJ macro tool for analyzing mitochondrial network morphology in mammalian cell culture. Acta

Histochem. *119*, 315–326. https://doi.org/10.1016/j.acthis.2017.03.001.

Wang, C., Dai, X., Wu, S., Xu, W., Song, P., and Huang, K. (2021). FUNDC1-dependent mitochondria-associated endoplasmic reticulum membranes are involved in angiogenesis and neoangiogenesis. Nat. Commun. *12*, 2616. https://doi.org/10.1038/s41467-021-22771-3.

Westermann, B. (2010). Mitochondrial fusion and fission in cell life and death. Nat. Rev. Mol. Cell Biol. *11*, 872–884. https://doi.org/10.1038/nrm3013.

Wiley, S.E., Andreyev, A.Y., Divakaruni, A.S., Karisch, R., Perkins, G., Wall, E.A., van der Geer, P., Chen, Y.F., Tsai, T.F., Simon, M.I., et al. (2013). Wolfram Syndrome protein, Miner1, regulates sulphydryl redox status, the unfolded protein response, and Ca2+ homeostasis. EMBO Mol. Med. *5*, 904–918. https://doi.org/10.1002/emmm.201201429.

Yu, J., Mao, S., Zhang, Y., Gong, W., Jia, Z., Huang, S., and Zhang, A. (2016). MnTBAP therapy attenuates renal fibrosis in mice with 5/6 nephrectomy. Oxid. Med. Cell. Longev. *2016*, 7496930. https://doi.org/10.1155/2016/7496930.

Zatyka, M., Da Silva Xavier, G., Bellomo, E.A., Leadbeater, W., Astuti, D., Smith, J., Michelangeli, F., Rutter, G.A., and Barrett, T.G. (2015). Sarco(endo)plasmic reticulum ATPase is a molecular partner of Wolfram syndrome 1 protein, which negatively regulates its expression. Hum. Mol. Genet. *24*, 814–827. https://doi.org/10.1093/hmg/ddu499.

Zatyka, M., Ricketts, C., da Silva Xavier, G., Minton, J., Fenton, S., Hofmann-Thiel, S., Rutter, G.A., and Barrett, T.G. (2008). Sodium-potassium ATPase 1 subunit is a molecular partner of Wolframin, an endoplasmic reticulum protein involved in ER stress. Hum. Mol. Genet. *17*, 190–200. https://doi.org/10.1093/hmg/ddm296.

Zhang, A., Williamson, C.D., Wong, D.S., Bullough, M.D., Brown, K.J., Hathout, Y., and Colberg-Poley, A.M. (2011). Quantitative proteomic analyses of human cytomegalovirus-induced restructuring of endoplasmic reticulum-mitochondrial contacts at late times of infection. Mol. Cell. Proteomics *10*. M111.009936. https://doi.org/10.1074/mcp.M111.009936.

Zorova, L.D., Popkov, V.A., Plotnikov, E.Y., Silachev, D.N., Pevzner, I.B., Jankauskas, S.S., Babenko, V.A., Zorov, S.D., Balakireva, A.V., Juhaszova, M., et al. (2018). Mitochondrial membrane potential. Anal. Biochem. *552*, 50–59. https://doi.org/10.1016/j.ab.2017.07.009.

Stem Cell Reports, Volume *18*

Supplemental Information

Depletion of WFS1 compromises mitochondrial function in hiPSC-derived neuronal models of Wolfram syndrome

Malgorzata Zatyka, Tatiana R. Rosenstock, Congxin Sun, Adina M. Palhegyi, Georgina W. Hughes, Samuel Lara-Reyna, Dewi Astuti, Alessandro di Maio, Axel Sciauvaud, Miriam E. Korsgen, Vesna Stanulovic, Gamze Kocak, Malgorzata Rak, Sandra Pourtoy-Brasselet, Katherine Winter, Thiago Varga, Margot Jarrige, Hélène Polvèche, Joao Correia, Eva-Maria Frickel, Maarten Hoogenkamp, Douglas G. Ward, Laetitia Aubry, Timothy Barrett, and Sovan Sarkar

SUPPLEMENTAL FIGURES

Figure S1

A hiPSC lines from which neural stem cells and neurons were generated

hiPSC line	Genotype	Mutations in *WFS1* gene
CT1	Healthy control	wild-type
CT2	Healthy control	wild-type
WS1	WS patient	c.1230-1233delCTCT [p.V412fs*440]; c.2171C>T [p.P724L]
WS2	WS patient	c.1230-1233delCTCT [p.V412fs*440]; c.1230-1233delCTCT [p.V412fs*440]
WS5	WS patient	c.1060-1062delTTC [p.F354del]; c.1676C>A [p.A559D]
WS5R	WS patient 'rescued'	WS5 corrected control after Dox treatment; *WFS1* cDNA under Dox promoter inserted at *AAVS1* locus

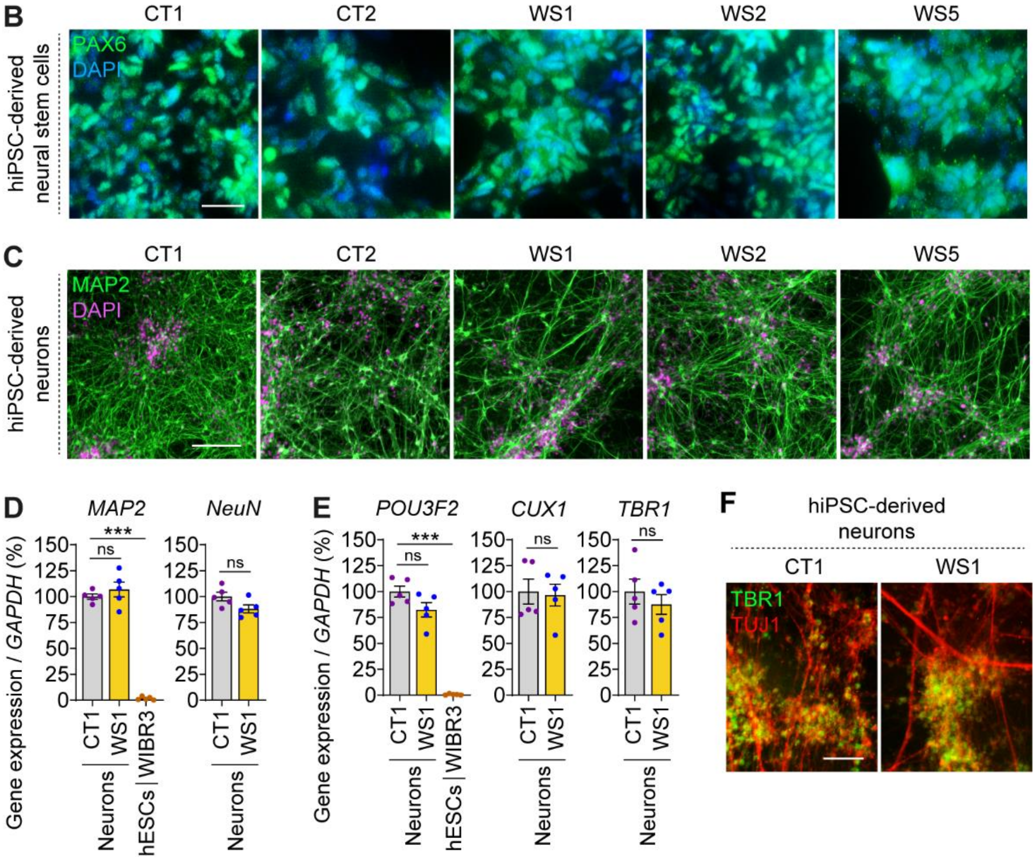

Figure S1. Characterization of hiPSC-derived neural stem cells and neurons; related to Figure 1.
(A) List of control (CT1 and CT2) and Wolfram syndrome (WS1, WS2 and WS5) patient-derived hiPSC lines from which neural stem cells (NSCs) and neurons were generated in this study.
(B, C) Immunofluorescence images of PAX6 (B) and MAP2 (C) in CT1, CT2, WS1, WS2 and WS5 hiPSC-derived NSCs (B) and 4 weeks (4 w) old neurons (C).
(D, E) qPCR expression analyses of *MAP2* (D), *NeuN* (D), *POU3F2* (E), *CUX1* (E) and *TBR1* (E) in CT1 and WS1 hiPSC-derived neurons (4 w); WIBR3 hESCs were used as a negative control (D, E).
(F) Immunofluorescence images of TBR1 and TUJ1 in CT1 and WS1 hiPSC-derived neurons (4 w).
Graphical data are mean ± s.e.m. of n = 5 biological replicates (D, E). P values were calculated by unpaired two-tailed Student's t-test (D, E; right and middle panels) or one-way ANOVA with Dunnett's multiple comparisons test (D, E; left panels) on 3 independent experiments. ***P<0.001; ns, non-significant. Scale bar, 25 µm (B), 50 µm (F), 100 µm (C).

Figure S2

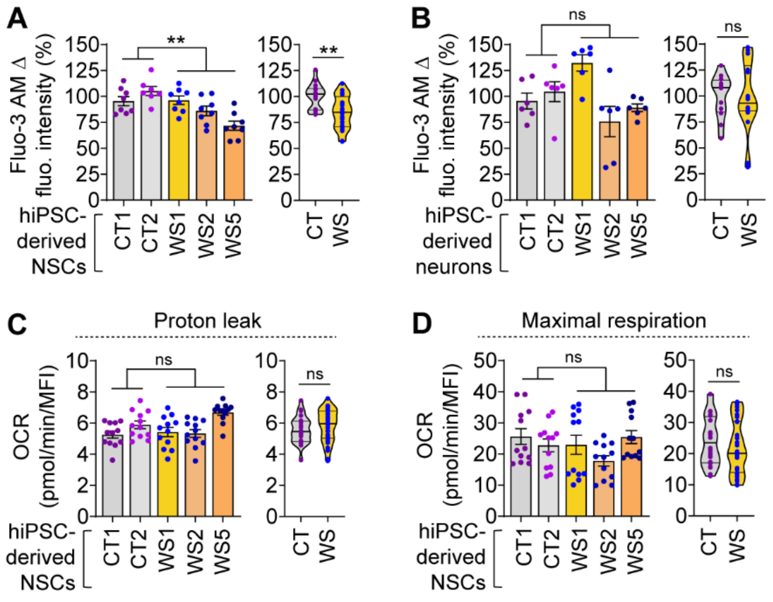

Figure S2. Analysis of mitochondrial Ca^{2+} and respiratory parameters in WS patient hiPSC-derived cells; related to Figure 2.

(A, B) Measurement of mitochondrial Ca^{2+} by Fluo-3 AM Δ fluorescence intensity in CT1, CT2, WS1, WS2 and WS5 hiPSC-derived NSCs (A) and neurons (4 w) (B).

(C, D) Oxygen consumption rate (OCR) levels were measured post mitochondrial stress test, involving oligomycin (Oligo), BAM15 and rotenone (Rot)/antimycin A (AA) treatment, in CT1, CT2, WS1, WS2 and WS5 hiPSC-derived NSCs, wherein proton leak (C) and maximal respiration (D) were calculated as described in experimental procedures and Table S6. MFI: mean fluorescent intensity. Graphical data are mean ± s.e.m. of n = 6–12 biological replicates as indicated (A–D), or displayed as violin plots (line at median) of CT and WS groups (A–D). P values were calculated by unpaired two-tailed Student's t-test on 2 (A, B) or 3 (C, D) independent experiments. **P<0.01; ns, non-significant.

Figure S3

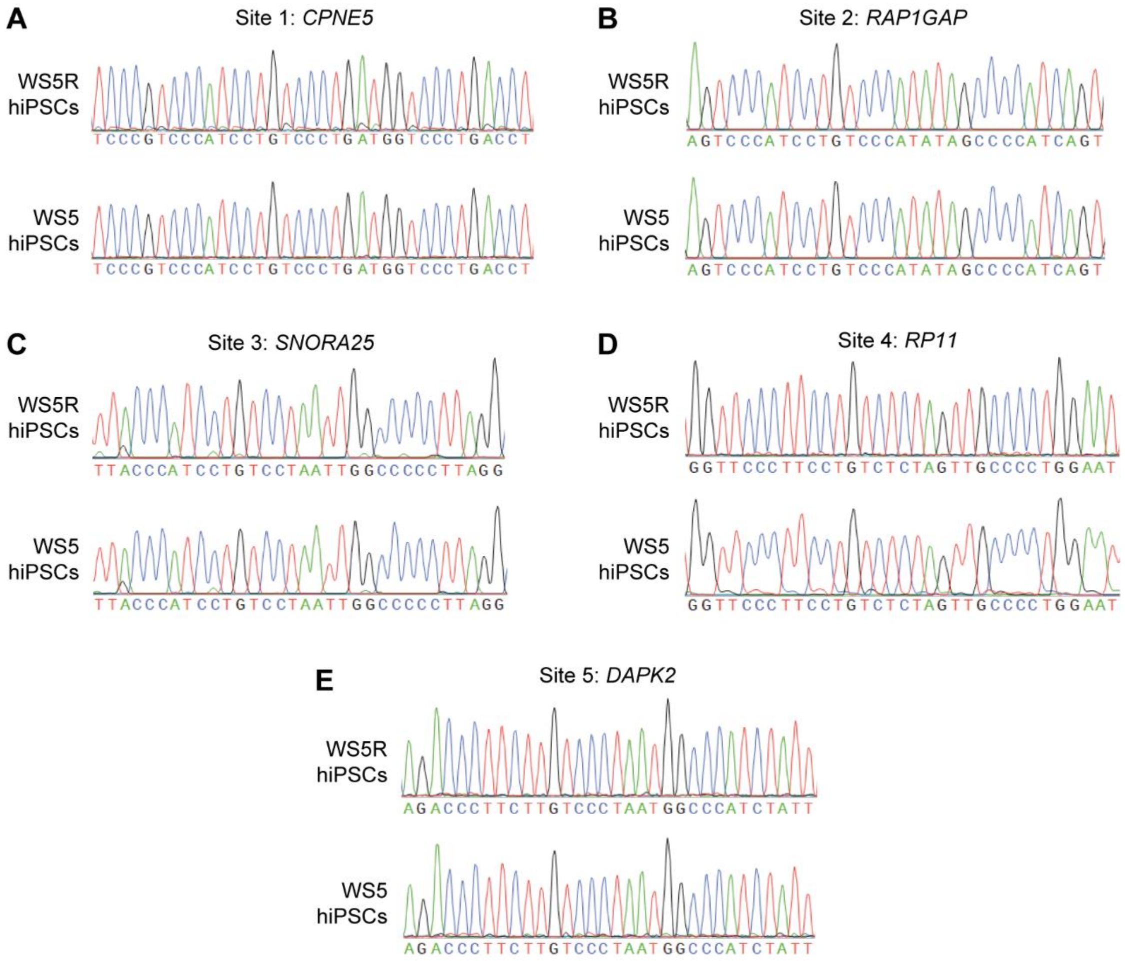

Figure S3. Off-target analysis in genome-edited WS5R hiPSC line; related to Figure 3.
(A–E) Potential off-target loci sequencing of top 5 sites, as determined by CRISPOR, illustrating no differences between WS5 and WS5R hiPSCs. Site 1: *CPNE5* (A); site 2: *RAP1GAP* (B); site 3: *SNORA25* (C); site 4: *RP11* (D); and site 5: *DAPK2* (E).

Figure S4

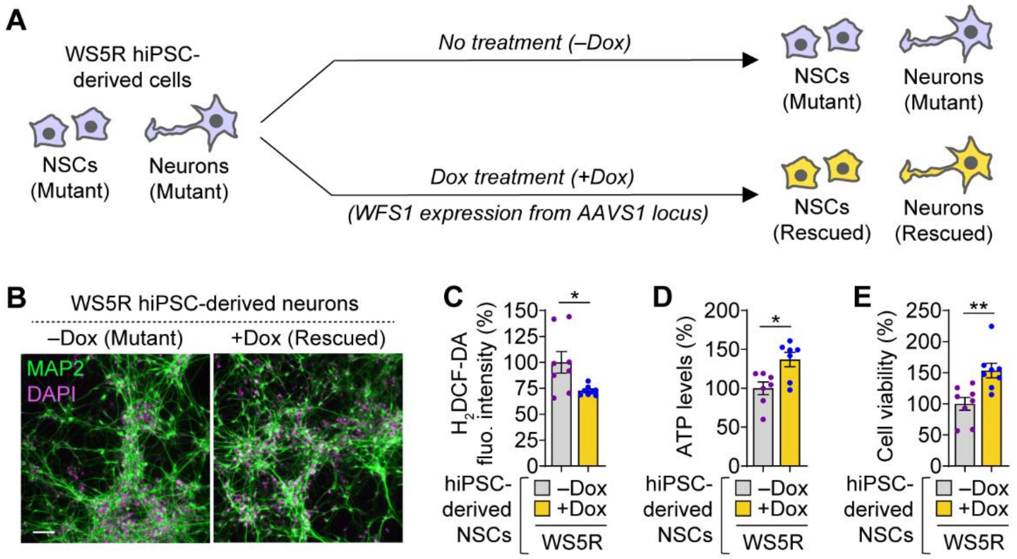

Figure S4. Genetic rescue of mitochondrial and cell death phenotypes in genome-edited WS patient hiPSC-derived cells; related to Figure 3.

(A) Genetic rescue in genome-edited WS5R hiPSC-derived neural stem cells (NSCs) and neurons, wherein WS5R cells treated with doxycycline (Dox) act as 'rescued' cells due to *WFS1* expression from *AAVS1* locus but WS5R cells in the absence of Dox remain as mutant cells.

(B) Immunofluorescence images of MAP2 in WS5R hiPSC-derived neurons (4 w), treated with or without 50 ng/mL Dox for 48 h.

(C–E) Measurements of ROS by H_2DCF-DA fluorescence intensity (C), ATP levels (D) and cell viability (E) in WS5R hiPSC-derived NSCs, treated with or without 50 ng/mL Dox for 48 h. Graphical data are mean ± s.e.m. of n = 7–8 biological replicates as indicated (C–E). P values were calculated by unpaired two-tailed Student's t-test on 3 independent experiments (C–E). *P<0.05; **P<0.01. Scale bar, 100 μm (B).

Figure S5

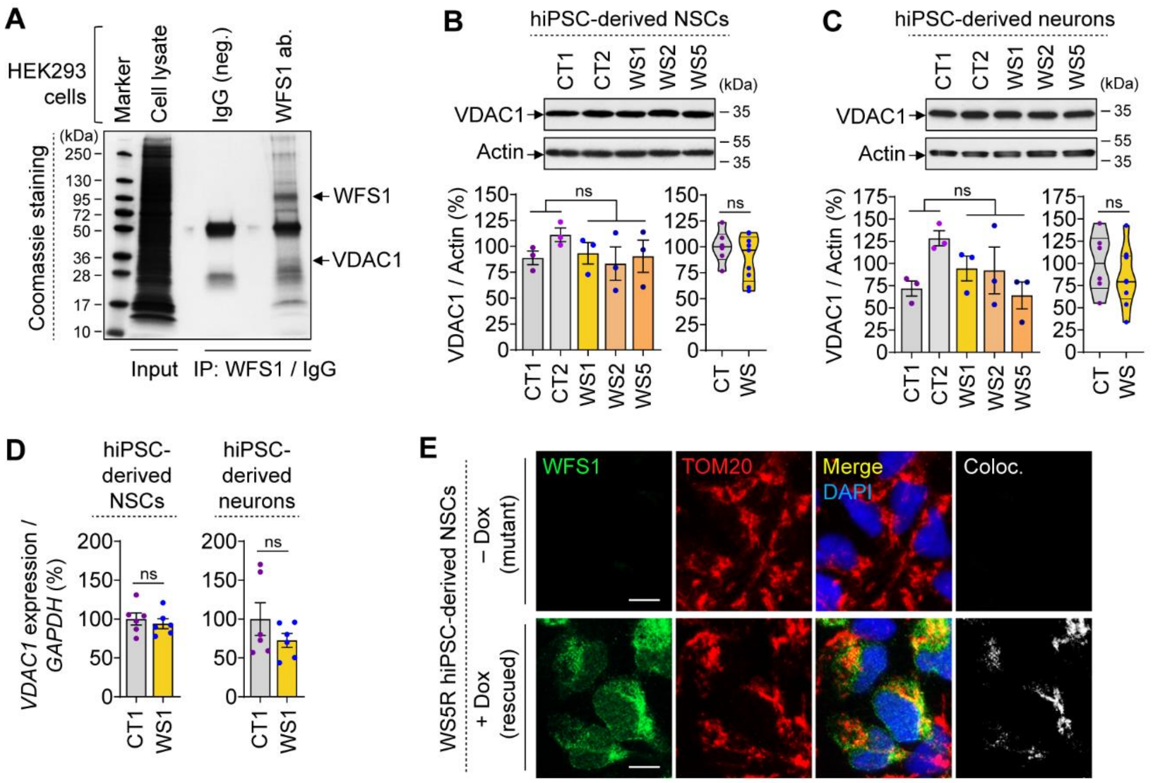

F Compounds used for modulating mitochondrial phenotype

Compounds	Cell type used	Property
Cyslosporin A (CsA)	hiPSC-derived NSCs & neurons	Inhibits mitochondrial permeability transition pore
MnTBAP	hiPSC-derived NSCs & neurons	Superoxide dismutase mimetic and superoxide scavenger
N-acetyl cysteine (NAC)	hiPSC-derived NSCs & neurons	Antioxidant and free radical scavenger

Figure S5. VDAC1 levels and WFS1–TOM20 colocalization in WS patient hiPSC-derived cells; related to Figure 4 and 6.
(A) Coomassie staining of SDS-PAGE gel with cell lysate (input) or after immunoprecipitation with WFS1 antibody or IgG (negative control) in HEK293 cells expressing Myc-WFS1. Approximate bands of WFS1 and VDAC1 are shown after pulldown.
(B, C) Immunoblotting and densitometric analyses of VDAC1 relative to Actin in CT1, CT2, WS1, WS2 and WS5 hiPSC-derived NSCs (B) and neurons (4 w) (C).
(D) qPCR expression analysis of *VDAC1* relative to *GAPDH* in CT1 and WS1 hiPSC-derived NSCs and neurons (4 w).
(E) Immunofluorescence images of TOM20 and WFS1 colocalization in WS5R hiPSC-derived NSCs, treated with or without 50 ng/ml Dox for 48 h.
(F) Details of compounds used for modulating mitochondrial phenotypes in WS hiPSC-derived NSCs and neurons.
Graphical data are mean ± s.e.m. of n = 3–6 biological replicates as indicated (B–D) or displayed as violin plots (line at median) of CT and WS groups (B, C). P values were calculated by unpaired two-tailed Student's t-test on 3 independent experiments (B–D). ns, non-significant. Scale bar, 10 µm (E).

SUPPLEMENTAL TABLES

Table S1

Compound name	Solvent	Stock conc.	Final conc.	Catalogue number	Source
Cyclosporin A (CsA)	DMSO	1 mg/mL	1 µM	SML1018	Merck
Mn(III)tetrakis(4-benzoic acid)porphyrin Chloride (MnTBAP)	NaOH, 80%	2 mM	2 µM	475870	Merck
N-Acetyl-L-cysteine (NAC)	H_2O	100 mM	100 µM	A9165	Merck

Table S1. List of compounds used for modulating mitochondrial function; related to Supplemental Experimental Procedures on compound treatment.
Details of compounds used in this study for modulating mitochondrial function in hiPSC-derived neural stem cells and neurons.

Table S2

Gene	Species	Primer direction	Primer sequence	Source	Reference
ATP5PD	Human	Forward	GCT GCT TTA CCT GAG AAT CCA CC	Merck	Origene (HP230531)
		Reverse	TAT CCT CTG GCA CGG GAA CCT T		
COX16	Human	Forward	TGG AGG TTC TTT TGG TCT TCG TG	Merck	Origene (HP212207)
		Reverse	TCT CAT ATT CCG ACT CTA AAG ATA TT		
CUX1	Human	Forward	TCC GTA GCA TCC AAG GCA GAC A	Merck	Origene (HP233477)
		Reverse	CTT CAT CAG AGC CAG TCT CCG A		
GAPDH	Human	Forward	GTC TCC TCT GAC TTC AAC AGC G	Merck	This study
		Reverse	ACC ACC CTG TTG CTG TAG CCA A		
MAP2	Human	Forward	AGG CCC AAG CTA AAG TTG GT	Merck	This study
		Reverse	ATG GTC CAC ACG GGC TTT AG		
NDUFA10	Human	Forward	TGG CTC AAG CAG GAC AAT CGC A	Merck	Origene (HP207839)
		Reverse	AGA CAC GGT CAG TCT GAT GAG C		
NeuN	Human	Forward	TAC AGC GAC AGT TAC GGC AG	Merck	This study
		Reverse	TTC CAA TGC TGT AGG TCG CC		
POU3F2	Human	Forward	GTG TTC TCG CAG ACC ACC ATC T	Merck	Origene (HP208713)
		Reverse	GCT GCG ATC TTG TCT ATG CTC G		
SDHB	Human	Forward	GCA GTC CAT AGA AGA GCG TGA G	Merck	Origene (HP206603)
		Reverse	TGT CTC CGT TCC ACC AGT AGC T		
TBR1	Human	Forward	TCA CTG GAG GTT TCA AGG AGG C	Merck	Origene (HP209514)
		Reverse	TTT CTT GGC GCA TCC AGT GAG C		
VDAC1	Human	Forward	GCA AAA TCC CGA GTG ACC CAG A	Merck	Origene (HP206903)
		Reverse	TCC AGG CAA GAT TGA CAG CGG T		

Table S2. List of primers for gene expression analysis; related to Supplemental Experimental Procedures on gene expression analysis.
Details of primers used in this study for gene expression analysis in hiPSC-derived neural stem cells and neurons.

Table S3

Antigen	Host species	Source	Catalogue number	Dilution
**Primary antibodies for immunoblotting**				
Actin	Mouse	Merck	A5441	1:2000
DRP1	Rabbit	Cell Signaling Technology	8570S	1:1000
c-Myc	Mouse	Merck	A5546	1:1000
OPA1	Rabbit	Cell Signaling Technology	67589S	1:1000
VDAC1	Mouse	Abcam	ab186321	1:1000
WFS1	Rabbit	Cell Signaling Technology	8749S	1:1000
WFS1	Sheep	R&D Systems	AF7417	1:1000
**Secondary antibodies for immunoblotting**				
Anti-mouse IgG, HRP-conjugated	Rabbit	Dako	P0161	1:10000
Anti-rabbit IgG, HRP-conjugated	Goat	Dako	P0448	1:10000
Anti-sheep IgG, HRP-conjugated	Donkey	R&D Systems	HAF016	1:2000
Anti-mouse IgG light chain, HRP-conjugated	Goat	Jackson Immuno Research	115-035-174	1:10000

Table S3. List of primary and secondary antibodies for immunoblotting; related to Supplemental Experimental Procedures on immunoblotting analysis.
Details of antibodies used in this study for immunoblotting analysis in hiPSC-derived neural stem cells and neurons and in HEK293 cells.

Table S4

Antigen	Host species	Source	Catalogue number	Dilution
**Primary antibodies for immunofluorescence**				
Calnexin	Rabbit	Abcam	ab22595	1:500
MAP2	Rabbit	Cell Signaling Technology	8707S	1:200
NESTIN	Rabbit	BioLegend	839801	1:500
PAX6	Rabbit	BioLegend	901301	1:100
TBR1	Rabbit	Cell Signaling Technology	49661S	1:250
TOM20	Mouse	Santa Cruz Biotechnology	sc17764	1:100
β3-Tubulin (TUJ1)	Mouse	Cell Signaling Technology	4466S	1:200
VDAC1	Mouse	Abcam	ab186321	1:100
WFS1	Rabbit	Cell Signaling Technology	8749S	1:80
**Secondary antibodies for immunofluorescence**				
Anti-mouse IgG (H+L), Alexa Fluor 594	Donkey	Thermo Fisher Scientific	A-21203	1:500
Anti-rabbit IgG (H+L), Alexa Fluor 488	Donkey	Thermo Fisher Scientific	A-21206	1:1000

Table S4. List of primary and secondary antibodies for immunofluorescence; related to Supplemental Experimental Procedures on immunofluorescence analysis.
Details of antibodies used in this study for immunofluorescence analysis in hiPSC-derived neural stem cells and neurons.

Table S5

Antigen	Host species	Source	Catalogue number	Concentration
**Primary antibodies for immunoprecipitation**				
WFS1	Rabbit	Cell Signaling Technology	8749S	0.007 µg/µL
Normal IgG	Rabbit	Cell Signaling Technology	2729S	0.007 µg/µL
Normal IgG	Rabbit	R&D Systems	AB105-c	0.007 µg/µL

Table S5. List of primary antibodies for immunoprecipitation; related to Supplemental Experimental Procedures on co-immunoprecipitation.
Details of antibodies used in this study for immunoprecipitation in hiPSC-derived neural stem cells and in HEK293 cells.

Table S6

Parameter	Equation
Basal respiration	(measurement before oligomycin stimulation) – (rate measurement after rotenone/antimycin A stimulation)
ATP production	(basal respiration) – (minimum rate measurement after oligomycin stimulation)
Proton leak	(minimum rate measurement after oligomycin stimulation) – (minimum measurement after rotenone/antimycin A stimulation)
Maximal respiration	(maximum rate measurement after BAM15 stimulation) – (minimum rate measurement after rotenone/antimycin A stimulation)

Table S6. Calculations for metabolic parameters in mitochondrial respiration measurements; related to Supplemental Experimental Procedures on mitochondrial respiration measurement. Details of calculations used in this study for assessing basal respiration, ATP production, proton leak and maximal respiration in hiPSC-derived neural stem cells and neurons.

Table S7

Gene	Species	Primer direction	Primer sequence	Source	Reference
CPNE5	Human	Forward	TGGACACCAAAGAATGGTGA	Eurofins	CRISPOR
		Reverse	CTGGCATCTGCAGGGTTATT		
RAP1GAP	Human	Forward	ATACCCACGCCCCTATACTC	Eurofins	CRISPOR
		Reverse	CTAGTGACAGCTGTGTGGTC		
SNORA25	Human	Forward	CGCGCCTCCCTTTATAAAGT	Eurofins	CRISPOR
		Reverse	ATGGTAGGCTCAGACACAGA		
RP11	Human	Forward	CCGGGAAATGTTATGAATGG	Eurofins	CRISPOR
		Reverse	TCACATGGAAGCACATGGAT		
DAPK2	Human	Forward	TGTGGAAATGGGGATGACTT	Eurofins	CRISPOR
		Reverse	CCTAAGCACCCGACAGAGAG		

Table S7. List of primers for off-target analysis; related to Supplemental Experimental Procedures on sequence analysis at off-targets.
Details of primers used in this study for off-target analysis in hiPSCs.

SUPPLEMENTAL EXPERIMENTAL PROCEDURES

Human pluripotent stem cell lines and culture

Human induced pluripotent stem cell (hiPSC) lines, used for generating neural stem cells (NSCs) and neurons, were established previously. These include control hiPSC lines, CT1 and CT2 (Pourtoy-Brasselet et al., 2021); and WS patient-derived hiPSC lines, WS1 (Shang et al., 2014), WS2 (Shang et al., 2014) and WS5 (Pourtoy-Brasselet et al., 2021). WS5R (rescued) hiPSC line was generated by genome editing via CRISPR/Cas9 mediated knock-in of *WFS1* cDNA under a doxycycline inducible promoter in the *AAVS1* locus of WS5 line, as previously established (Pourtoy-Brasselet et al., 2021). WIBR3 human embryonic stem cell (hESC) line was established previously (Lengner et al., 2010). The hiPSC and hESC lines were cultured, as previously described (Lengner et al., 2010; Pourtoy-Brasselet et al., 2021), on inactivated mouse embryonic fibroblast (MEF) feeder layer in hESC medium comprised of DMEM/F-12, 5 % KnockOut Serum Replacement, 1 % L-glutamine, 1 % non-essential amino acids, 1 % penicillin/streptomycin, 4 ng/mL human recombinant basic fibroblast growth factor (bFGF) (all from Gibco), 15 % fetal bovine serum (HyClone) and 0.1 mM β-mercaptoethanol (Sigma-Aldrich); or cultured feeder-free on Geltrex basement membrane matrix in StemFlex Basal Medium supplemented with StemFlex 10X Supplement (all from Gibco); and maintained in a humidified incubator with 5 % CO_2 and 5 % O_2 at 37 °C.

Generation and culture of hiPSC-derived neural stem cells

Neural stem cells (NSCs) were differentiated from hiPSCs, as described previously (Boissart et al., 2013; Pourtoy-Brasselet et al., 2021). NSCs were cultured on Poly-L-ornithine and Laminin (PO-L) (Sigma-Aldrich) coated plates or flasks in N2B27 medium comprised of DMEM/F-12 and Neurobasal medium in 1:1 ratio, 1 % N-2 supplement, 2 % B-27 supplement, 1% penicillin/streptomycin (all from Gibco), 0.1 % β-mercaptoethanol (Sigma-Aldrich) supplemented with 10 ng/mL FGF-2 (Miltenyi Biotec) and 10 ng/mL EGF (PeproTech), and were maintained in a humidified incubator with 5 % CO_2 at 37 °C. NSCs were passaged twice a week with 0.05 % Trypsin-EDTA (Gibco), and the medium was changed on alternate days. Approximately 6.5×10^4 cells/well were seeded in a 96-well plate for analyses of mitochondrial function or cell viability, 9×10^4 cells/well were seeded in 24-well plate with coverslips for immunostaining, and 4.5×10^5 cells/well were seeded in 6-well plate for immunoblotting and qPCR analyses. Analyses on NSCs were done after 48 h or after compound treatment.

Neuronal differentiation of hiPSC-derived NSCs

Neuronal differentiation of hiPSC-derived NSCs was carried out as described previously (Boissart et al., 2013; Pourtoy-Brasselet et al., 2021). The NSCs were seeded as above on PO-L coated plates in N2B27 medium without FGF-2 and EGF, and were maintained in a humidified incubator with 5 % CO_2 at 37 °C. At day 4 of neuronal differentiation, cells were treated with 10 µM DAPT (Tocris) to prevent cell proliferation. The N2B27 medium (without FGF-2 and EGF) was changed every 2 days and neuronal differentiation was carried out for 4 weeks (4 w), after which analyses were done for cellular identity, mitochondrial function and cell viability. The neurons generated *in vitro* were cortical in nature (Boissart et al., 2013; Pourtoy-Brasselet et al., 2021).

HEK293 cell culture and transfection

HEK293 cells were cultured in DMEM medium supplemented with 10 % FBS, 1 % L-glutamine, 1 % non-essential amino acids and 1 % penicillin/streptomycin (all from Gibco), and were maintained in a humidified incubator with 5 % CO_2 at 37 °C. The cells were plated at 5×10^5 cells per well in 6-well plates, and after 24 h, transient transfection was performed with FuGENE HD Transfection Reagent (Promega) using plasmids pCMV-Myc-WFS1 and pCMV-Myc (Zatyka et al., 2008) according to the manufacturer's protocol. Briefly, 3 µg of the plasmid was used per well with 3:1 ratio of FuGENE HD to DNA. Cells were incubated with the transfection mix for 48 h after which the samples were harvested for experimentation.

Compound treatment

For restoration of WFS1 levels, WS5R hiPSC-derived NSCs and neurons were treated with 50 ng/mL doxycycline (Dox; Sigma-Aldrich) for 48 h to induce WFS1 expression (Pourtoy-Brasselet et al., 2021). For modulating mitochondrial function, WS1 or WS2 hiPSC-derived NSCs and neurons were treated with 1 µM Cyclosporin A (CsA), 2 µM Mn(III)tetrakis(4-benzoic acid)porphyrin Chloride (MnTBAP), and 100 µM N-Acetyl-L-cysteine (NAC) (all from Sigma-Aldrich; details of the drugs in Table S1) for 48 h (in NSCs with replenishment on the first day) or for 6 days (in neurons with replenishment on the third day).

RNA-seq data analysis
AmpliSeq data used in this study has been previously published and deposited on NCBI GEO under accession number GSE156911 (Pourtoy-Brasselet et al., 2021). A subset of genes related to mitochondria were established from several pathways (WikiPathway 2021 Human / WP111; HumanCyc 2016 / PWY66-407; Reactome 2016 / R-HSA-163200) combined with Gene Ontologies (GO:0006084; GO:0010510; GO:0006390; GO:0030150; GO:0005741; GO:0000266; GO:0048311) followed by web-based gene list enrichment analysis tool EnrichR (Chen et al., 2013; Kuleshov et al., 2016). Commonly expressed upregulated and downregulated differentially expressed genes (DEGs) (P value ≤ 5 %; Fold change ≥ 1.5; Minimum reads > 100) between mitochondria gene-set of control and WS NSCs or neurons were selected using Venny diagram (v2.1.0) (Oliveros, 2007-2015). These mitochondria-associated DEGs in NSCs and neurons were depicted in a volcano plot by plotting the magnitude of change [Log_2(Fold change)] against the measure of significance [Log_{10}(P adjusted)].

Gene expression analysis
The expression of mitochondrial and neuronal genes was analyzed by quantitative real-time PCR (qPCR), as previously described (Araujo et al., 2020; Hummon et al., 2007). Briefly, total RNA was extracted using Trizol (Invitrogen), and the quality and concentration of mRNA was evaluated using NanoDrop ND-1000 spectrophotometer (Thermo Fisher Scientific). The RNA was converted to complementary DNA (cDNA) using iScript cDNA Synthesis Kit (Bio-Rad) according to manufacturer's instruction. 100 ng of cDNA was used for qPCR with 300 nM of gene-specific primers (primers sequences listed in Table S2) and SsoAdvanced Universal SYBR Green Supermix (Bio-Rad) using the Applied Biosystems QuantStudio 5 Real-Time PCR System (Thermo Fisher Scientific). The reaction comprised an initial cycle of 10 min at 95 °C, and then 40 subsequent cycles of 15 sec at 95 °C and 60 secs at 60 °C, followed by melting curve of 95 °C for 15 sec, 60 °C for 1 min and 95 °C for 15 sec. Data were analyzed using $2^{-\Delta\Delta Ct}$ method, normalized to the expression of the housekeeping gene *GAPDH* and expressed as a percentage of the control condition.

Immunoblotting analysis
For WFS1 immunoblotting, cells were lysed in Lysis Buffer [62.5 mM Tris-HCl pH 6.8, 2 % SDS, 12.5 % glycerol (all from Sigma-Aldrich) and Complete Mini Protease Inhibitor Cocktail (Roche)]. For all other immunoblotting, cells were lysed in RIPA Buffer [50 mM Tris pH 8, 150 mM NaCl, 0.1 % SDS, 1 mM EDTA, 0.5 % deoxycholate, 1 % IGEPAL (all from Sigma-Aldrich) and Complete Mini Protease Inhibitor Cocktail (Roche)]. The cell lysates were sonicated 3 × 10 s followed by centrifugation at 12000 × *g* for 30 min at 4 ºC. Protein concentration was measured by DC Protein Assay (Bio-Rad). The samples were boiled for all immunoblotting but not for WFS1 immunoblotting. 20 µg of protein per sample was subjected to SDS–PAGE and immunoblot analysis as described previously (Gharanei et al., 2013; Seranova et al., 2019; Sun et al., 2021). The immunoblots were then incubated in Blocking Buffer (5 % non-fat milk powder in PBS-Tween 20) for 1 h at room temperature, followed by incubation in primary antibodies overnight at 4 °C and then in appropriate secondary antibodies conjugated to horseradish peroxidase for 1 h at room temperature (list of primary and secondary antibodies for immunoblotting analysis in Table S3). The chemiluminescent signal was visualized using SuperSignal West Femto Maximum Sensitivity Substrate (Thermo Fisher Scientific) or Amersham ECL Western Blotting Detection Reagent (GE Healthcare) on Amersham Hyperfilm ECL (GE Healthcare) via ECOMAX X-ray Film Processor (PROTEC). Densitometry analysis on immunoblots was done using ImageJ v1.48 (NIH) software. The data was expressed as a percentage of the control condition, as previously described (Seranova et al., 2019; Sun et al., 2021).

Immunofluorescence analysis
Immunofluorescence analysis was performed as described previously (Seranova et al., 2019; Sun et al., 2021). Briefly, cells were washed in PBS, fixed with 4 % formaldehyde (Thermo Fisher Scientific) at room temperature for 15 min, permeabilized with 0.5 % Triton X-100 (Sigma-Aldrich) for 10 min, and incubated with Blocking Buffer [5 % donkey serum (Sigma-Aldrich) in PBS] for 1 h at room temperature. The cells were then incubated overnight with primary antibodies (diluted in Blocking Buffer) at 4 °C, then washed in PBS, followed by incubation with appropriate Alexa Fluor conjugated secondary antibodies (diluted in Blocking Buffer) for 1 h at room temperature (list of primary and secondary antibodies for immunofluorescence analysis in Table S4), and washed again in PBS. The coverslips were mounted on glass slides with ProLong Gold antifade reagent with DAPI (Invitrogen).

Staining of mitochondria-associated ER membranes (MAMs)
Analysis of MAMs by staining of mitochondria (with MitoTracker Red CMXRos dye) and ER (with Calnexin antibody) was done as described previously (Wang et al., 2021). The hiPSC-derived NSCs

were seeded into an initial density of 1–2×10^5 cells per well in 24-well plates with PO-L-coated cover slips. Neuronal differentiation was done for 4 weeks. The hiPSC-derived neurons were treated with or without 50 ng/mL doxycycline for 48 h. Cells were then incubated with 1 µM MitoTracker Red CMXRos (Invitrogen) in a humidified incubator with 5 % CO_2 at 37 °C, followed by immunostaining with Calnexin antibody and staining with 10 µg/mL DAPI (Invitrogen). The cover clips were mounted on Vectashield antifade mountant (Vector Laboratories).

Image acquisition of fixed cells

Immunofluorescence images of fixed cells were acquired by fluorescence microscopy using EVOS FL Cell Imaging System (Thermo Fisher Scientific) with AMG 10x Plan FL and AMG 40x Plan FL lens, or with Zeiss LSM880 confocal microscope with Airyscan (Zeiss) equipped with a 100x/1.4 PlanApo objective. Confocal microscope images were acquired and processed with the Zeiss ZEN (Black) software. For analyses of MAMs and mitochondrial branch length, images of fixed cells were acquired by structured illumination microscopy (SIM) using a Nikon N-SIM System (Nikon Instruments) equipped with a SR HP Apo TIRF 100x/1.49 objective and two Hamamatsu ORCA Flash4 CMOS cameras. The Nikon NIS Element software was used to acquire and process SIM images for analysis. The colocalization and colour threshold panels of images were generated using ImageJ v1.48 (NIH) software.

Image analysis for quantification of MAMs

For quantification of MAMs, colocalization analysis between MitoTracker Red CMXRos and Calnexin was performed on SIM reconstructed images using JacCoP plugin (https://imagej.net/plugins/jacop) within the Fiji/ImageJ software. The JACoP plugin allows to use several commonly used colocalization indicators. Among those, Mander's coefficient was used because it shows the percentage of pixel that overlaps between two channels (Dunn et al., 2011), therefore quantifying the colocalization between mitochondria and ER. The coefficient values range between 0 and 1, expressing the ratio of intensity of positive pixels (or threshold) from one channel into another one. Image analysis for the quantification of MAMs was performed on ~75 images per sample, and the data on Mander's coefficient was expressed as a percentage. The cells imaged for MAM analysis were selected based on their neuronal morphology.

Mitochondrial branch length analysis

Mitochondrial branch length analysis after MitoTracker Red CMXRos staining (as described above) was done, as previously described (Valente et al., 2017), on SIM reconstructed images. Measurements of mitochondrial branch length, mitochondrial summed branch length and mitochondrial footprint were done in hiPSC-derived neurons (~50 images per sample) using Analyze Morphology plugin from Mitochondrial Network Analysis (MiNA) toolset in Fiji v2.9.0 (Schindelin et al., 2012) with the following settings: (i) Median filter: radius = 4; (ii) Unsharp mask: radius = 3, mask weight = 0.7; (iii) CLAHE: blocksize = 99, histogram bins = 200, max slope = 3, mask = none. Otsu Thresholding and Ridge Detection were selected with the following settings: High contrast = 250; Low contrast = 140; Line width = 15; Minimum line length = 1. The hiPSC-derived neurons were identified based on their morphology, and non-neuronal cells or artifacts were removed using a mask in ImageJ prior to MiNA analysis.

Co-immunoprecipitation

HEK293 cell lysate (600 µg) overexpressing either pCMV-Myc-WFS1 (Myc-WFS1) or pCMV-Myc (empty Myc), or WS5R hiPSC-derived NSC lysate in the presence or absence of Doxycycline, was used for co-immunoprecipitation (co-IP) with 2.35 µg of either WFS1 antibody (rabbit) or rabbit IgG (two different rabbit IgG were used) from non-immunised animals (list of primary antibodies for immunoprecipitation in Table S5). Co-IP was performed with Dynabeads Protein A Immunoprecipitation Kit (Invitrogen) as per manufacturer's instructions with minor modifications. The cell lysates mixed with antibody and magnetic beads were incubated for 30 min at room temperature on a rotation wheel followed by 6 washes in wash buffer. The elution samples (IP samples) as well as the whole cell lysates (inputs) were subjected to immunoblotting analysis with VDAC1 (mouse) antibody, followed by reprobing of the immunoblot with WFS1 (sheep) and c-Myc (mouse) antibodies (list of primary and secondary antibodies for immunoblotting analysis in Table S3).

Identification of WFS1 interactors by immunoprecipitation and mass spectrometry

HEK293 cell lysate (1 mg protein) overexpressing Myc-WFS1 (Zatyka et al., 2008) was used for immunoprecipitation (IP) with 5 µg of WFS1 antibody (rabbit) or with 5 µg of rabbit IgG from non-immunised animals (used as a negative control). The Dynabeads Protein A Immunoprecipitation Kit

(Invitrogen) was used for IP following the manufacturer's protocol. The immunoprecipitated proteins were eluted in SDS loading buffer by heating at 95 °C for 10 min, separated by SDS–PAGE on a 4–20 % Mini-Protean Precast Protein Gel (Bio-Rad), followed by staining with Bio-Safe Coomassie Stain (Bio-Rad) for 1 h and then de-staining in water overnight. The protein bands were cut from the stained gel and subjected to in-gel digestion using sequencing grade trypsin (Promega). The peptides were extracted with 1 % formic acid in 10 % acetonitrile for 60 min at room temperature and then with 2 % formic acid in 60 % acetonitrile for 30 min at room temperature, and lyophilised. The peptides were dissolved in 0.1 % formic acid. The resulting peptides were analysed by LC-MS/MS using a 60 min 0–40 % acetonitrile gradient in 0.1 % formic acid (75 μm × 25 cm C18 Pepmap column, Dionex) and a Bruker Impact Q-ToF Mass Spectrometer (Bruker Daltonics). Peptides were identified using MASCOT to search the SWISSPROT human protein sequence database. Mass tolerances for parent and fragment ions were 20 p.p.m. and 0.05 Da, respectively, and the minimum peptide MOWSE score was 25. Protein identifications were filtered using both a 1 % false discovery threshold and a requirement for two or more peptides using ProteinScape software (Bruker Daltonics). Mass spectrometry data for WFS1 interactors has been deposited in MassIVE repository, accession number MSV000091646.

Mitochondrial $\Delta\Psi_m$, ROS and Ca^{2+} measurements
Measurements of mitochondrial membrane potential ($\Delta\Psi_m$), reactive oxygen species (ROS) and mitochondrial Ca^{2+} were respectively done using TMRE (Tetramethylrhodamine ethyl ester), CM-H_2DCF-DA (chloromethyl derivative of 2',7'-dichlorodihydrofluorescein diacetate) and Fluo-3 AM (all from Invitrogen), as described previously (Araujo et al., 2020; Rosenstock et al., 2022; Silva et al., 2019). Briefly, cells were loaded with Microscopy Medium (120 mM NaCl, 3.5 mM KCl, 0.4 mM KH_2PO_4, 5 mM $NaHCO_3$, 1.2 mM $NaSO_4$, 20 mM HEPES and 15 mM glucose; pH 7.4) supplemented with 1 mM $CaCl_2$ (all from Sigma-Aldrich), along with 1 % Pluronic F-127 (Thermo Fisher Scientific) for Fluo-3 AM only, and incubated with 500 nM TMRE (for $\Delta\Psi_m$), 20 µM CM-H_2DCFDA (for ROS) or 10 µM Fluo-3 AM (for mitochondrial Ca^{2+}) for 1 h at 37 ºC. The fluorescence signals of TMRE, CM-H_2DCFDA and Fluo-3 AM were acquired using EnSpire Multimode microplate reader (Perkin Elmer) for a period of 5 min to get basal fluorescence, and again for TMRE and Fluo-3 AM for another 5 min after the addition of the mitochondrial uncoupler, 10 µM FCCP (fluorocarbonyl cyanide phenylhydrazone; Sigma-Aldrich). The delta (Δ) values of TMRE and Fluo-3 AM, denoting mitochondrial $\Delta\Psi_m$ and mitochondrial Ca^{2+}, were calculated by subtracting the basal fluorescence from post-FCCP fluorescence (Araujo et al., 2020; Rosenstock et al., 2022; Silva et al., 2019). Data were obtained as relative fluorescence units, normalised to protein concentration by Bradford Protein Assay (Bio-Rad) and expressed as a percentage of the control condition.

ATP measurement
ATP levels were measured using ApoSENSOR ADP/ATP Ratio Bioluminescent Assay Kit (BioVision) as per manufacturer's instructions (Rosenstock et al., 2022). Briefly, the Reaction Mix containing Nucleotide Releasing Buffer and ATP monitoring enzyme was added into a 96-well, white-walled, clear flat-bottom optical plate and incubated at room temperature for 1 h to burn the residual ATP levels. The luminescence was then measured using EnSpire Multimode microplate reader (Perkin Elmer) to determine the background reading (Data A). Cells cultured in a separate 96-well plate were incubated with Nucleotide Releasing Buffer for 5 min at room temperature to release the ATP. The supernatant was then transferred to the appropriate wells of the 96-well white-walled plate containing the Reaction Mix, and incubated for 2 min at room temperature. The luminescence was measured again using microplate reader (Data B). ATP levels were determined by subtracting Data B from Data A, normalized to protein concentration by Bradford Protein Assay (Bio-Rad), and expressed as a percentage of control condition.

Mitochondrial respiration measurement
The hiPSC-derived NSCs were seeded into an initial density of 4–4.5×10^4 cells per well in XF96 cell-culture microplates previously coated with PO-L. The NSCs were cultured for 48 h (for measurement in NSCs) whilst the neurons were generated from NSCs after differentiation for 4 weeks (for measurement in neurons), amounting to ~3.5×10^4 cells per well in XF96 cell-culture microplates. Before the experiment, the original culture medium was replaced with Seahorse XF DMEM medium without phenol red supplemented with 2.5 mM L-glutamine, 0.5 mM sodium pyruvate and 17.5 mM glucose (all from Agilent; supplemented to match the levels of these components in the DMEM/F-12 medium in which the neuronal cells were cultured), and the cells were incubated for 1 h in a non-CO_2 incubator. Preparation of all the regents was done while the cells were in the incubation period and following the manufacturer's instructions. Basal levels of oxygen consumption rates (OCR) were measured on an XFe96 Extracellular Flux Analyzer (Agilent). Cells were stimulated with 2 µM

oligomycin, 3 µM BAM15 and 1 µM rotenone/antimycin A (all from Sigma-Aldrich), following the instructions specified in the XF Cell Mito Stress Test Kit (Agilent). A range of metabolic parameters were calculated, such as basal respiration, ATP production, proton leak and maximal respiration (calculations described in Table S6). CyQUANT Direct Cell Proliferation Assay (Invitrogen) was used to normalise cell number following the manufacturer's instructions. Fluorescence was measured in a FLUOstar Omega Plate Reader (BMG Labtech). We also utilised a protein normalisation method with Bradford Protein Assay (Bio-Rad) to corroborate that our normalisation method was accurate. The saturation dynamics for Seahorse was not at maximum level, and the levels were within a dynamic range. Biological replicates represent each well of the Seahorse plates, arising from 3 independent experiments performed on different days whilst keeping the same conditions as the other days.

Cell viability measurement by cytotoxicity assay
Cell viability was measured by luminescence-based CytoTox-Glo Cytotoxicity Assay (Promega) as per manufacturer's protocol. This luminescence-based cytotoxicity assay measures the extracellular activity of a distinct dead-cell protease when it is released from membrane-compromised cells. Briefly, cells in 96-well, white-walled, clear flat-bottom optical plates were incubated with CytoTox-Glo Assay Reagent (comprising of Assay Buffer and AAF-Glo Substrate) for 15 min at room temperature in the dark. The luminescence was measured using EnSpire Multimode microplate reader (Perkin Elmer). The readings obtained were attributed to the basal cytotoxicity (first reading). Cells were further incubated with Lysis Reagent (comprising of Assay Buffer and Digitonin) for 30 min at room temperature in the dark, after which luminescence was measured again (second reading). Cell viability was determined by subtracting the first reading from the second reading according to manufacturer's instructions, and expressed as percentage of the control condition.

TUNEL assay for apoptotic cells
Cells were stained with Click-iT Plus TUNEL Assay for *in situ* apoptosis detection, Alexa Fluor 488 dye (Invitrogen), as per manufacturer's protocol. Briefly, cells were fixed with 4 % formaldehyde (Thermo Fisher Scientific) for 15 min, permeabilized with 0.25 % Triton X-100 (Sigma-Aldrich) for 20 min at room temperature and washed with deionized water. Cells were incubated in TdT reaction buffer for 10 min at 37 °C, further incubated in TdT reaction mixture (comprising of TdT reaction buffer, EdUTP and TdT enzyme) for 60 min at 37 °C, washed with 3 % BSA (in PBS), then incubated in Click-iT Plus TUNEL reaction cocktail for 30 min at 37 °C, and washed again with 3 % BSA. To detect TUNEL$^+$ apoptotic nuclei only in neurons, cells were subjected to immunofluorescence by blocking with 3 % BSA, incubating with TUJ1 antibody (in 3% BSA) overnight at 4 °C, and then incubating with Alexa Fluor 594 secondary antibody for 1 h at room temperature. Coverslips were mounted on glass slides with ProLong Gold antifade reagent with DAPI (Invitrogen), followed by analysis by fluorescence microscopy as previously described (Maetzel et al., 2014). The percentage of TUNEL$^+$ nuclei was calculated from the total number of TUJ1$^+$ cells analysed. Approximately 200–300 cells per sample were analysed.

Sequence analysis at off-targets
Genomic DNA was extracted from WS5 and WS5R hiPSCs using QIAamp DNA Blood Kits (Qiagen). PCR reactions were carried out with 50 ng genomic DNA using the Phusion High-Fidelity DNA Polymerase Kit (Thermo Fisher Scientific) as per manufacturer's instruction (primers sequences are listed in Table S7). PCR conditions were 30 s at 98°C, followed by 30 cycles of 10 s at 98 °C, 30 s at 63 °C and 15 s at 72 °C, then 5 min at 72 °C. PCR products were sent for sequencing to Genewiz. To determine possible off-target loci, CRISPOR tool was used and the top five loci were sequenced.

Statistical analysis
Graphical data are shown from 3 or more biological replicates from independent experiments, as indicated in the respective figure legends. Graphical data are depicted by column graph scatter dot plot (mean ± s.e.m.) or violin plot (line at median) using Prism v8.3.1 software (GraphPad). Quantification of data are described under various Methods sections where appropriate. Statistical significance (P value) on graphical data was determined using unpaired two-tailed Student's t-test or by one-way ANOVA with Tukey's or Dunnett's multiple comparisons test using Prism v8.3.1 software (GraphPad). *$P<0.05$; **$P<0.01$; ***$P<0.001$; ns, non-significant.

SUPPLEMENTAL REFERENCES

Araujo, B.G., Souza, E.S.L.F., de Barros Torresi, J.L., Siena, A., Valerio, B.C.O., Brito, M.D., and Rosenstock, T.R. (2020). Decreased mitochondrial function, biogenesis, and degradation in peripheral blood mononuclear cells from amyotrophic lateral sclerosis patients as a potential tool for biomarker research. Mol Neurobiol *57*, 5084-5102. 10.1007/s12035-020-02059-1.

Boissart, C., Poulet, A., Georges, P., Darville, H., Julita, E., Delorme, R., Bourgeron, T., Peschanski, M., and Benchoua, A. (2013). Differentiation from human pluripotent stem cells of cortical neurons of the superficial layers amenable to psychiatric disease modeling and high-throughput drug screening. Transl Psychiatry *3*, e294. 10.1038/tp.2013.71.

Chen, E.Y., Tan, C.M., Kou, Y., Duan, Q., Wang, Z., Meirelles, G.V., Clark, N.R., and Ma'ayan, A. (2013). Enrichr: interactive and collaborative HTML5 gene list enrichment analysis tool. BMC Bioinformatics *14*, 128. 10.1186/1471-2105-14-128.

Dunn, K.W., Kamocka, M.M., and McDonald, J.H. (2011). A practical guide to evaluating colocalization in biological microscopy. Am J Physiol Cell Physiol *300*, C723-742. 10.1152/ajpcell.00462.2010.

Gharanei, S., Zatyka, M., Astuti, D., Fenton, J., Sik, A., Nagy, Z., and Barrett, T.G. (2013). Vacuolar-type H+-ATPase V1A subunit is a molecular partner of Wolfram syndrome 1 (WFS1) protein, which regulates its expression and stability. Hum Mol Genet *22*, 203-217. 10.1093/hmg/dds400.

Hummon, A.B., Lim, S.R., Difilippantonio, M.J., and Ried, T. (2007). Isolation and solubilization of proteins after TRIzol extraction of RNA and DNA from patient material following prolonged storage. Biotechniques *42*, 467-470, 472. 10.2144/000112401.

Kuleshov, M.V., Jones, M.R., Rouillard, A.D., Fernandez, N.F., Duan, Q., Wang, Z., Koplev, S., Jenkins, S.L., Jagodnik, K.M., Lachmann, A., et al. (2016). Enrichr: a comprehensive gene set enrichment analysis web server 2016 update. Nucleic Acids Res *44*, W90-97. 10.1093/nar/gkw377.

Lengner, C.J., Gimelbrant, A.A., Erwin, J.A., Cheng, A.W., Guenther, M.G., Welstead, G.G., Alagappan, R., Frampton, G.M., Xu, P., Muffat, J., et al. (2010). Derivation of pre-X inactivation human embryonic stem cells under physiological oxygen concentrations. Cell *141*, 872-883. 10.1016/j.cell.2010.04.010.

Maetzel, D., Sarkar, S., Wang, H., Abi-Mosleh, L., Xu, P., Cheng, A.W., Gao, Q., Mitalipova, M., and Jaenisch, R. (2014). Genetic and chemical correction of cholesterol accumulation and impaired autophagy in hepatic and neural cells derived from Niemann-Pick Type C patient-specific iPS cells. Stem Cell Reports *2*, 866-880. 10.1016/j.stemcr.2014.03.014.

Oliveros, J.C. (2007-2015). Venny. An interactive tool for comparing lists with Venn’s diagrams. http://bioinfogp.cnb.csic.es/tools/venny/index.html.

Pourtoy-Brasselet, S., Sciauvaud, A., Boza-Moran, M.G., Cailleret, M., Jarrige, M., Polveche, H., Polentes, J., Chevet, E., Martinat, C., Peschanski, M., and Aubry, L. (2021). Human iPSC-derived neurons reveal early developmental alteration of neurite outgrowth in the late-occurring neurodegenerative Wolfram syndrome. Am J Hum Genet *108*, 2171-2185. 10.1016/j.ajhg.2021.10.001.

Rosenstock, T.R., Sun, C., Hughes, G.W., Winter, K., and Sarkar, S. (2022). Analysis of mitochondrial dysfunction by microplate reader in hiPSC-derived neuronal cell models of neurodegenerative disorders. Methods Mol Biol. *2549*, 1-21. 10.1007/7651_2021_451.

Schindelin, J., Arganda-Carreras, I., Frise, E., Kaynig, V., Longair, M., Pietzsch, T., Preibisch, S., Rueden, C., Saalfeld, S., Schmid, B., et al. (2012). Fiji: an open-source platform for biological-image analysis. Nat Methods *9*, 676-682. 10.1038/nmeth.2019.

Seranova, E., Ward, C., Chipara, M., Rosenstock, T.R., and Sarkar, S. (2019). In vitro screening platforms for identifying autophagy modulators in mammalian cells. Methods Mol Biol *1880*, 389-428. 10.1007/978-1-4939-8873-0_26.

Shang, L., Hua, H., Foo, K., Martinez, H., Watanabe, K., Zimmer, M., Kahler, D.J., Freeby, M., Chung, W., LeDuc, C., et al. (2014). beta-cell dysfunction due to increased ER stress in a stem cell model of Wolfram syndrome. Diabetes *63*, 923-933. 10.2337/db13-0717.

Silva, L.F.S.E., Brito, M.D., Yuzawa, J.M.C., and Rosenstock, T.R. (2019). Mitochondrial dysfunction and changes in high-energy compounds in different cellular models associated to hypoxia: Implication to schizophrenia. Sci Rep *9*, 18049. 10.1038/s41598-019-53605-4.

Sun, C., Rosenstock, T.R., Cohen, M.A., and Sarkar, S. (2021). Autophagy dysfunction as a phenotypic readout in hiPSC-derived neuronal cell models of neurodegenerative diseases. Methods Mol Biol. *2549*, 103-136. 10.1007/7651_2021_420.

Valente, A.J., Maddalena, L.A., Robb, E.L., Moradi, F., and Stuart, J.A. (2017). A simple ImageJ macro tool for analyzing mitochondrial network morphology in mammalian cell culture. Acta Histochem *119*, 315-326. 10.1016/j.acthis.2017.03.001.

Wang, C., Dai, X., Wu, S., Xu, W., Song, P., and Huang, K. (2021). FUNDC1-dependent mitochondria-associated endoplasmic reticulum membranes are involved in angiogenesis and neoangiogenesis. Nat Commun *12*, 2616. 10.1038/s41467-021-22771-3.

Zatyka, M., Ricketts, C., da Silva Xavier, G., Minton, J., Fenton, S., Hofmann-Thiel, S., Rutter, G.A., and Barrett, T.G. (2008). Sodium-potassium ATPase 1 subunit is a molecular partner of Wolframin, an endoplasmic reticulum protein involved in ER stress. Hum Mol Genet *17*, 190-200. 10.1093/hmg/ddm296.
